# Comparison of the diagnostic accuracy of enhanced-CT and double contrast-enhanced ultrasound for preoperative T-staging of gastric cancer: a meta-analysis

**DOI:** 10.1186/s40644-025-00861-5

**Published:** 2025-04-03

**Authors:** MingYue Lv, Xu Hui, Xin Yang, SuSu Li, ZhiGuo Mao, XinHua Zhang, KeHu Yang

**Affiliations:** 1 The First Clinical Medical College of Gansu University of Traditional Chinese Medicine, Lanzhou, 730000 China; 2 Evidence-Based Medicine Centre, School of Basic Medical Science, Lanzhou University, Lanzhou, 730000 China; 3 Centre for Evidence-Based Social Science/Center for Health Technology Assessment, School of Public Health, Lanzhou University, Lanzhou, 730000 China; 4 Gansu Key Laboratory of Evidence-Based Medicine, Lanzhou University, Lanzhou, 730000 China; 5 Department of Ultrasound Medicine, Gansu University of Chinese Medicine, Gansu Provincial People’S Hospital, No. 204, Donggang West Road, Chengguan District, Lanzhou, 730000 China

**Keywords:** Gastric cancer, Computer tomography, Double contrast-enhanced ultrasound, Tumor staging, GRADE

## Abstract

**Background:**

Accurate preoperative staging of gastric cancer (GC) depends on effective diagnostic methods. Enhanced computed tomography (enhanced-CT) is a widely used and reliable preoperative assessment tool for GC, Double Contrast-Enhanced Ultrasound (DCEUS) can display the structure and layers of the gastric wall more accurately, and has high sensitivity (SE) and specificity (SP).

**Objective:**

The present study aims to conduct a comprehensive meta-analysis comparing the preoperative T-staging accuracy of DCEUS and enhanced-CT.

**Methods:**

A systematic literature search was conducted across PubMed, Embase, Web of Science, and Cochrane Library to identify eligible articles from inception to February 19, 2024. The study included both prospective and retrospective studies involving patients with GC who underwent DCEUS or enhanced-CT. This encompassed studies utilizing comparative diagnostic test accuracy (CDTA) with both DCEUS and enhanced-CT, as well as studies employing single diagnostic test accuracy (SDTA) with either DCEUS or enhanced-CT alone. Risk of bias was assessed using the Quality Assessment Of Diagnostic Accuracy Studies-C (QUADAS-C) and Assessment Of Diagnostic Accuracy Studies-2 (QUADAS-2) tool. The quality of evidence for each outcome was assessed using GRADE (Grading of Recommendations Assessment, Development and Evaluation).

**Results:**

A total of 39 studies involving 6,374 patients were included in this meta-analysis. Among these, 3 studies (319 patients) directly compared dynamic contrast-enhanced ultrasound (DCEUS) and enhanced computed tomography (CT), while 31 studies (5,180 patients) evaluated enhanced CT alone, and 5 studies (875 patients) assessed DCEUS alone.

For the direct comparison studies (CDTA), DCEUS demonstrated higher sensitivity (SE) and specificity (SP) for T1-T4 staging compared to enhanced CT, with moderate to low certainty of evidence. Specifically, DCEUS showed superior performance in detecting early-stage (T1) and advanced-stage (T4) tumors. Enhanced CT, while effective, had lower sensitivity across all stages, particularly for T1 tumors.

In the single-modality studies (SDTA), DCEUS consistently showed higher sensitivity for T2-T4 staging compared to enhanced CT, with comparable specificity. However, the certainty of evidence for indirect comparisons was very low, highlighting the need for further high-quality comparative studies.

Overall, DCEUS appears to be a promising modality for gastric cancer T staging, particularly for early-stage detection, but the limited number of direct comparative studies underscores the need for more robust evidence.

**Conclusion:**

Current evidence indicates that DCEUS significantly outperforms enhanced-CT in terms of SE and diagnostic accuracy for preoperative T-staging of GC, while maintaining comparable SP. However, these findings require further validation through rigorous studies with larger sample sizes and improved methodological quality.

**Supplementary Information:**

The online version contains supplementary material available at 10.1186/s40644-025-00861-5.

## Introduction

Gastric cancer (GC) occupies a prominent position globally, ranking as the fifth most prevalent malignancy and the third leading cause of cancer-related deaths, posing a significant health burden worldwide [1].Within gastric cancer management protocols, preoperative T-staging constitutes a fundamental component of tumor staging systems [2], This diagnostic modality provides critical guidance for both disease severity stratification and therapeutic regimen selection [3–5]. Therefore, the utilization of appropriate diagnostic imaging tools, precise clinical staging, and optimized surgical techniques is imperative for enhancing prognostic outcomes [1, 6]. A precise preoperative T-staging is indispensable for devising personalized treatment strategies, anticipating patient outcomes, guiding adjuvant therapy, and evaluating treatment efficacy. These aspects collectively contribute to improving the overall survival rates among GC patients, thus advancing the standard of care in this challenging disease [7–10].

In the management of GC, obtaining precise T-staging information through imaging modalities is of utmost importance. Currently, endoscopic ultrasonography (EUS), enhanced computed tomography (CT), magnetic resonance imaging (MRI), and dual contrast-enhanced ultrasonography (DCEUS) constitute the primary methods employed for clinical T-staging [11–14]. Given the advancements and widespread availability of CT equipment, enhanced CT has emerged as a noninvasive and frequently utilized tool for preoperative staging of GC [15–17]. As a noninvasive examination technique, contrast-enhanced CT has inherent limitations, particularly in terms of resolving soft tissues, which can sometimes hinder the accurate distinction of gastric wall layers. Additionally, the requirement of iodine contrast agent injection poses challenges, especially for patients with renal metabolic disorders. Furthermore, concerns persist regarding the potential adverse effects of ionizing radiation associated with this modality [18]. Therefore, it is crucial to meticulously assess the risks and implement necessary precautions during its application to safeguard patient safety and maintain the accuracy of the examination.

As a novel non-invasive diagnostic modality, DCEUS represents a significant advancement in medical imaging, combining the advantages of gastric filling contrast-enhanced ultrasound with intravenous contrast-enhanced ultrasound techniques [19–21]. Utilizing venous acoustic contrast agents as dynamic tracers within the vasculature, DCEUS effectively discriminates between benign and malignant lesions based on their distinct blood supply patterns and surrounding tissue infiltration characteristics [22]. In recent years, DCEUS has garnered widespread clinical application in Asia [23], particularly in the evaluation of GC. Its utility extends to microvascular perfusion imaging across various organs, enabling the assessment of lesion invasion depth and angiogenesis through the analysis of contrast agent enhancement duration and perfusion patterns within the local tissue. DCEUS further provides insights into the heterogeneous histological features of vascularization [24–27], thereby facilitating the differential diagnosis of benign and malignant gastric ulcers and enhancing the detection rate of malignant lesions [28]. Consequently, DCEUS has emerged as a promising technology actively promoted and widely utilized in GC screening and precise preoperative staging [15, 29]. However, it's worth noting that due to the interference of gas, ultrasound examination has limitations in assessing the depth of tumor invasion, especially in detecting early GC, and its diagnostic accuracy may be compromised [21]. And the interpretation and accuracy of DCEUS findings remain partially dependent on the operator's experience and skill, necessitating continued education and standardization efforts to optimize its diagnostic performance [30]. During the past few years, scholars have undertaken extensive investigations into DCEUS technology. Wang et al. [31] conducted a study encompassing 206 GC patients, exploring the clinical utility of combining DCEUS with dynamic contrast-enhanced multi-slice CT (MSCT) for preoperative T staging of GC. The correct diagnosis rate of DCEUS was 90.91% in T1 staging, 88.89% in T2 staging, 78.95% in T3 staging, 82.86% in T4 staging, and 83.98% in summation. Additionally, Xu et al. [32] analyzed six studies involving 926 patients with GC, the pooled SE and SP of DCEUS were 0.67 and 0.98 for T1 stage, 0.81 and 0.95 for T2 stage, 0.89 and 0.86 for T3 stage and 0.87 and 0.96 for T4 stage, respectively. Furthermore, Zhang et al. [33] reported significant superiority to CT in T1 (RR=1.57, 95% CI=1.20–2.05, *p*=0.001) and T2 (RR=1.41, 95% CI=1.16–1.71, *p*=0.001), demonstrating that DCEUS serves as a viable complementary diagnostic tool alongside gastroscopy for clinical T staging of GC. However, the widespread acceptance of DCEUS for effective T staging of GC remains limited. In this article, we aim to quantitatively assess the diagnostic role of DCEUS in the staging of primary GC. Specifically, our study focuses on conducting a meta-analysis to compare the accuracy of DCEUS with enhanced-CT in the preoperative staging of GC.

Comparative diagnostic test accuracy (CDTA) allows for the rigorous comparison of the accuracy of two tests, employing a study design that involves administering both tests to the same population and subsequently comparing their outcomes to a uniform reference standard, thereby facilitating a direct comparison. However, it is noteworthy that in numerous instances, primary research has primarily focused on evaluating the accuracy of individual diagnostic tests, termed single diagnostic test accuracy (SDTA), such as DCEUS or enhanced-CT, against a reference standard within distinct populations and standalone studies [34]. In such scenarios, the comparison of the pertinent tests becomes indirect, as no direct head-to-head evaluation exists. This indirect comparison introduces additional complexities and limitations, which are beyond the purview of this paper. Consequently, our meta-analysis, which aims to address questions related to comparative test accuracy, comprehensively includes both studies utilizing CDTA methodologies and those relying on indirect comparisons across separate SDTA studies. By synthesizing this comprehensive evidence base, we strive to provide a rigorous and informed analysis of the relative diagnostic accuracies of the tests under investigation.

## Materials and methods

This study strictly adhered to the guidelines outlined in the Preferred Reporting Items for Systematic Reviews and Meta-Analyses of Diagnostic Test Accuracy (PRISMA-DTA) statement [35], and it was prospectively registered in the International Prospective Register of Systematic Reviews with the unique registration number(PROSPERO, CRD42023489558) [36].

### Literature search

The PubMed, Embase, Cochrane Library, and Web of Science databases were systematically searched to retrieve relevant articles published from their inception until December 29, 2023, without any language or publication type restrictions. The search strategy included topic terms and text words, specifically encompassing the following keywords: "Stomach Neoplasms", "Tomography", "X-Ray Computed", "double contrast enhanced ultrasonography", "sensitivity”, “specificity" and "Predictive Value of Tests". This comprehensive search strategy allowed for the retrieval of a wide range of studies pertaining to the diagnostic accuracy and predictive capabilities of various imaging techniques in the detection and assessment of stomach neoplasms. The detailed search strategy employed in this study is provided in the Supplementary Material Table S1.

### Inclusion and exclusion criteria and study selection

The studies were selected based on the following stringent criteria: (a) Participant characteristics: patients who underwent preoperative endoscopic biopsy or had a pathologically confirmed diagnosis of GC, underwent gastrectomy for GC, and had no prior history of chemotherapy, radiotherapy, targeted therapy, immunotherapy, or any other cancer-related treatments; (b) Index tests: preoperative staging of GC conducted using DCEUS examination or enhanced-CT examination; (c) Reference standards: diagnosis of GC established by postoperative histopathology; (d) Outcome measures: evaluation of the accuracy of screening tools for assessing the T-staging; (e) Study designs: inclusion of both prospective and retrospective studies, encompassing both studies using CDTA with both DCEUS and enhanced-CT and studies using SDTA with either DCEUS or enhanced-CT alone.

The T stage classification, based on the depth of invasion of the primary tumor, was precisely defined as follows: T1 stage denoted tumor invasion limited to the lamina propria mucosa and submucosa; T2 stage indicated tumor infiltration into the muscularis propria; T3 stage implied tumor infiltration into the subserosal connective tissue without invasion of the surrounding peritoneum or adjacent structures; and T4 stage encompassed tumors invading the serosa (T4a) or invading adjacent tissues or organs (T4b).

Studies were excluded based on the following exclusion criteria: (a) Cases involving non-operable resectable lesions with metastases detected during preoperative evaluation; (b) Patients who were medically contraindicated for surgical intervention; (c) Publications in the form of letters to the editor, case reports, editorials, review articles, syntheses, or meta-analyses; (d) Studies that did not utilize the standardized TNM classification system for GC stagin.

The studies were systematically imported into the reference management software, EndNote 20, and the online systematic review platform, Rayyan (https://www.rayyan.ai/), for rigorous screening. Two independent reviewers (MYL, XY) carefully evaluated the literature, adhering strictly to the pre-established inclusion and exclusion criteria. Any disparities encountered during the screening process were resolved through thorough discussion or negotiation with the third reviewer (SSL) until a consensus was achieved.

### Data extraction

Data extraction from eligible studies was performed independently by two reviewers (LMY and YX), utilizing a pre-established data extraction form. In the event of any discrepancies, a third reviewer (XJB) was consulted for resolution. The extracted information encompassed several categories:(a) Study characteristics, including the first author's name, the study's design, the country of origin, and the year of publication.(b) Patient clinical characteristics, which encompassed the total number of patients, their age, sex, histopathological grading criteria, and the type of diagnostic modality employed. (c) Diagnostic performance metrics, ensuring the availability of sufficient data to construct a 2 x 2 contingency table. This table encompassed the predictive value of the studies, accuracy, sensitivity (SE), and specificity (SP), as well as the counts of true positives (TP), true negatives (TN), false positives (FP), and false negatives (FN). Additionally, positive likelihood ratios (PLR), negative likelihood ratios (NLR), diagnostic odds ratios (DOR), receiver operating characteristic (ROC) curves, and the area under the curve (AUC) were also extracted. For the calculation of the 2x2 contingency tables and the associated statistical metrics, the statistical software STATA version 16 (Stata Corp LP, College Station, USA) was utilized.

### Risk of bias assessment

Risk of bias of included studies was assessed using the Quality Assessment Of Diagnostic Accuracy Studies-C (QUADAS-C) [37]and Assessment Of Diagnostic Accuracy Studies-2 (QUADAS-2) tool [38]. The results were independently assessed and cross-checked by two investigators (MYL and YX), and any disagreement was achieved consensus for each item under the help of the third author (SSL).

The QUADAS-2 test consists of four key areas: patient selection, index testing, reference standards, and flow and timing. QUADAS-C is an extension of QUADAS-2 designed to assess the risk of bias in studies of comparative accuracy. QUADAS-C retained the same 4-domain structure as QUADAS-2 (patient selection, index testing, reference standards, and flow and time) and included additional questions for each QUADAS-2 domain.

### Grading of evidence

The quality of evidence for each outcome was assessed using GRADE (Grading of Recommendations Assessment, Development and Evaluation). The initial GRADE certainty of the body of CDTA evidence starts at high, regardless of the study design and subsequently adjusted based on five factors that could decrease confidence: risk of bias, indirectness, inconsistency, imprecision, and publication bias. Additionally, three factors were considered to potentially increase confidence: dose-effect relationship, significant trial effect size, and residual negative bias. The overall strength of evidence was then categorized as having high, medium, low or very low confidence levels [39].

GRADE Guidance 31 recommend that raters assess the certainty of evidence in comparative (CDTA) studies and between-study (SDTA) comparisons separately. Second, if comparative studies constitute high certainty evidence, there is typically no need to look for between-study comparisons. Third, if the evidence from comparative studies is of moderate certainty or lower, suggest assessing the certainty of between-study comparisons and to choose the highest certainty evidence to inform recommendations (between-study comparisons to provide moderate certainty evidence at best due to issues regarding indirectness) [34]. The detailed certainty of evidence employed in this study was provided in the Supplementary Material.

### Publication bias

When more than 10 studies were included, deek’s funnel plot asymmetry test was used to detect publication bias, with *P*<0.05 indicating publication bias.

### Statistical analysis

The statistical analysis in this study was conducted using three software packages: RevMan 5.3 from The Cochrane Collaboration, Meta-Disc software version 1.4 [40] and the STATA version 16 (Stata Corp LP, CollegeStation, USA). RevMan 5.3 was used to plot histograms to assess risk of bias. RevMan 5.3 and Meta-Disc 1.4 and STATA 16 software were used to analyze the SE, SP, PLR, NLR, DOR, AUC, 95% confidence interval (CI) and summary receiver operating characteristic (SROC) curve of DCEUS and enhanced-CT among CDTA and SDTA using bivariate random-effects model. We used Meta-Disc 1.4 software to summary estimates when there were less than three studies. This approach allowed us to calculate summary estimates while dealing with potential sources of variation caused by: (1) imprecision of estimates within individual studies; (2) correlation across studies; and (3) variation between studies. enhanced-CT and DCEUS, which ROC curve position is closer to the upper left corner, suggests that it has better diagnostic efficacy.

This study conducted a meta-analysis on continuous variables, and the weighted mean difference (WMD) was selected as the combined effect size to reflect the difference between the DCEUS and the enhanced-CT. Mean difference (MD) of SE was calculated as SE DCEUS minus SE enhanced-CT, and MD of SP was calculated as SP DCEUS minus SP enhanced-CT. The Q test and I^2^ statistic were used to assess the heterogeneity of the included studies. Given possible heterogeneity across studies, we used a random-effects model to integrate effect sizes across studies. Using this model, we obtained a more robust and accurate estimate of the overall effect size, with corresponding CIs and hypothesis testing results. Gradepro (Guideline Development Tool, https://gdt.gradepro.org/app/) was used to calculate the actual clinical effect, the corresponding number of patients detected in each 1,000 patients with GC in diagnostic accuracy between DCEUS and enhanced-CT according to the incidence of four stages of T1-T4.

## Results

### Search results

**Fig. 1:**
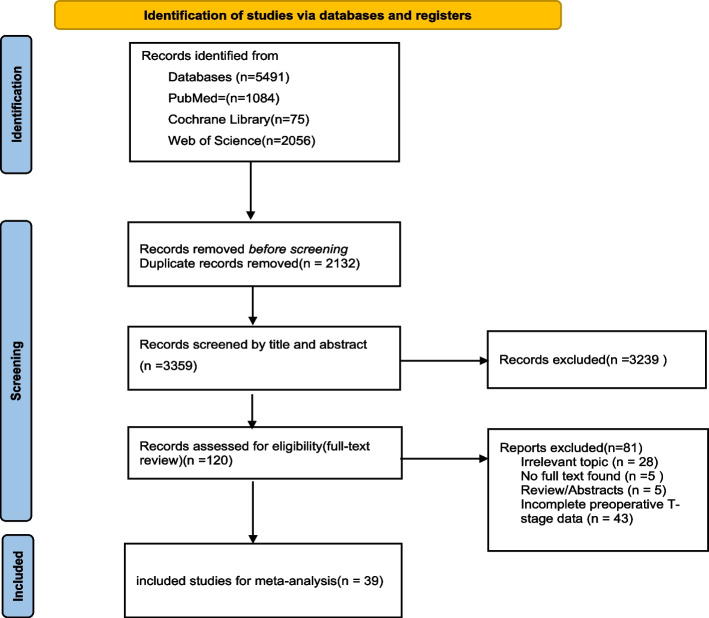
PRISMA flow chart of the literature retrieval

A rigorous literature search yielded a total of 5,491 articles. Upon diligent elimination of duplicate studies, the corpus was narrowed down to 3,359 articles. Following a meticulous screening of titles and abstracts, 3,239 articles were excluded based on their relevance to the research objectives. Subsequently, we evaluated the eligibility of 120 studies, resulting in the exclusion of an additional 81 articles. Finally, a meticulous selection process led to the inclusion of 39 articles that fully met the pre-defined criteria. The PRISMA flow diagram provides a systematic and transparent overview of the entire study selection process (Figure 1).

### Characteristics of the included studies

The study design and patient characteristics of the 39 included studies, 6,374 cases are shown in Table S2 and Table S3. Women accounted for about one-third, age ranges from 17 to 87 years old, with the majority of middle-aged and elderly people over 50 years old, 11 were prospective [28, 41–50] and 28 were retrospective [31, 51–77]. Thirty-three studies were based on Asian populations [28, 31, 47, 49, 56, 57, 59, 61, 64, 65, 69, 71, 75, 77, 78], 1 study on an African population [44] and 5 studies on European populations [48, 62, 66, 67, 73]. Thirteen studies used AJCC (3th, 5th, 7th, 8th) (American Joint Committee on Cancer) TNM staging. Nine studies used UICC (4th, 5th, 6th, 8th) (Union for International Cancer Control) TNM staging. The 7th edition of AJCC TNM staging was the most common staging criteria in 8 studies. Three studies were CDTA trials [31, 61, 75] with both DCEUS and enhanced-CT, with a study population of 319. Thirty-one studies were DTA trials with enhanced-CT only [41, 42, 46, 48, 51, 52, 55, 67, 68, 70, 72, 73, 76, 77], with 5,180 participants. Five studies were DTA trials with DCEUS only [45, 49, 60, 64, 65], with a study population of 875.

## Quality assessment

**Fig. 2:**
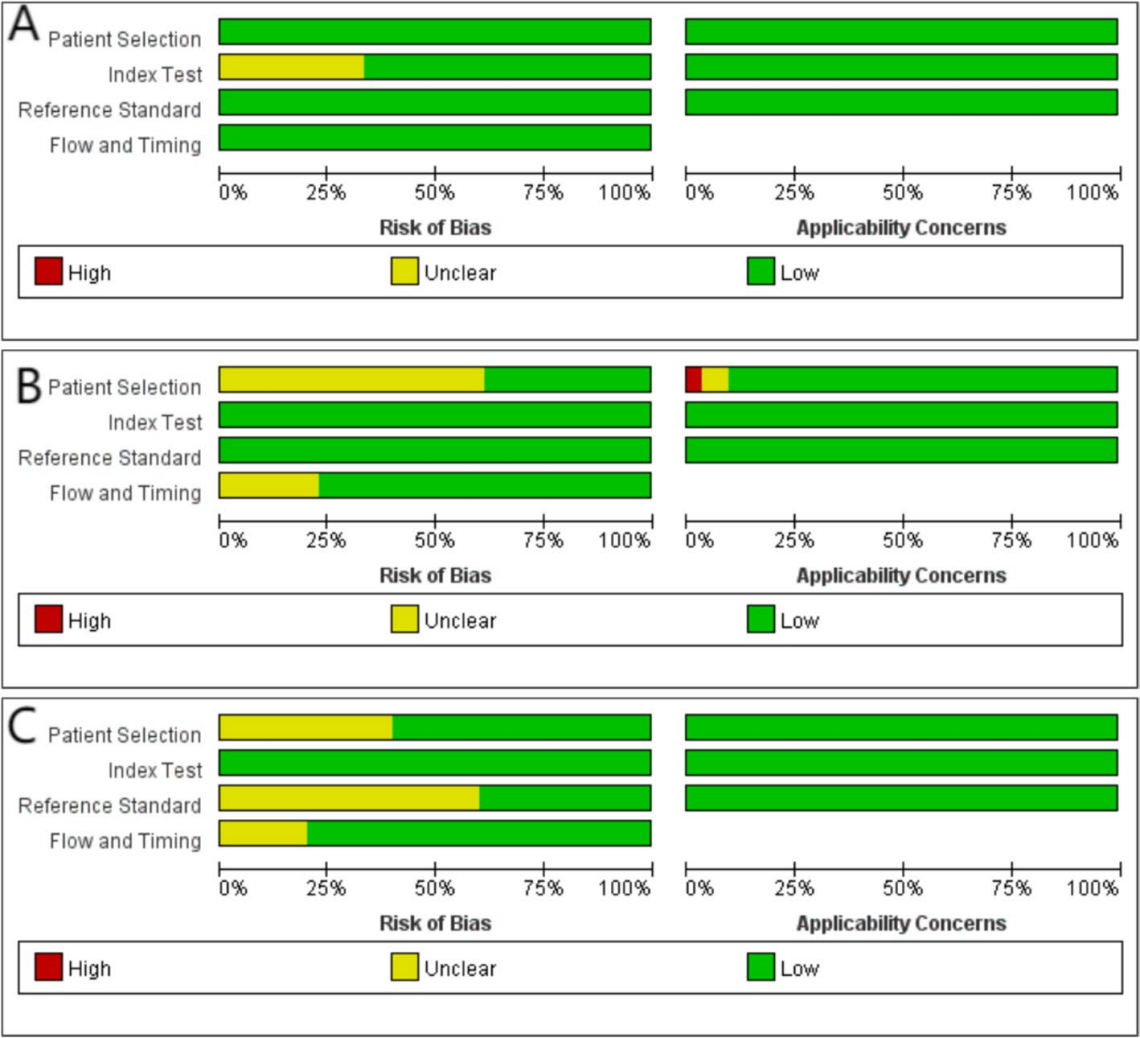
QUADAS. **A** The quality of 3 CDTA studies (Evaluated by QUADAS-C). **B** The quality of 31 SDTA studies with enhanced CT, summarises the risk of bias and applicability issues for the studies (Evaluated by QUADAS-2). **C** The quality of 5 SDTA studies with DCEUS, summarises the risk of bias and applicability issues for the studies (Evaluated by QUADAS-2). Detailed results are displayed in Figure S2

Quality of the included CDTA was assessed by the QUADAS-C. In terms of patient selection, no risk of bias was identified. In index test, 2 studies were low risk of bias, 1 studies was unclear risk. No risk of bias was identified in the reference standard domain. In the bias of flow and timing no risk of bias was identified. All studies had a low risk of bias for clinical applicability (Fig. 2A, Figure S2A).

Quality of the included SDTA was assessed by the QUADAS-2. In 31 SDTA trials with enhanced-CT, in terms of patient selection, 12 (39%) studies was low risk of bias, 19 (61%) studies was unclear. No risk of bias was identified in the index test domain. For the reference standard, no risk of bias was identified. In the bias of flow and timing 7 (23%) studies were considered to be at unclear risk of bias and 24 (77%) at low risk of bias. In terms of applicability, 1 (3%) studies had high risk of bias and 2 (6%) studies had unclear bias applicability in the area of patient selection.

In 5 SDTA trials with DCEUS, in terms of patient selection, 4 (80%) studies was low risk of bias, 1 (20%) studies was unclear risk of bias. No risk of bias was identified in the index test domain. For the reference standard, 2 (40%) studies was low risk of bias, 3 (60%) studies was unclear risk of bias. In the bias of flow and timing 1 (20%) studies were considered to be at unclear risk of bias and 4 (80%) at low risk of bias. All studies had a low risk of bias for clinical applicability. (Fig. 2B, C, Figure S2B, Figure S2C).

### Certainty of evidence

Based on the GRADE assessment, the evidentiary level for direct comparative studies ranged from low to moderate, whereas the evidentiary level for indirect comparative studies was classified as very low. Overall, the pooled evidentiary level fell within the low to moderate category [39, 51].

#### Direct comparative studies (CDTA)

In the GC population, the prevalence rates were 1.4% for T1 stage, 21.8% for T2 stage, 31.7% for T3 stage, and 42.4% for T4 stage. Compared with enhanced-CT, DCEUS could diagnose 6 (3 to 8) more GC patients with T1 stage; 51 (11 to 90) more patients with T2 stage; 35 more (16 fewer-86 more) patients with T3 stage; 60 (16 to 103) more patients with T4 stage per 1000 cases. DCEUS could reduce misdiagnosis 0 (24 fewer-24 more) GC patients with T1 stage; 8 fewer (29 fewer-13 more) patients with T2 stage; 89 more (40–137 more) patients with T3 stage; 11 more (12 fewer-34 more) patients with T4 stage per 1000 cases. In T1-T4 stage, DCEUS and enhanced-CT have good consistency in TN among preoperative staging of GC. However, DCEUS is slightly superior to enhanced-CT in TP (Table 1).

**Table 1:** GRADE a Systematic Assessment of the Grading of Evidence for T staging among CDTA

T staging	Downgrading the quality of evidence								Summary of results			
	Outcome	No of studies (No of patients)	Study design	Risk of bias	Indirectness	Inconsistency	Imprecision	Publication Bias	Effect per 1,000 patients tested (95%CI)			Certainty of the evidence
									DCEUS	CT	Discrepancy	
T1	TP	3(319)	Completely paired	Serious^a^	Not Serious	Not Serious	Untested	Untested	13 more (11–14 more)	7 more (5-10more)	6 more (3–8 more)	⊕ ⊕ ⊕ ○ Moderate
	FN								1 more (0–3 more)	7 more (4–9 more)	6 fewer (8–3 fewer)	
	TN	3(319)	Completely paired	Serious^a^	Not Serious	Not Serious	Untested	Untested	976 more (956–986 more)	976 more (947–986 more)	0 (24 fewer-24 more)	⊕ ⊕ ⊕ ○ Moderate
	FP								10 more (0–30 more)	10 more (0–39 more)	0 (24 fewer-24 more)	
T2	TP	3(319)	Completely paired	Serious^a^	Not Serious	Not Serious	Untested	Untested	177 more (146–196 more)	126 more (94–155 more)	51 more (11–90 more)	⊕ ⊕ ⊕ ○ Moderate
	FN								41 fewer (22–72 fewer)	92 fewer (63–124 fewer)	51 fewer (90–11 fewer)	
	TN	3(319)	Completely paired	Serious^a^	Not Serious	Not Serious	Untested	Untested	766 more (743–774 more)	774 more (751–782 more)	8 fewer (29 fewer-13 more)	⊕ ⊕ ⊕ ○ Moderate
	FP								16 more (8–39 more)	8 more (0–31 more)	8 more (13 fewer-29 more)	
T3	TP	3(319)	Completely paired	Serious^a^	Not Serious	Not Serious	Untested	Untested	260 more (222–288 more)	225 more (184–263 more)	35 more (16 fewer-86 more)	⊕ ⊕ ⊕ ○ Moderate
	FN								57 more (29–95 more)	92 more (54–133 more)	35 fewer (86 fewer-16 more)	
	TN	3(319)	Completely paired	Serious^a^	Not Serious	Serious^b^	Untested	Untested	567 more (533–594 more)	478 more (437–512 more)	89 more (40–137 more)	⊕ ⊕ ○○ Low
	FP								116 more (89–150 more)	205 more (171–246 more)	89 fewer (137–40 fewer)	
T4	TP	3(319)	Completely paired	Serious^a^	Not Serious	Serious^b^	Untested	Untested	331 more (297–356 more)	271 more (242–305 more)	60 more (16–103 more)	⊕ ⊕ ○○ Low
	FN								93 more (68–127 more)	153 more (119–182 more)	60 fewer (103–16 fewer)	
	TN	3(319)	Completely paired	Serious^a^	Not Serious	Not Serious	Untested	Untested	547 more (518–564 more)	536 more (501–513 more)	11 more (12 fewer-34 more)	⊕ ⊕ ⊕ ○ Moderate
	FP								29 more (12-58more)	40 more (63–75 more)	11 fewer (34 fewer-12 more)	

A fully contextualized threshold was used for rating certainty of evidence

Among the gastric cancer population, T1 stage accounted for 1.4%, T2 stage accounted for 21.8%, T3 stage accounted for 31.7%, and T4 stage accounted for 42.4%

*TP* True Positive, *FN* False Negative, *TN* True Negative, *FP* False Positive, *CDTA* comparative diagnostic test accuracy studies, *SDTA* Single diagnostic test accuracy studies

^a^a unclear risk of bias in the domain of "Index Test", but the results of the SE analyses showed that there was not much effect on accuracy. Therefore, On this basis, it is judged that there is no serious risk of bias in the assessment of the system and the risk of bias in this area is downgraded by only 1 level

^b^Unexplained heterogeneity in effects ranging from one side of the threshold to the other

### Risk of bias

Across three studies, all participants underwent both DCEUS and enhanced-CT imaging procedures. One study exhibited an unclear risk of bias in the "Index Test" domain. Consequently, it is concluded that there is no significant risk of bias in the overall assessment of the system, and the risk of bias in this specific area is only downgraded by a single level.

### Indirectness

The included articles showed that the population of interest of studies and systematic reviews was basically the same, which was all patients with GC confirmed by pathology, and all patients received DCEUS and enhanced-CT before surgery. DCEUS and enhanced-CT were performed in accordance with clinical practice, and the target disease of all studies was GC. The indirect aspect is not downgraded after comprehensive judgment.

### Inconsistency

Forest plots of SP for T3 stage and SE for T4 stage showed low overlap of 95% CI for MD and unexplained heterogeneity from one side of the threshold to the other. The difference in overall assessment SE was thus reduced by one grade in terms of inconsistency. The de-escalation factors for the differences in SP were consistent with the differences in SE.

### Imprecision

We used noncontextualized certainty ratings to make choices of ranges without value judgments that do not involve modeling based on relevant criteria in the GRADE Evidence to Decision framework for tests. The way of setting ranges for SE and SP is using existing limits of the 95% CIs, which implies precision is not routinely part of the rating.

### Publication bias

Publication bias was not tested because there were only 3 CDTA studies.

#### Between-study (indirect) comparisons

### Risk of bias

Across the five DCEUS studies, two studies exhibited unclear risk of bias in the Patient Selection domain, three studies displayed unclear risk of bias in the Reference Standard domain, and one study demonstrated unclear risk of bias in the Flow and Timing domain. Consequently, the pooled risk of bias across these three domains remains unclear risk of bias.

Within the 31 enhanced-CT studies, 19 studies had an unclear risk of bias in the Patient Selection domain, while two studies exhibited unclear risk of bias in the Reference Standard domain. Additionally, seven studies showed an unclear risk of bias in the Flow and Timing domain. Given these findings, the overall risk of bias across these domains remains unclear risk of bias.

For the purposes of comparison, we adopted the highest risk of bias judgment for each domain, resulting in an unclear risk of bias for the Reference Standard and Patient Selection domains, as well as an unclear risk of bias for the remaining two domains. Based on this assessment, the overall risk of bias warranted a downgrading of two levels.

### Indirectness

Because of an indirect comparison, the tests were evaluated in different studies with very different characteristics, there was already very serious indirectness that would warrant rating down by two levels in absence of further indirectness concerns.

### Inconsistency

For the results TP and FN, we evaluated the inconsistency of TP and FN separately, because the SE of the two tests ranged from about 50% to more than 90%, and we thought that it might be inconsistent to compare them. Our scores for TP and FN were reduced by one level. For outcomes TN and FP (SP), we also observed heterogeneity between the two tests. Therefore, we decided to downgrade the inconsistency rating by one level.

### Imprecision

We used noncontextualized certainty ratings to make choices of ranges without value judgments that do not involve modeling based on relevant criteria in the GRADE Evidence to Decision framework for tests. Therefore, both outcomes were not rated down for imprecision.

### Publication bias

This systematic review employed a comprehensive search strategy across four databases and searched for both unpublished and ongoing studies. Deek's funnel plot test showed that the publication bias of T1 (*P* = 0.26), T3 (*P* = 0.51) and T4 (*P* = 0.24) was not significant. However, T2 (*P* = 0.01) had the risk of publication bias. Overall, we had serious concerns about publication bias at T2 stage (overall judgment: serious publication bias). We did not encounter any serious concerns about publication bias in T1, T3, and T4 stage (Figure S21).

### Meta-analysis for T-staging

The overall sensitivity (SE), specificity (SP), diagnostic odds ratio (DOR), positive likelihood ratio (PLR), and negative likelihood ratio (NLR) of DCEUS and enhanced-CT for gastric cancer (GC) patients are summarized in Tables 2, 3, 4 and 5.

**Table 2:** SE and SP for DCEUS and enhanced-CT imaging to diagnose T staging

Characteristics	Sensitivity(%)				Specificity(%)			
	DCEUS (95%CI)	Enahnced-CT (95%CI)	Mean Difference (95%CI)	P-Value of Mean Difference	DCEUS (95%CI)	Enahnced-CT (95%CI)	Mean Difference (95%CI)	P-Value of Mean Difference
CDTA								
T1	91(76, 98)	53(35, 70)	38 (10, 66)	0.007	99(97, 100)	99(96, 100)	0(−3, 2)	0.80
T2	81(67, 90)	58(43, 71)	18 (−8, 44)	0.18	98(95, 99)	94(90, 96)	3 (−3, 9)	0.30
T3	82(70, 91)	71(58, 83)	9 (−17, 35)	0.49	83(78, 87)	70(64, 75)	−1 (−31, 28)	0.94
T4	78(70, 84)	64(57, 72)	−15 (−64, 33)	0.53	95(90, 98)	93(87, 89)	1 (−7, 8)	0.89
STDA								
T1	62(51, 73)	56(45, 66)	/	/	97(95, 99)	98(96, 99)	/	/
T2	81(76, 86)	63(54, 70)	/	/	95(89, 98)	88(85, 91)	/	/
T3	89(85, 92)	77(72, 82)	/	/	89(84, 93)	84(81, 87)	/	/
T4	90(83, 94)	77(70, 83)	/	/	96(94, 97)	96(94, 97)	/	/

*CT* computed tomography, *DCEUS* double contrast-enhanced ultrasound, *CDTA* comparative diagnostic test accuracy studies, *SDTA* Single diagnostic test accuracy studies

**Table 3:** DOR and PLR for NLR and enhanced-CT imaging to diagnose T staging

Characteristics	DCEUS			Enhanced-CT		
	DOR(95%CI)	PLR(95%CI)	NLR(95%CI)	DOR(95%CI)	PLR(95%CI)	NLR(95%CI)
CDTA						
T1	605.65(116.35–3152.74)	62.22(23.27–166.40)	0.12(0.050.30)	62.94(17.23–229.87)	23.39(8.46–64.66)	0.49(0.26–0.94)
T2	96.11(5.96–1549.65)	23.06(3.28–162.16)	0.26(0.07–1.00)	22.08(8.38–58.20)	8.20(2.16–25.79)	0.45(0.33–0.62)
T3	15.46(7.06–32.87)	3.63(1.95–6.75)	0.26(0.07–1.00)	11.09(0.91–135.22)	3.44(0.99–11.87)	0.32(0.07–1.48)
T4	48.81(20.64–115.45)	12.95(6.21–27.02)	0.36(0.16–0.78)	34.33(1.70–692.14)	9.97(1.34–73.86)	0.33(0.11–0.99)
SDTA						
T1	51.33(22.52–116.98)	19.87(10.03–39.35)	0.39(0.29–0.52)	58.05(31.63–106.55)	26.23(14.85–46.35)	0.45(0.36–0.57)
T2	86.74(29.88–251.82)	17.19(6.79–43.79)	0.20(0.15–0.26)	12.70(8.89–18.15)	5.35(4.32–6.64)	0.42(0.34–0.52)
T3	67.15(40.62–111.01)	8.36(5.57–12.55)	0.12(0.09–0.17)	18.37(12.97–26.01)	4.92(4.04–5.99)	0.27(0.22–0.33)
T4	213.43(95.47–477.12)	22.50(14.57–34.75)	0.11(0.06–0.19)	79.20(49.23–127.40)	19.06(13.57–26.76)	0.24(0.18–0.32)

*CDTA* comparative diagnostic test accuracy studies, *SDTA* Single diagnostic test accuracy studies, *DOR* Diagnostic Odds Ratio, *PLR* Positive Likelihood Ratio, *NLR* Negative Likelihood Ratio

**Table 4:** GRADE a Systematic Assessment of the Grading of Evidence for T staging among SDTA

T staging	Downgrading the quality of evidence								Summary of results		
	Outcome	NO.of studies	Study design	Risk of bias	Indirectness	Inconsistency	Imprecision	Publication	Effect per 1,000 tested (95%CI)		Certainty of evidence
									DCEUS	CT	
T1	TP	31(5432)	SDTA	Very serious^a^	very serious^b^	serious^c^	Untested	Not serious	9 more (7–10 more)	8 more (6–9 more)	○○○○
											very low
	FN								5 more (4–7 more)	6 more (5–8 more)	
	TN	31(5432)	SDTA	Very serious^a^	very serious	serious	Untested	Not serious	956 more (937–976 more)	966 more (947–976 more)	○○○○
											very low
	FP								30 more (10–49 more)	20 more (10–39 more)	
T2	TP	36(6055)	SDTA	Very serious^a^	very serious	serious	Untested	serious^d^	177 more (166–187 more)	137 more (118–153 more)	○○○○
											very low
	FN								41 more (31–52 more)	81 more (65–100 more)	
	TN	36(6055)	SDTA	Very serious^a^	very serious	serious	Untested	serious	743 more (696–766 more)	688 more (665–712 more)	○○○○
											very low
	FP								39 more (16–86 more)	94 more (70–117 more)	
T3	TP	36(6055)	SDTA	Very serious^a^	very serious	serious	Untested	Not serious	282 more (269–292 more)	244 more (228–260 more)	○○○○
											very low
	FN								35 more (25–48 more)	73 more (57–89 more)	
	TN	36(6055)	SDTA	Very serious^a^	very serious	serious	Untested	Not serious	608 more (574–635 more)	574 more (553–594 more)	○○○○
											very low
	FP								75 more (48–109 more)	109 more (89–130 more)	
T4	TP	35(6011)	SDTA	Very serious^a^	very serious	serious	Untested	Not serious	382 more (352–399 more)	326 more (297–352 more)	○○○○
											very low
	FN								42 more (25–72 more)	98 more (72–127 more)	
	TN	35(6011)	SDTA	Very serious^a^	very serious	serious	Untested	Not serious	553 more (541–559 more)	553 more (541–559 more)	○○○○
											very low
	FP								23 more (17–35 more)	23 more (17–35 more)	

TP: true positives; FN: false negatives; TN: true negatives; FP: false positives; CDTA, comparative diagnostic test accuracy studies; SDTA,Single diagnostic test accuracy studies

^a^Unclear risk of bias for the Reference Standard and Patient Selection domains, as well as an unclear risk of bias for the remaining two domains.indirect comparison and doubts about directness for both the intervention and comparison tests

^b^Due to an indirect comparison, tests in different studies with highly diverse characteristics were evaluated. Therewas already significant indirectness, Indirectness ratings were downgraded by one level

^c^Due to significant sensitivity variations between TP and FN, alongside heterogeneity in TN and FP , directcomparisons lacked reliability, inconsistency ratings were downgraded by one level

^d^Publication bias was detected

**Table 5:** Overall confidence of evidence

T staging	Outcome	Single test accuracy studies	Completely paired	Confidence of the pooled evidence
T1	TP and FN	○○○○ very low	⊕ ⊕ ⊕ ○ Moderate	⊕ ⊕ ⊕ ○ Moderate
	TN and FP	○○○○ very low	⊕ ⊕ ⊕ ○ Moderate	⊕ ⊕ ⊕ ○ Moderate
T2	TP and FN	○○○○ very low	⊕ ⊕ ⊕ ○ Moderate	⊕ ⊕ ⊕ ○ Moderate
	TN and FP	○○○○ very low	⊕ ⊕ ⊕ ○ Moderate	⊕ ⊕ ⊕ ○ Moderate
T3	TP and FN	○○○○ very low	⊕ ⊕ ⊕ ○ Moderate	⊕ ⊕ ⊕ ○ Moderate
	TN and FP	○○○○ very low	⊕ ⊕ ⊕ ○ Low	⊕ ⊕ ⊕ ○ Low
T4	TP and FN	○○○○ very low	⊕ ⊕ ⊕ ○ Low	⊕ ⊕ ⊕ ○ Low
	TN and FP	○○○○ very low	⊕ ⊕ ⊕ ○ Moderate	⊕ ⊕ ⊕ ○ Moderate

If comparative studies constitute high certainty evidence, there is typically no need to look for between-study comparisons. If the evidence from comparative studies is of moderate certainty or lower, suggest assessing the certainty of between-study comparisons and to choose the highest certainty evidence to inform recommendations

*TP* true positives, *FN* false negatives, *TN* true negatives, *FP* false positives

#### T1 staging

A total of 34 studies involving 5,751 patients were included for T1 staging analysis. Among these, 3 studies were direct comparison trials (CDTA) [31, 61, 75], 3 were single-modality trials (SDTA) with DCEUS alone [45, 64, 65],, and 28 were SDTA trials with enhanced-CT alone [28, 43, 44, 47, 56, 57, 59, 63, 66, 69, 71, 74, 77, 78].

### Direct comparative studies

**Fig. 3:**
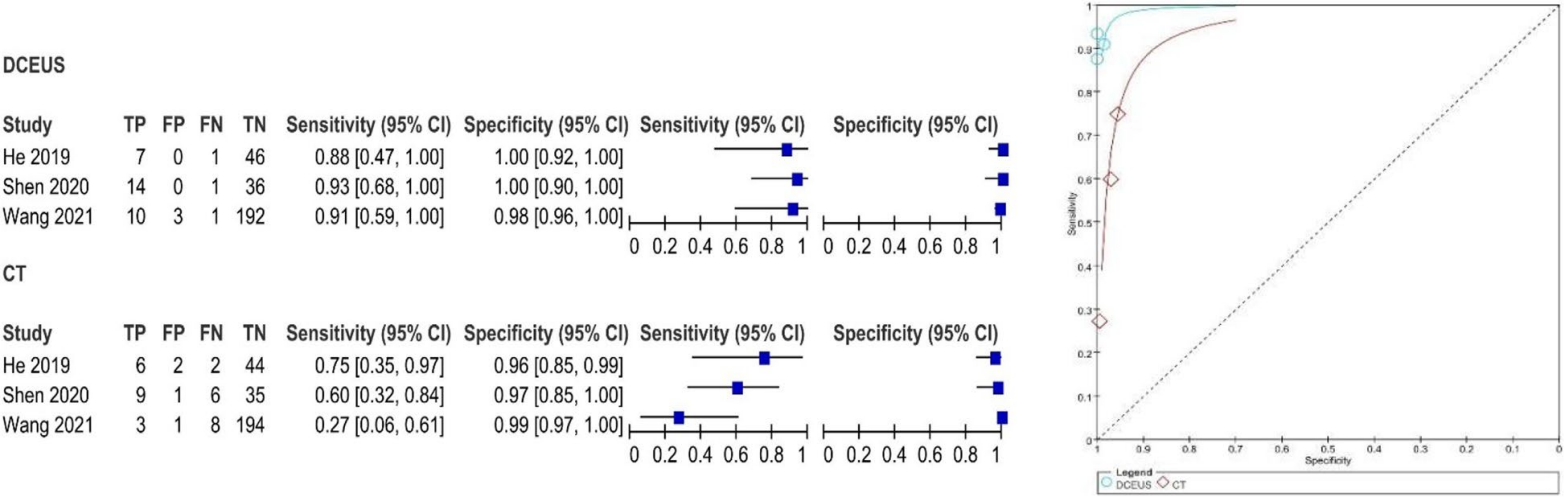
Pooled SE and SP in T1 staging of DCEUS and enhanced-CT. SE: sensitivity; SP: specificity; CT, computed tomography; DCEUS, double contrast-enhanced ultrasound

The pooled SE and SP of DCEUS were 0.91 (95% CI: 0.76, 0.98; I2 = 0.0%, *p* = 0.90) and 0.99 (95% CI: 0.97, 1.00; I2 = 5.7%, *p* = 0.35), respectively, with moderate certainty of evidence. The DOR, PLR, and NLR were 605.65 (116.35, 316.74), 62.22 (23.27, 166.40), and 0.12 (0.15, 0.30), respectively (Figure S4). For enhanced-CT, the pooled SE and SP were 0.53 (95% CI: 0.35, 0.70; I2 = 59.5%, *p* = 0.08) and 0.99 (95% CI: 0.96, 1.00; I2 = 46.1%, *p* = 40.16), with moderate certainty of evidence. The DOR, PLR, and NLR were 62.94 (17.23, 229.87), 23.39 (8.46, 64.66), and 0.49 (0.26, 0.94), respectively (Figure S4). The AUC for DCEUS (0.99) was higher than that for enhanced-CT (0.94) (Fig. 3, Figure S3). Compared with enhanced-CT, the mean differences (MD) for SE and SP of DCEUS were 0.38 (95% CI: 0.10, 0.66; I2 = 47%, *p* = 0.15) and 0 (95% CI: −0.03, 0.02; I2 = 0%, *p* = 0.38), respectively (Figure S5).

### Between-study (indirect) comparisons

**Fig. 4:**
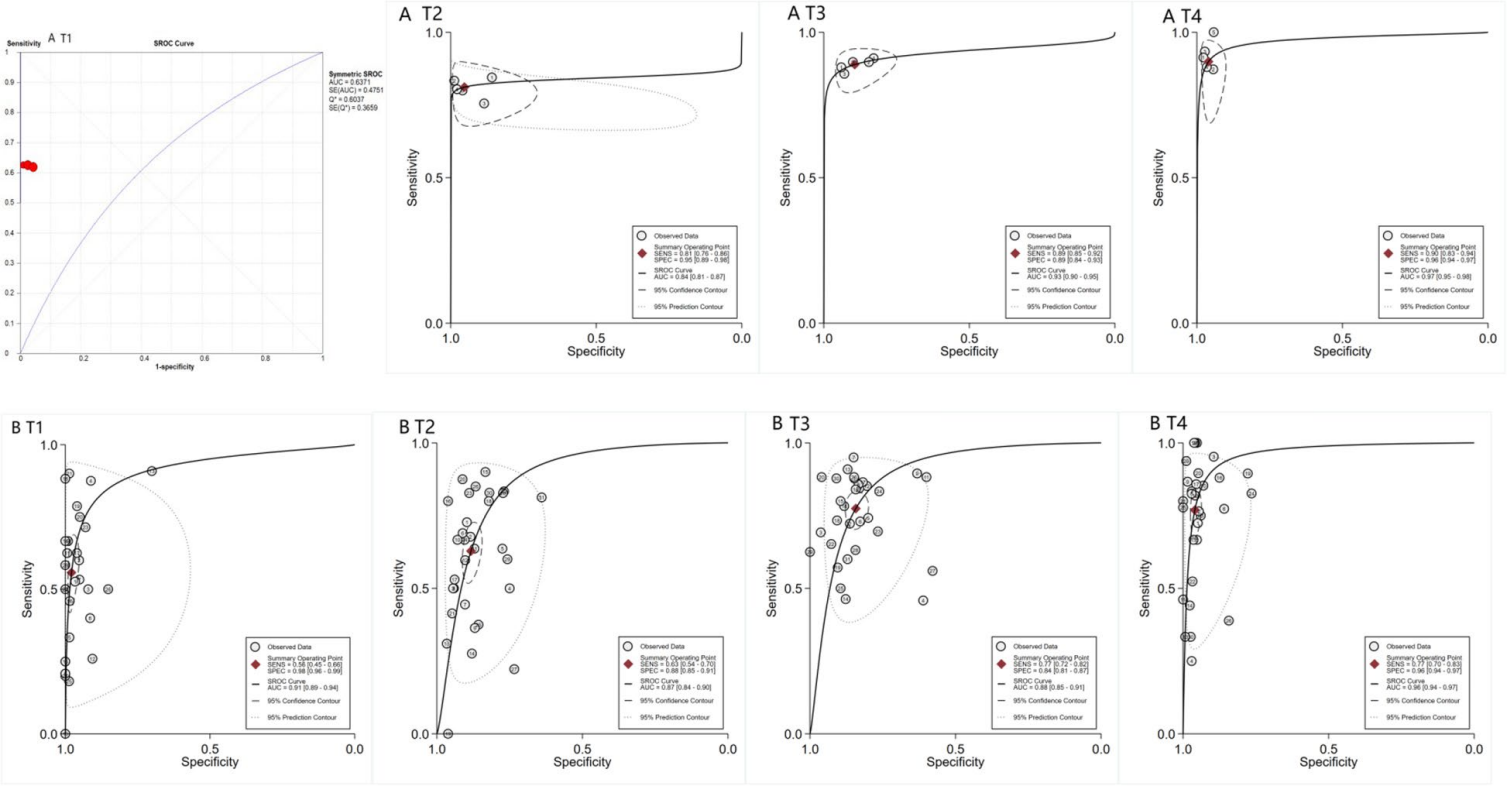
SROC curves for all studies at each stage of T1, T2, T3, T4. 1. **A** DCEUS for T1, T2, T3, T4 staging of gastric cancer; (**B**) enhanced-CT for T1, T2, T3, T4 staging of gastric cancer; CT, computed tomography; DCEUS, double contrast enhanced ultrasound. SROC, summary receiver operating characteristic

The pooled SE and SP of DCEUS were 0.62 (95% CI: 0.51, 0.73; I^2^ = 0.00%, *p* = 0.99) and 0.97 (95% CI: 0.95, 0.99; I^2^ = 49.50%, *p* = 0.14), respectively, with very low certainty of evidence. The DOR, PLR, and NLR were 51.33 (22.52, 116.98), 19.87 (10.03, 39.35), and 0.39 (0.29, 0.52), respectively (Figure S7, Figure S8). For enhanced-CT, the pooled SE and SP were 0.56 (95% CI: 0.45, 0.66; I^2^ = 94.55%, *p* < 0.001) and 0.98 (95% CI: 0.96, 0.99; I^2^ = 96.03%, *p* < 0.001), with very low certainty of evidence. The DOR, PLR, and NLR were 58.05 (31.63, 106.55), 26.23 (14.85, 46.35), and 0.45 (0.36, 0.57), respectively (Figure S7, Figure S8). The AUC for DCEUS (0.64) was lower than that for enhanced-CT (0.91) (Fig. 4).

#### T2 staging

Thirty-nine studies reporting data on 6,374 patients were included in T2 staging of GC, three studies were CDTA trial [31, 61, 75], five were SDTA trials with DCEUS alone [45, 49, 60, 64, 65], and thirty-one were SDTA trials with enhanced-CT alone [28, 43, 44, 47, 56, 57, 59, 62, 66, 69, 71, 74, 77, 78].

### Direct comparative studies

**Fig. 5:**
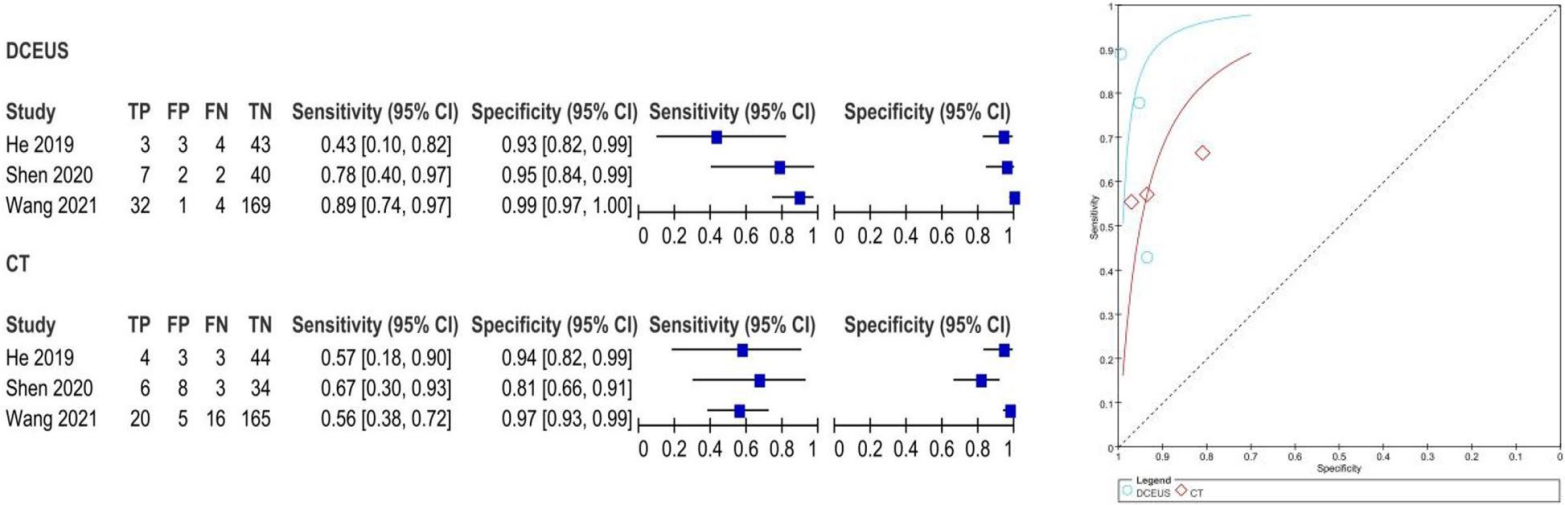
Pooled SE and SP in T2 staging of DCEUS and enhanced-CT. SE: sensitivity; SP: specificity; CT, computed tomography; DCEUS, double contrast-enhanced ultrasound

The pooled SE and SP of DCEUS were 0.81 (95% CI: 0.67, 0.90; I^2^ = 70.2%, *p* = 0.04) and 0.98 (95% CI: 0.95, 0.99; I^2^ = 69.1%, *p* = 0.04), respectively, with moderate certainty of evidence. The DOR, PLR, and NLR were 96.11 (5.96, 1,549.65), 23.06 (3.28, 162.16), and 0.26 (0.07, 1.00), respectively (Figure S10). For enhanced-CT, the pooled SE and SP were 0.58 (95% CI: 0.43, 0.71; I^2^ = 0.0%, *p* = 0.83) and 0.94 (95% CI: 0.90, 0.96; I^2^ = 83%, *p* < 0.01), with moderate certainty of evidence. The DOR, PLR, and NLR were 22.08 (8.38, 58.20), 8.20 (2.61, 25.79), and 0.45 (0.33, 0.62), respectively (Figure S10). The AUC for DCEUS (0.88) was higher than that for enhanced-CT (0.75) (Fig. 5, Figure S9). Compared with enhanced-CT, the MD for SE and SP of DCEUS were 0.18 (95% CI: −0.08, 0.44; I^2^ = 38%, *p* = 0.20) and 0.03 (95% CI: −0.03, 0.09; I^2^ = 29%, *p* = 0.25), respectively (Figure S10).

### Between-study (indirect) comparisons

The pooled SE and SP of DCEUS were 0.81 (95% CI: 0.76, 0.86; I^2^ = 0.00%, *p* = 0.79) and 0.95 (95% CI: 0.89, 0.98; I^2^ = 87.67%, *p* < 0.001), respectively, with very low certainty of evidence. The DOR, PLR, and NLR were 86.74 (29.88, 251.82), 17.19 (6.79, 43.79), and 0.20 (0.15, 0.26), respectively (Figure S7, Figure S8). For enhanced-CT, the pooled SE and SP were 0.63 (95% CI: 0.54, 0.70; I^2^ = 84.52%, *p* < 0.001) and 0.88 (95% CI: 0.85, 0.91; I^2^ = 81.64%, *p* < 0.001), with very low certainty of evidence. The DOR, PLR, and NLR were 12.70 (8.89, 18.15), 5.35 (4.32, 6.64), and 0.42 (0.34, 0.52), respectively (Figure S7, Figure S8). The AUC for DCEUS (0.84) was slightly lower than that for enhanced-CT (0.87) (Fig. 4).

#### T3 staging

Thirty-nine studies reporting data on 6,374 patients were included in T3 staging, three studies were CDTA trials [31, 61, 75], five were SDTA trials with DCEUS alone [45, 49, 60, 64, 65], and thirty-one were SDTA trials with enhanced-CT alone [28, 43, 44, 47, 56, 57, 59, 62, 66, 69, 71, 74, 77, 78].

### Direct comparative studies

**Fig. 6:**
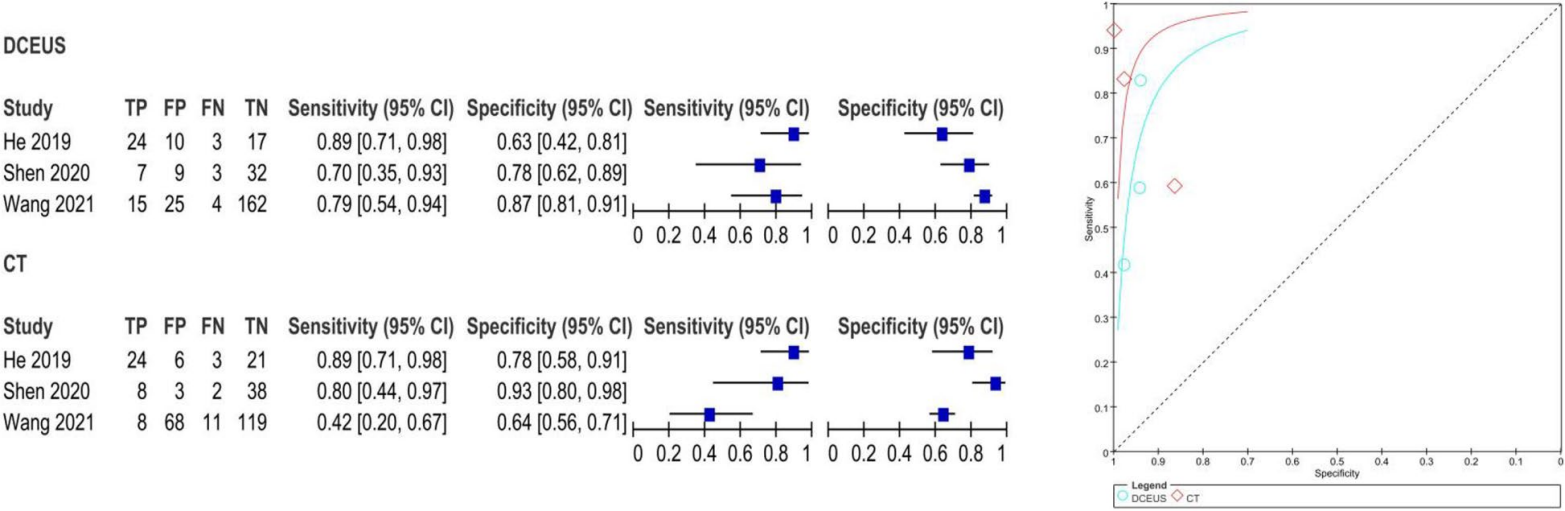
Pooled SE and SP in T3 staging of DCEUS and enhanced-CT. SE: sensitivity; SP: specificity; CT, computed tomography; DCEUS, double contrast-enhanced ultrasound

The pooled SE and SP of DCEUS were 0.82 (95% CI: 0.70, 0.91; I^2^ = 0.0%, *p* = 0.38) and 0.83 (95% CI: 0.78, 0.87; I^2^ = 77.0%, *p* = 0.013), respectively, with moderate to low certainty of evidence. The DOR, PLR, and NLR were 15.46 (7.06, 33.87), 3.63 (1.95, 6.75), and 0.26 (0.15, 0.46), respectively (Figure S14). For enhanced-CT, the pooled SE and SP were 0.71 (95% CI: 0.58, 0.83; I^2^ = 83.7%, *p* < 0.001) and 0.70 (95% CI: 0.64, 0.75; I^2^ = 83.3%, *p* < 0.01), with moderate to low certainty of evidence. The DOR, PLR, and NLR were 11.09 (0.91, 135.22), 3.44 (0.99, 11.87), and 0.32 (0.07, 1.48), respectively (Figure S14). The AUC for DCEUS (0.87) was slightly higher than that for enhanced-CT (0.86) (Fig. 6, Figure S13). Compared with enhanced-CT, the MD for SE and SP of DCEUS were 0.09 (95% CI: −0.17, 0.35; I^2^ = 59%, *p* = 0.09) and −0.01 (95% CI: −0.31, 0.28; I^2^ = 90%, *p* < 0.001), respectively (Figure S15).

### Between-study (indirect) comparisons

The pooled SE and SP of DCEUS were 0.89 (95% CI: 0.85, 0.92; I^2^ = 0.0%, *p* = 0.89) and 0.89 (95% CI: 0.84, 0.93; I^2^ = 63.95%, *p* = 0.03), respectively, with very low certainty of evidence. The DOR, PLR, and NLR were 67.15 (40.62, 111.01), 8.36 (5.57, 12.55), and 0.12 (0.09, 0.17), respectively (Figure S7, Figure S8). For enhanced-CT, the pooled SE and SP were 0.77 (95% CI: 0.72, 0.82; I^2^ = 86.31%, *p* < 0.001) and 0.84 (95% CI: 0.81, 0.87; I^2^ = 75.32%, *p* < 0.001), with very low certainty of evidence. The DOR, PLR, and NLR were 18.37 (12.97, 26.01), 4.92 (4.04, 5.99), and 0.27 (0.22, 0.33), respectively (Figure S7, Figure S8). The AUC for DCEUS (0.93) was higher than that for enhanced-CT (0.88) (Fig. 4).

#### T4 staging

Thirty-eight studies reporting data on 6,330 patients were included in T4 staging of GC, three studies were CDTA trials [31, 61, 75], five were SDTA trials with DCEUS alone [45, 49, 60, 64, 65], and thirty were SDTA trials with enhanced-CT alone [41, 42, 46, 48, 52, 55, 58, 67, 68, 70, 72, 73, 76, 77].

### Direct comparative studies

**Fig. 7:**
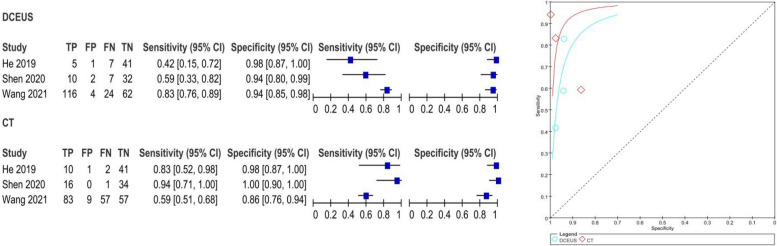
Pooled SE and SP in T4 staging of DCEUS and enhanced-CT. SE: sensitivity; SP: specificity; CT, computed tomography; DCEUS, double contrast-enhanced ultrasound

The pooled SE and SP of DCEUS were 0.78 (95% CI: 0.70, 0.84; I^2^ = 84%, *p* < 0.01) and 0.95 (95% CI: 0.90, 0.98; I^2^ = 0.0%, *p* = 0.62), respectively, with low to moderate certainty of evidence. The DOR, PLR, and NLR were 48.81 (20.64, 115.45), 12.95 (6.21, 27.02), and 0.36 (0.16, 0.78), respectively (Figure S18). For enhanced-CT, the pooled SE and SP were 0.64 (95% CI: 0.57, 0.72; I^2^ = 83.6%, *p* < 0.01) and 0.93 (95% CI: 0.87, 0.89; I^2^ = 80.6%, *p* = 0.01), with low to moderate certainty of evidence. The DOR, PLR, and NLR were 34.33 (1.70, 692.14), 9.97 (1.34, 73.86), and 0.33 (0.11, 0.99), respectively (Figure S18). The AUC for DCEUS (0.96) was higher than that for enhanced-CT (0.87) (Fig. 7, Figure S17). Compared with enhanced-CT, the MD for SE and SP of DCEUS were −0.15 (95% CI: −0.64, 0.33; I^2^ = 91%, *p* < 0.001) and 0.01 (95% CI: −0.07, 0.08; I^2^ = 37%, *p* = 0.21), respectively (Figure S19).

### Between-study (indirect) comparisons

The pooled SE and SP of DCEUS were 0.90 (95% CI: 0.83, 0.94; I2 = 0.00%, *p* = 0.83) and 0.96 (95% CI: 0.94, 0.97; I2 = 6.76%, *p* = 0.37), respectively, with very low certainty of evidence. The DOR, PLR, and NLR were 213.43 (95.47, 477.12), 22.50 (14.57, 34.75), and 0.11 (0.06, 0.19), respectively (Figure S7, Figure S8). For enhanced-CT, the pooled SE and SP were 0.77 (95% CI: 0.70, 0.83; I2 = 77.81%, *p* < 0.001) and 0.96 (95% CI: 0.94, 0.97; I2 = 74.87%, *p* < 0.001), with very low certainty of evidence. The DOR, PLR, and NLR were 79.20 (49.23, 127.40), 19.06 (13.57, 26.76), and 0.24 (0.18, 0.32), respectively (Figure S7, Figure S8). The AUC for DCEUS (0.97) was slightly higher than that for enhanced-CT (0.96) (Fig. 4).

## Discussion

We conducted a diagnostic meta-analysis of dynamic contrast-enhanced ultrasound (DCEUS) and enhanced computed tomography (CT) for T-staging assessment in gastric cancer (GC), encompassing 39 studies with a total of 6,374 patients. Our analysis focused on three high-quality studies using direct comparison (CDTA) and 36 studies using indirect comparison (SDTA) to ensure the reliability of our findings.

Our findings align with and expand on prior meta-analyses of gastric cancer T-staging imaging modalities. DCEUS has higher sensitivity than enhanced-CT for early T1–T2 stages, but there are discrepancies in T3–T4 staging accuracy. In T1, DCEUS's pooled sensitivity (91%) beats enhanced-CT's (53%, *p* = 0.007), matching Zhang et al. and Xu et al. [32, 33]. For T2, DCEUS (81%) also outperforms CT (58%), consistent with Zhang et al., likely due to better layer visualization [33]. In T3, DCEUS has moderate sensitivity (82%) but lower specificity (83%) than Xu et al.'s 86%, due to T3/T4 distinction challenges [32]. In T4, both DCEUS (78%) and CT (64%) have reduced sensitivity, as noted by Nie et al. [79], highlighting the need for multimodal approaches. DCEUS's T1 sensitivity (91%) is below EUS's (82–94%). Zhang et al. confirmed EUS's higher T1 accuracy [33].

The overall quality of evidence, combining direct and indirect comparisons, ranged from low to moderate. Direct evidence was graded as moderate to low due to risks of bias and inconsistency, primarily caused by unexplained heterogeneity among studies. Indirect evidence was generally of lower quality, particularly at the T2 stage, due to issues such as risk of bias, inconsistency, indirectness, and publication bias.

While our assessment relied primarily on direct comparison evidence, supplemented by indirect data, the methodological limitations of both approaches highlight the need for further refinement. Additionally, differences in sample characteristics between direct and indirect studies, as well as potential biases from variations in examination instruments and staging standards over the past decade, pose challenges to our findings. Future research should address these limitations to improve the accuracy and reliability of GC staging assessments.

Accurate T-staging is critical for tailoring treatment strategies to individual patient conditions, optimizing outcomes, and improving quality of life. For early-stage GC (T1-T2), surgical intervention is relatively straightforward, often without the need for adjuvant chemotherapy. In contrast, advanced-stage GC (T3-T4) requires more complex surgical approaches and is often followed by adjuvant therapies. Endoscopic resection, compared to traditional surgery, offers advantages such as reduced trauma, fewer complications, faster recovery, and lower costs, with a 5-year survival rate exceeding 90% [80], These benefits underscore the importance of accurate staging in guiding effective treatment strategies for early GC [1].

Enhanced CT provides high-resolution images and multi-planar reconstruction capabilities, allowing visualization of tumor enhancement patterns and differentiation between neoplastic and healthy tissues [81]. However, it has limitations in assessing tumor infiltration depth [82], particularly in early-stage GC, and struggles to differentiate between T2-T3 and T3-T4 stages [83], Tyhese limitations highlight the need for complementary diagnostic modalities in clinical practice [84]. DCEUS, combining ultrasound with oral and intravenous contrast agents [11], offers superior effectiveness and reliability in preoperative T-staging compared to enhanced CT.

The superior sensitivity of DCEUS for T1-2 staging may be attributed to three key factors [21, 32, 85–89]: (1) Real-time visualization of gastric wall layers (mucosa/submucosa) through high-frequency ultrasound probes. (2) Dynamic perfusion imaging of microbubble contrast agents enabling detection of microvascular changes in early tumors; [3] Absence of ionizing radiation artifacts that may obscure subtle mucosal lesions on CT.

It provides high-contrast, dynamic visualization of tumor perfusion and excellent image quality [21]. While both DCEUS and multi-slice CT (MSCT) can predict the cT stage of GC, our findings confirm that DCEUS achieves a significantly higher diagnostic accuracy (83.98%) compared to MSCT (55.34%) [31]. DCEUS is an economical, safe, and highly acceptable diagnostic modality with significant potential for GC staging. It provides a theoretical basis for precise staging and supports the rational selection of treatment plans [85]. Despite its promise, the widespread adoption of DCEUS is limited by the need for advanced operator skills and a lack of supporting evidence. If scaled up, DCEUS could revolutionize preoperative GC staging in developing countries, offering a novel approach that requires further promotion and clinical validation [33].

### Clinical integration of DCEUS: challenges and opportunities

The integration of DCEUS into routine work flows requires addressing three key factors: Cost-effectiveness analyses suggest that while DCEUS contrast agents (e.g., SonoVue® at 80–120 perdose)are cheaper than CT contrast media(80–120 perdose) are cheaper than CT contrast media(150–200 per dose), the upfront cost of high-end ultrasound systems ($120,000–250,000) may limit accessibility in low-resource settings [90–92]. However, the absence of radiation exposure and reusability of ultrasound hardware could yield long-term savings. Second, although ultrasound is widely available globally, DCEUS expertise remains concentrated in tertiary centers. Telemedicine platforms enabling real-time remote supervision by expert radiologists may democratize access [93].

### Subgroup analysis for heterogeneity

Subgroup analyses were systematically performed to investigate potential sources of heterogeneity (I^2^ > 50%) in the diagnostic accuracy across T1-T4 gastric cancer staging. Due to the small number of studies included in the direct comparison and the indirect comparison of DCEUS, only a further subgroup analysis was conducted on the indirect comparison of enhanced CT to explore the possible sources of heterogeneity. This analysis was stratified by four critical dimensions: geographic region, CT technological parameters, sample size thresholds, and study design methodology (Table 6). Initial stratification by geographic region revealed distinct regional patterns, particularly in the early tumor stages. Asian populations demonstrated significantly higher sensitivity for T1 staging (58%, 95% CI: 47–68%) compared to European/African cohorts (44%, 15–79%), despite comparable high specificity across regions (98% vs. 97%). This disparity may be related to the predominance of intestinal-type gastric carcinomas in Asia, which typically exhibit well-defined morphological features that are conducive to sonographic detection, in contrast to the higher prevalence of infiltrative diffuse-type malignancies in Western populations, which complicate staging precision. For T3 staging, Asian studies maintained diagnostic superiority in both sensitivity (79% vs. 69%) and specificity (84% vs. 81%), whereas T4 staging showed geographic parity in sensitivity (77% vs. 78%) and specificity (96% vs. 95%).

**Table 6:** Subgroup analysis of heterogeneity sources in gastric cancer staging diagnostic accuracy

Analysis	No. of models	Pooled SE(95% CI)	I^2^(%)	P1	Pooled SP(95% CI)	I^2^(%)	P2	Pooled PLR(95% CI)	I^2^(%)	P3	Pooled NLR(95% CI)	I^2^(%)	P4	AUC
T1														
Geographical Regions														
Asian	24	0.58 (0.47–0.68)	95.42	0	0.98 (0.96–0.99)	96.56	0	27.86 (14.67–52.93)	94.44	0	0.43 (0.34–0.55)	97.21	0	0.91
European and African	4	0.44 (0.15–0.79)	76.09	0.01	0.97 (0.90–0.99)	72.81	0.01	17.48(5.77–52.92)	0	0.71	0.57(0.3–1.1)	58.79	0.06	0.93
CTs with varying slice numbers														
≥ 64	9	0.56 (0.39–0.71)	89.68	0	0.97 (0.94–0.98)	71.85	0	17.87 (8.05–39.66)	65.8	0	0.46 (0.31–0.67)	93.28	0	0.94
< 64	19	0.56 (0.44–0.68)	94.22	0	0.98 (0.96–0.99)	96.93	0	32.75 (16.00–67.04)	94.79	0	0.45 (0.34–0.58)	96.86	0	0.91
Sample size														
≥ 100	12	0.52(0.36–0.67)	97.58	0	0.98 (0.95–0.99)	98	0	25.65(11.26–58.45)	96.29	0	0.49(0.36–0.67)	98.43	0	0.9
< 100	16	0.61(0.48–0.73)	47.28	0.02	0.98 (0.95–0.99)	59.97	0	25.42(11.47–56.35)	2.22	0.06	0.40(0.28–0.56)	70.01	0	0.93
Types of Study Design														
prospective	10	0.53(0.42–0.63)	48.25	0.04	0.98(0.95–0.99)	80.29	0	35.14(12.91–95.66)	41.15	0	0.48(0.38–0.61)	54.89	0.02	0.83
retrospective	18	0.57(0.43–0.70)	97.1	0	0.97 (0.95–0.99)	96.19	0	21.58(11.09–41.97)	93.91	0	0.44(0.32–0.61)	98.5	0	0.92
T2														
Geographical Regions														
Asian	25	0.62 (0.53–0.70)	85.46	0	0.90(0.87–0.92)	80.17	0	5.95(4.77–7.43)	72.61	0	0.43 (0.34–0.54)	89.41	0	0.88
European and African	6	0.66 (0.44–0.83)	69.27	0.01	0.80(0.74–0.85)	39.1	0.15	3.28(2.04–5.28)	0	0.15	0.42 (0.22–0.80)	86.4	0	0.81
CTs with varying slice numbers														
≥ 64	9	0.65(0.50–0.77)	71.61	0	0.92(0.88–0.94)	69.98	0	7.68(5.61–10.51)	0	0.58	0.39(0.37–0.56)	73.82	0	0.9
< 64	22	0.62 (0.52–0.72)	87.05	0	0.87(0.83–0.89)	82.98	0	4.66(3.66–5.91)	69.57	0	0.43 (0.34–0.56)	89.59	0	0.86
Sample size														
≥ 100	12	0.54(0.43–0.64)	89.41	0	0.91(0.89–0.93)	84.16	0	6.17(4.62–8.22)	80.76	0	0.51(0.40–0.64)	92.47	0	0.89
< 100	19	0.71(0.61–0.79)	52.69	0	0.85(0.80–0.88)	55.82	0	4.66(3.47–6.25)	9.32	0.03	0.35(0.25–0.48)	77.21	0	0.86
Types of Study Design														
prospective	10	0.71(0.61–0.79)	60.9	0.01	0.87(0.82–0.91)	80.85	0	5.55(4.18–7.37)	41.94	0	0.33(0.25–0.44)	42.93	0.07	0.87
retrospective	21	0.58 (0.47–0.68)	85.25	0	0.89(0.85–0.92)	84.28	0	5.20(3.87–6.99)	69.34	0	0.47(0.37–0.61)	87.77	0	0.86
T3														
Geographical Regions														
Asian	25	0.79 (0.73–0.84)	87.43	0	0.84(0.87–0.81)	73.88	0	5.03(4.26–5.95)	58.26	0	0.25(0.20–0.31)	89.45	0	0.89
European and African	6	0.69 (0.56–0.80)	72.39	0	0.81(0.67–0.90)	80.6	0	3.66(1.73–7.74)	77.04	0	0.38(0.23–0.63)	82.33	0	0.81
CTs with varying slice numbers														
≥ 64	9	0.79 (0.70–0.86)	66.47	0	0.85(0.81–0.89)	46.53	0.06	5.35(3.94–7.28)	43.76	0	0.25(0.17–0.37)	73.72	0	0.89
< 64	22	0.77 (0.70–0.82)	89.2	0	0.83(0.79–0.87)	79.55	0	4.66(3.7–5.86)	67.91	0	0.28(0.21–0.36)	90.32	0	0.88
Sample size														
≥ 100	12	0.82(0.75–0.87)	93.59	0	0.83(0.80–0.86)	83.12	0	4.86(4.06–5.82)	73.1	0	0.22(0.16–0.30)	94.95	0	0.89
< 100	19	0.72 (0.65–0.78)	52.02	0	0.86(0.80–0.89)	70.26	0	5.07(3.47–7.40)	62.12	0	0.32(0.25–0.41)	63.14	0	0.85
Types of Study Design														
prospective	10	0.82(0.77–0.87)	52.07	0.03	0.87(0.79–0.92)	63.88	0	6.14(4.03–9.35)	25.41	0.01	0.20(0.16–0.26)	53.99	0.02	0.9
retrospective	21	0.76(0.69–0.82)	88.42	0	0.84(0.80–0.87)	79.65	0	4.67(3.68–5.93)	73.88	0	0.29(0.22–0.38)	88.95	0	0.87
T4														
Geographical Regions														
Asian	24	0.77(0.71–0.83)	66.9	0	0.96(0.95–0.97)	74.52	0	20.32(14.37–28.73)	52.07	0	0.24(0.18–0.31)	77.44	0	0.95
European and African	6	0.78(0.44–0.94)	83.86	0	0.95(0.89–0.98)	81.45	0	15.32(5.91–39.72)	67.75	0	0.24(0.07–0.75)	91.17	0	0.96
CTs with varying slice numbers														
≥ 64	8	0.73(0.55–0.86)	74.76	0	0.95(0.89–0.97)	74.74	0	13.39(7.50–23.91)	23.36	0.02	0.28(0.16–0.49)	88.17	0	0.94
< 64	22	0.77(0.69–0.74)	74.01	0	0.96(0.95–0.98)	74	0	21.41(14.43–31.78)	55.17	0	0.24(0.17–0.33)	82.74	0	0.97
Sample size														
≥ 100	12	0.80(0.72–0.86)	76.78	0	0.96(0.95–0.98)	80.03	0	22.62(14.94–34.25)	62.33	0	0.21(0.15–0.29)	85.32	0	0.96
< 100	18	0.74(0.61–0.83)	65.6	0	0.95(0.92–0.97)	66.95	0	15.62(9.25–26.39)	40.88	0	0.28(0.18–0.43)	78.17	0	0.95
Types of Study Design														
prospective	10	0.77(0.64–0.86)	73.93	0	0.96(0.95–0.97)	0	0.74	20.41(14.18–29.37)	80.56	0	0.24(0.15–0.39)	80.56	0	0.97
retrospective	20	0.77(0.68–0.84)	73.2	0	0.96(0.93–0.97)	83.23	0	15.7(11.16–28.29)	70.8	0	0.24(0.17–0.34)	85.17	0	0.95

P1-P4 represent the P values for assessing the overall heterogeneity in each subgroup through the Cochran Q test

*TP* true positives, *FN* false negatives, *TN* true negatives, *FP* false positives, *CT* Computed Tomography, *SROC* summary receiver operating characteristic

Stratification of CT systems by technological parameters (≥ 64-slice vs. < 64-slice) yielded nuanced insights. High-resolution CT demonstrated limited diagnostic advantages for early-stage characterization, with equivalent sensitivity (T1: 56% vs. 56%; T2: 65% vs. 62%) and specificity (T1: 97% vs. 98%; T2: 92% vs. 87%) between the technological tiers, although reduced heterogeneity was observed in T1 staging with advanced systems (I^2^ = 89.68% vs. 94.22%). In the evaluation of advanced stages, modest resolution-dependent improvements were observed for T3 metrics (sensitivity: 79% vs. 77%; specificity: 85% vs. 83%), yet substantial residual heterogeneity persisted (I^2^ > 65%), suggesting inherent limitations of technological solutions in addressing fundamental staging challenges.

Stratification by sample size (*n* = 100 threshold) uncovered critical methodological considerations. Smaller studies (n < 100) exhibited enhanced T1-T2 sensitivity (T1: 61% vs. 52%; T2: 71% vs. 54%) with preserved specificity (T1: 98% vs. 98%; T2: 85% vs. 91%), along with markedly reduced T1 heterogeneity (I^2^ = 47.28% vs. 97.58%), potentially reflecting selection bias towards operable early-stage cases. Conversely, large-scale studies (n ≥ 100) demonstrated improved sensitivity stability in advanced stages (T3: 82% vs. 72%; T4: 80% vs. 74%) but sustained high heterogeneity (I^2^ > 76%), highlighting the complex clinical realities captured in population-level cohorts.

Comparisons of study designs revealed that prospective protocols conferred particular advantages in complex anatomical evaluations. Prospective designs achieved superior T3 staging accuracy (sensitivity: 82% vs. 76%; specificity: 87% vs. 84%) with reduced heterogeneity (I^2^ = 52.07% vs. 88.42%), whereas retrospective studies showed equivalent T4 performance metrics (sensitivity: 77% vs. 77%; specificity: 96% vs. 96%) but elevated heterogeneity levels (I^2^ = 73.2% vs. 73.93%). This dichotomy highlights the critical role of standardized operational protocols in minimizing procedural variability, particularly for staging that requires precise assessment of perivisceral infiltration.

Synthesizing these multidimensional findings, geographic variance mediated by histopathological subtype prevalence and study design rigor emerge as primary modifiers of T1-T3 staging variability, while technological parameters and cohort scale predominantly influence advanced-stage reproducibility. Persistent residual heterogeneity (I^2^ > 50% across subgroups) underscores unresolved confounding factors, including operator-dependent endoscopic technique variance and interinstitutional discrepancies in pathological interpretation criteria, necessitating future standardization efforts to optimize diagnostic consistency in gastric cancer staging.

### Strengths and limitations

This study possesses distinct advantages that contribute to its scientific rigor and comprehensiveness. Firstly, it offers a comprehensive evaluation encompassing all stages of GC, thereby providing a more holistic understanding of diagnostic accuracy of enhanced-CT and DCEUS for preoperative T-staging of GC. Secondly, the credibility of both direct and indirect evidence was rigorously assessed using a graded evidence evaluation system, ensuring the reliability and validity of our findings.

However, it is acknowledged that our study also has certain limitations. Notably, the availability of trials providing direct evidence for CDTA was limited. To mitigate this bias, we incorporated studies on SDTA with DCEUS or enhanced-CT.

Third, the substantial disparity in sample sizes between enhanced-CT (*n* = 31 studies) and DCEUS (*n* = 5 studies) in indirect comparisons may introduce selection bias. We conducted sensitivity analyses excluding low-quality CT studies (The score of QUADAS-2 is ≤ 2 items, which is of low risk), which showed consistent results (Supplementary Fig. 2B). However, this limitation underscores the need for more high-quality DCEUS studies to balance comparative evidence.

Nevertheless, the optimal method for integrating these diverse types of evidence remains a challenge that requires further methodological exploration. Future research is needed to address this issue and enhance the overall quality of evidence synthesis in GC research. [86]. Secondly, the staging criteria and medical equipment utilized in the reviewed literature exhibited inconsistencies, potentially introducing variations in the accuracy of our findings. Lastly, clinical heterogeneity among the included studies, such as inconsistencies in the level of expertise among sonographers, represents an additional source of heterogeneity that may have influenced our results. Given these limitations, further methodological research is warranted to enhance the quality and consistency of evidence synthesis in research.

### Strategies to improve DCEUS reproducibility

To mitigate operator-dependent variability in DCEUS, we propose a three-tiered approach: First, adherence to standardized training programs such as the EFSUMB Level 2 certification [94], which has been shown to reduce diagnostic discrepancies by 25–30% in multicenter trials. Second, strict protocolization of contrast agent administration (e.g., 2.4 mL SonoVue bolus followed by 5 mL saline flush) and imaging acquisition windows (e.g., arterial phase initiated at 20 s post-injection). Third, leveraging emerging technologies such as AI-driven lesion boundary detection algorithms (e.g., U-Net-based systems) to standardize image interpretation. These measures could enhance the clinical adoption of DCEUS for gastric cancer staging [95].

## Conclusion

The results of this meta-analysis firmly establish the feasibility of DCEUS for preoperative tumor staging of GC, providing reliable evidence for its clinical utility. Specifically, DCEUS demonstrates a superior diagnostic accuracy compared to CT in the assessment of T1-T2 GC stages. Furthermore, for T3-T4 staging, DCEUS exhibits comparable diagnostic efficacy to CT. Given these findings, DCEUS is anticipated to emerge as a valuable auxiliary diagnostic tool for clinical T staging of GC. However, it is imperative to note that further comparative diagnostic test accuracy studies are required to comprehensively validate the routine clinical application of DCEUS.

## Supplementary Information

Supplementary Material 1

## Data Availability

No datasets were generated or analysed during the current study.
