# Supplementary material for: Comparison of the diagnostic accuracy of enhanced-CT and double contrast-enhanced ultrasound for preoperative T-staging of gastric cancer: a meta-analysis
Source: Cancer Imaging. 2025 Apr 3;25:48. doi: 10.1186/s40644-025-00861-5 (PMC11966938; doi:10.1186/s40644-025-00861-5)
Supplement: Supplementary file 1 — Supplementary Material 1 [file 40644_2025_861_MOESM1_ESM.pdf]

Content

Grading of evidence 3

Supplementary Table 1. Search Strategy 7

Supplementary Table 2. Characteristics of the included studies 18

Supplementary Table 3. Characteristics of the data included in the study 23

Supplementary Table 4. Specific parameters of CT scan 26

Supplementary Figure 2A. Quality of the 3 included studies (Evaluated by QUADAS-C) 40

Supplementary Figure 2B. Quality of the 31 Single diagnostic test accuracy studies (Evaluated by QUADAS-2) 41

Supplementary Figure 2C. Quality of the 5 Single diagnostic test accuracy studies (Evaluated by QUADAS-2). 42

Supplementary Figure 3. Forest plots of sensitivity, specificity and SROC for DCEUS and enhanced-CT in T1 staging CDTA 43

Supplementary Figure 4. Forest plots of DOR, PLR and NLR for DCEUS and enhanced-CT in T1 staging CDTA 44

Supplementary Figure 5. Forest plots of MD for sensitivity and specificity in T1 staging CDTA 45

Supplementary Figure 6. Forest plots of sensitivity and specificity for DCEUS and enhanced-CT in T1 staging among SDTA 46

Supplementary Figure 7. Likelihood forest plots 47

Supplementary Figure 7. Likelihood forest plots 48

Supplementary Figure 8. Forest plots of DOR for DCEUS and enhanced-CT among SDTA 50

Supplementary Figure 9. Forest plots of sensitivity, specificity and SROC for DCEUS and enhanced-CT in T2 staging CDTA 51

Supplementary Figure 10. Forest plots of DOR, PLR and NLR for DCEUS and enhanced-CT in T2 staging CDTA 53

Supplementary Figure 11. Forest plots of MD for sensitivity and specificity in T2 staging CDTA 54
Supplementary Figure 12. Forest plots of sensitivity and specificity for DCEUS and enhanced-CT in T2 staging among SDTA 55
Supplementary Figure 13. Forest plots of sensitivity, specificity and SROC for DCEUS and enhanced-CT in T3 staging CDTA 56
Supplementary Figure 14. Forest plots of DOR, PLR and NLR for DCEUS and enhanced-CT in T3 staging CDTA 57
Supplementary Figure 15. Forest plots of MD for sensitivity and specificity in T3 staging CDTA 58
Supplementary Figure 16. Forest plots of sensitivity and specificity for DCEUS and enhanced-CT in T3 staging among SDTA 59
Supplementary Figure 17. Forest plots of sensitivity, specificity and SROC for DCEUS and enhanced-CT in T4 staging CDTA 60
Supplementary Figure 18. Forest plots of DOR, PLR and NLR for DCEUS and enhanced-CT in T4 staging CDTA 61
Supplementary Figure 19. Forest plots of MD for sensitivity and specificity in T4 staging CDTA 62
Supplementary Figure 20. Forest plots of sensitivity and specificity for DCEUS and enhanced-CT in T4 staging among SDTA 63
Supplementary Figure 21. The results of Deek's funnel plot tes 64

Grading of evidence

Direct comparative studies (comparative diagnostic test accuracy)

We used noncontextualized certainty ratings to make choices of ranges without value judgments that do not involve modeling based on relevant criteria in the GRADE Evidence to Decision (EtD) framework for tests(1) . The way of setting ranges for SE and SP is using existing limits of the 95% CIs, which implies precision is not routinely part of the rating. What the certainty rating represents certainty that the true difference in accuracy lies within the confidence region of the tests compared or the true difference in SE and SP lies within their respective confidence intervals. It means that the choice of the boundaries for the range of SE and SP does not involve value judgments. That is, the importance of the number of FNs or FPs does not bear on the ranges chosen, and the downstream consequences of the test results have no influence on the certainty ratings of SE and SP. Using this approach, one omits the rating of imprecision, that is, one could have high certainty that the true SE or SP lies within the range set by the CI regardless of whether this range is wide or narrow. The ranges can be presented for SE and SP, or for the number of FPs and FNs, given a particular pretest probability. In comparing two tests, one will rate the certainty of the difference in SE and SP or FPs and FNs between the tests under consideration. This approach could potentially mean that we express high certainty in very imprecise results(2).

Risk of bias: In addition to including appropriate design features of single test accuracy studies(3), comparative diagnostic test accuracy studies should ensure that participants are comparable in terms of factors that affect test accuracy.

Indirectness and applicability: The assessment of indirectness for comparative diagnostic test accuracy remains largely the same. The population, intervention test (index test), comparison test (alternative index test) and outcomes in the body of evidence should be similar to those of the healthcare question at hand.

Inconsistency: When rating inconsistency and imprecision for comparative diagnostic test accuracy, the study results and the threshold should be preferably expressed as comparative diagnostic test accuracy measures, rather than as the accuracy of each index test. We focus on the scenario in which a specific cut-off value is used for each test,

and therefore we are able to express test accuracy as SE and SP. Comparisons of SE and SP can be expressed as absolute differences, ratios or odds ratios(4).

Imprecision: Imprecise estimates, identifiable as wide CIs around the summary estimate, can reduce the certainty of evidence(5). How wide the interval should be to lower our certainty depends on the threshold used. If the CI crosses the threshold, it warrants rating down for imprecision. However, if the wide CI for the summary estimate is clearly due to between-study heterogeneity, one may choose rate down for inconsistency instead of imprecision.

Publication bias: Until further research regarding publication bias in comparative diagnostic test accuracy is available, raters should choose the prior GRADE guidance for judging publication bias(5). These include for-profit interest, knowledge of unpublished studies, and presence of small studies with results suggesting implausibly large difference in accuracy. In addition, tests for small-study effects in test accuracy reviews may also be used, but these have not been validated for comparative diagnostic test accuracy studies.

Upgrading for dose effect, large effects, residual plausible bias and confounding: The application of these principles to comparative diagnostic test accuracy evidence requires further investigation; here we offer temporary and limited guidance(6).

Between-study (indirect) comparisons

The approach to between-study comparisons, as described in GRADE guidance 21(7), largely remains the same, but there are additional considerations for the domains risk of bias, indirectness, and inconsistency.

Risk of bias: When assessing risk of bias in studies that evaluate the accuracy of a single test, appropriate tools should be used, such as QUADAS-2(3) . This will result in a risk of bias judgment for each test in the comparison. We suggest choosing the highest risk of bias judgment to represent the risk of bias for the comparison.

Indirectness and applicability: Indirectness in the context of between-study comparisons refers to obtaining accuracy estimates for each test from different bodies of

evidence and comparing them: a set of studies for DECUS and a fully separate set of studies for the enhance-CT, for example. While risk of bias can be addressed separately for the different bodies of evidence, we have an added concern regarding the indirect comparison with possible confounding due to differences in study group characteristics or in the reference standard. For this reason, between-study comparisons will typically lead to ratings lower than high certainty of the evidence.

Inconsistency: In between-study comparisons, we are not interested in the inconsistency in estimates of each test's accuracy, but rather in the inconsistency of their comparison. However, studies in a between-study comparison can only estimate test accuracy for a single test, and inconsistency in comparative diagnostic test accuracy estimates cannot be directly observed. GRADE Guidance 31 suggest a two-step procedure for assessing inconsistency for between-study comparisons: first rating inconsistency for each index test using established criteria(5) and second, inferring the inconsistency of their comparison. How the rating of inconsistency for each index test could be operationalized using thresholds is still a work in progress.

Imprecision: Imprecise estimates, identifiable as wide CIs around the summary estimate, can reduce the certainty of evidence(5). How wide the interval should be to lower our certainty depends on the threshold used(1, 2). If the CI crosses the threshold, it warrants rating down for imprecision. However, if the wide CI for the summary estimate is clearly due to between-study heterogeneity, one may choose rate down for inconsistency instead of imprecision.

Publication bias: Until further research regarding publication bias in comparative diagnostic test accuracy is available, raters should choose the prior GRADE guidance for judging publication bias(5). These include for-profit interest, knowledge of unpublished studies, and presence of small studies with results suggesting implausibly large difference in accuracy. In addition, tests for small-study effects in test accuracy reviews may also be used, but these have not been validated for comparative diagnostic test accuracy studies.

Upgrading for dose effect, large effects, residual plausible bias and confounding: The application of these principles to comparative diagnostic test accuracy evidence requires further investigation; here we offer temporary and limited guidance(6).

Supplementary Table 1. Search Strategy

Database/platforms	Results
Cochrane Library	75
PubMed	1084
Web of Science	2056
EMBASE	2276
TOTAL	5491

Database: Cochrane Library

Retrieval date: December 29, 2023. Results 75

#	Searches	Results
#1	(double contrast enhanced ultrasonography):ti,ab,kw OR (double contrast enhanced ultrasound):ti,ab,kw OR (DCEUS):ti,ab,kw	56
#2	MeSH descriptor: [Tomography Scanners, X-Ray Computed] explode all trees	49

#3	(X-Ray Computed Tomography):ti,ab,kw OR (Tomography, X-Ray Computerized):ti,ab,kw OR (Tomography, X Ray Computerized):ti,ab,kw OR (Computed X Ray Tomography):ti,ab,kw OR (X-Ray Computer Assisted Tomography):ti,ab,kw OR (X Ray Computer Assisted Tomography):ti,ab,kw OR (Tomography, X-Ray Computer Assisted):ti,ab,kw OR (Tomography, X Ray Computer Assisted):ti,ab,kw OR (Computerized Tomography, X Ray):ti,ab,kw OR (Computerized Tomography, X-Ray):ti,ab,kw OR (X-Ray Computerized Tomography):ti,ab,kw OR (CT X Ray):ti,ab,kw OR (CT X Rays):ti,ab,kw OR (X Ray, CT):ti,ab,kw OR (X Rays, CT):ti,ab,kw OR (Tomodensitometry):ti,ab,kw OR (Tomography, X Ray Computed):ti,ab,kw OR (X Ray Tomography, Computed):ti,ab,kw OR (X-Ray Tomography, Computed):ti,ab,kw OR (Computed X-Ray Tomography):ti,ab,kw OR (Tomographies, Computed X-Ray):ti,ab,kw OR (Tomography, Computed X-Ray):ti,ab,kw OR (Tomography, Xray Computed):ti,ab,kw OR (Computed Tomography, Xray):ti,ab,kw OR (Xray Computed Tomography):ti,ab,kw OR (CAT Scan, X Ray):ti,ab,kw OR (CAT Scan, X-Ray):ti,ab,kw OR (CAT Scans, X-Ray):ti,ab,kw OR (Scan, X-Ray CAT):ti,ab,kw OR (Scans, X-Ray CAT):ti,ab,kw OR (X-Ray CAT Scan):ti,ab,kw OR (X-Ray CAT Scans):ti,ab,kw OR (Tomography, Transmission Computed):ti,ab,kw OR (Computed Tomography, Transmission):ti,ab,kw OR (Transmission Computed Tomography):ti,ab,kw OR (CT Scan, X-Ray):ti,ab,kw OR (CT Scan, X Ray):ti,ab,kw OR (CT Scans, X-Ray):ti,ab,kw OR (Scan, X-Ray CT):ti,ab,kw OR (Scans, X-Ray CT):ti,ab,kw OR (X-Ray CT Scan):ti,ab,kw OR (X-Ray CT Scans):ti,ab,kw OR (Computed Tomography, X-Ray):ti,ab,kw OR (Computed Tomography, X Ray):ti,ab,kw OR (X Ray Computerized Tomography):ti,ab,kw OR (Cine-CT):ti,ab,kw OR (Cine CT):ti,ab,kw OR (Electron Beam Computed Tomography):ti,ab,kw OR (Electron Beam Tomography):ti,ab,kw OR (Beam Tomography, Electron):ti,ab,kw OR (Tomography, Electron Beam):ti,ab,kw OR (Tomography, X-Ray Computerized Axial):ti,ab,kw OR (Tomography, X Ray Computerized Axial):ti,ab,kw OR (X-Ray Computerized Axial Tomography):ti,ab,kw OR (X Ray Computerized Axial Tomography):ti,ab,kw OR (computed tomography):ti,ab,kw OR (contrast-enhanced computed tomography):ti,ab,kw OR (multidetector computed tomography):ti,ab,kw	23671
#4	#2 OR #3	23671
#5	#4 OR #1	23718
#6	MeSH descriptor: [Stomach Neoplasms] explode all trees	3551

#7	(Neoplasm, Stomach):ti,ab,kw OR (Stomach Neoplasm):ti,ab,kw OR (Neoplasms, Stomach):ti,ab,kw OR (Gastric Neoplasms):ti,ab,kw OR (Gastric Neoplasm):ti,ab,kw OR (Neoplasm, Gastric):ti,ab,kw OR (Neoplasms, Gastric):ti,ab,kw OR (Cancer of Stomach):ti,ab,kw OR (Stomach Cancers):ti,ab,kw OR (Gastric Cancer):ti,ab,kw OR (Cancer, Gastric):ti,ab,kw OR (Cancers, Gastric):ti,ab,kw OR (Gastric Cancers):ti,ab,kw OR (Stomach Cancer):ti,ab,kw OR (Cancer, Stomach):ti,ab,kw OR (Cancers, Stomach):ti,ab,kw OR (Cancer of the Stomach):ti,ab,kw OR (Gastric Cancer, Familial Diffuse):ti,ab,kw	11678
#8	#6 OR #7	11678
#9	MeSH descriptor: [Sensitivity and Specificity] explode all trees	19964
#10	MeSH descriptor: [Predictive Value of Tests] explode all trees	8771
#11	MeSH descriptor: [ROC Curve] explode all trees	8771
#12	MeSH descriptor: [Area Under Curve] explode all trees	1696
#13	(Sensitivity and Specificity):ti,ab,kw OR (Predictive Value of Tests):ti,ab,kw OR (Sensitivity):ti,ab,kw OR (idiosyncrasy):ti,ab,kw OR (accuracy):ti,ab,kw OR (degree of accuracy):ti,ab,kw OR (precision):ti,ab,kw OR (spot):ti,ab,kw OR (Positive predictive value):ti,ab,kw OR (Negative predictive value):ti,ab,kw OR (false positive rate):ti,ab,kw OR (false-negative rate):ti,ab,kw OR (Jordon index):ti,ab,kw OR (predictive value):ti,ab,kw OR (Saturation):ti,ab,kw OR (Specificity):ti,ab,kw OR (ROC Curve):ti,ab,kw OR (ROC Analysis):ti,ab,kw OR (Receiver Operating Characteristic):ti,ab,kw OR (ROC):ti,ab,kw OR (Area Under Curves):ti,ab,kw OR (AUC):ti,ab,kw OR (predictive value):ti,ab,kw OR (Predictive Value of Tests):ti,ab,kw	7943
#14	#9 OR #10 OR #11 OR #12 OR #13	161596
#15	#5 AND #8 AND #14	75

Database: PubMed

Retrieval date: December 29, 2023. Results 1084

#	Searches	Results
15	((((((Sensitivity and Specificity[Mesh]) OR (Predictive Value of Tests[Mesh])) OR (ROC Curve[Mesh])) OR (Area Under Curve[Mesh])) OR (Sensitivity and Specificity[Title/Abstract] OR Predictive Value of Tests[Title/Abstract] OR Sensitivity[Title/Abstract] OR idiosyncrasy[Title/Abstract] OR accuracy[Title/Abstract] OR degree of accuracy[Title/Abstract] OR precision[Title/Abstract] OR spot[Title/Abstract] OR Positive predictive value[Title/Abstract] OR Negative predictive value[Title/Abstract] OR false positive	1,084

	rate[Title/Abstract] OR false-negative rate[Title/Abstract] OR Jordon index[Title/Abstract] OR predictive value[Title/Abstract] OR Saturation[Title/Abstract] OR Specificity[Title/Abstract] OR ROC Curve[Title/Abstract] OR ROC Analysis[Title/Abstract] OR Receiver Operating Characteristic[Title/Abstract] OR ROC[Title/Abstract] OR Area Under Curves[Title/Abstract] OR AUC[Title/Abstract] OR predictive value[Title/Abstract] OR Predictive Value of Tests[Title/Abstract])) AND ((Stomach Neoplasms[MeSH Terms]) OR (Stomach Neoplasms[Title/Abstract] OR Neoplasm, Stomach[Title/Abstract] OR Stomach Neoplasm[Title/Abstract] OR Neoplasms, Stomach[Title/Abstract] OR Gastric Neoplasms[Title/Abstract] OR Gastric Neoplasm[Title/Abstract] OR Neoplasm, Gastric[Title/Abstract] OR Neoplasms, Gastric[Title/Abstract] OR Cancer of Stomach[Title/Abstract] OR Stomach Cancers[Title/Abstract] OR Gastric Cancer[Title/Abstract] OR Cancer, Gastric[Title/Abstract] OR Cancers, Gastric[Title/Abstract] OR Gastric Cancers[Title/Abstract] OR Stomach Cancer[Title/Abstract] OR Cancer, Stomach[Title/Abstract] OR Cancers, Stomach[Title/Abstract] OR Cancer of the Stomach[Title/Abstract] OR Gastric Cancer, Familial Diffuse[Title/Abstract]))) AND ((("Tomography, X-Ray Computed"[Mesh]) OR (X-Ray Computed Tomography[Title/Abstract] OR Tomography, X-Ray Computerized[Title/Abstract] OR Tomography, X Ray Computerized[Title/Abstract] OR Computed X Ray Tomography[Title/Abstract] OR X-Ray Computer Assisted Tomography[Title/Abstract] OR X Ray Computer Assisted Tomography[Title/Abstract] OR Tomography, X-Ray Computer Assisted[Title/Abstract] OR Tomography, X Ray Computer Assisted[Title/Abstract] OR Computerized Tomography, X Ray[Title/Abstract] OR Computerized Tomography, X-Ray[Title/Abstract] OR X-Ray Computerized Tomography[Title/Abstract] OR CT X Ray[Title/Abstract] OR CT X Rays[Title/Abstract] OR X Ray, CT[Title/Abstract] OR X Rays, CT[Title/Abstract] OR Tomodensitometry[Title/Abstract] OR Tomography, X Ray Computed[Title/Abstract] OR X Ray Tomography, Computed[Title/Abstract] OR X-Ray Tomography, Computed[Title/Abstract] OR Computed X-Ray Tomography[Title/Abstract] OR Tomographies, Computed X-Ray[Title/Abstract] OR Tomography, Computed X-Ray[Title/Abstract] OR Tomography, Xray Computed[Title/Abstract] OR Computed Tomography, Xray[Title/Abstract] OR Xray Computed Tomography[Title/Abstract] OR CAT Scan, X Ray[Title/Abstract] OR CAT Scan, X-Ray[Title/Abstract] OR CAT Scans, X-Ray[Title/Abstract] OR Scan, X-Ray CAT[Title/Abstract] OR Scans, X-Ray CAT[Title/Abstract] OR X-Ray CAT Scan[Title/Abstract] OR X-Ray CAT Scans[Title/Abstract] OR Tomography, Transmission Computed[Title/Abstract] OR Computed Tomography, Transmission[Title/Abstract] OR Transmission Computed Tomography[Title/Abstract] OR CT Scan, X-Ray[Title/Abstract] OR CT Scan, X Ray[Title/Abstract] OR CT Scans, X-Ray[Title/Abstract] OR Scan, X-Ray CT[Title/Abstract] OR Scans, X-Ray CT[Title/Abstract] OR X-Ray CT Scan[Title/Abstract] OR X-Ray CT Scans[Title/Abstract] OR Computed Tomography, X-Ray[Title/Abstract] OR Computed Tomography, X Ray[Title/Abstract] OR X Ray Computerized Tomography[Title/Abstract] OR Cine-CT[Title/Abstract] OR Cine CT[Title/Abstract] OR	

	Electron Beam Computed Tomography[Title/Abstract] OR Electron Beam Tomography[Title/Abstract] OR Beam Tomography, Electron[Title/Abstract] OR Tomography, Electron Beam[Title/Abstract] OR Tomography, X-Ray Computerized Axial[Title/Abstract] OR Tomography, X Ray Computerized Axial[Title/Abstract] OR X-Ray Computerized Axial Tomography[Title/Abstract] OR X Ray Computerized Axial Tomography[Title/Abstract] OR computed tomography[Title/Abstract] OR contrast-enhanced computed tomography[Title/Abstract] OR multidetector computed tomography[Title/Abstract])) OR (double contrast enhanced ultrasonography[Title/Abstract] OR double contrast enhanced ultrasound[Title/Abstract] OR DCEUS[Title/Abstract]))	
14	((((Sensitivity and Specificity[Mesh]) OR (Predictive Value of Tests[Mesh])) OR (ROC Curve[Mesh])) OR (Area Under Curve[Mesh])) OR (Sensitivity and Specificity[Title/Abstract] OR Predictive Value of Tests[Title/Abstract] OR Sensitivity[Title/Abstract] OR idiosyncrasy[Title/Abstract] OR accuracy[Title/Abstract] OR degree of accuracy[Title/Abstract] OR precision[Title/Abstract] OR spot[Title/Abstract] OR Positive predictive value[Title/Abstract] OR Negative predictive value[Title/Abstract] OR false positive rate[Title/Abstract] OR false-negative rate[Title/Abstract] OR Jordon index[Title/Abstract] OR predictive value[Title/Abstract] OR Saturation[Title/Abstract] OR Specificity[Title/Abstract] OR ROC Curve[Title/Abstract] OR ROC Analysis[Title/Abstract] OR Receiver Operating Characteristic[Title/Abstract] OR ROC[Title/Abstract] OR Area Under Curves[Title/Abstract] OR AUC[Title/Abstract] OR predictive value[Title/Abstract] OR Predictive Value of Tests[Title/Abstract])	2,543,461
13	Sensitivity and Specificity[Title/Abstract] OR Predictive Value of Tests[Title/Abstract] OR Sensitivity[Title/Abstract] OR idiosyncrasy[Title/Abstract] OR accuracy[Title/Abstract] OR degree of accuracy[Title/Abstract] OR precision[Title/Abstract] OR spot[Title/Abstract] OR Positive predictive value[Title/Abstract] OR Negative predictive value[Title/Abstract] OR false positive rate[Title/Abstract] OR false-negative rate[Title/Abstract] OR Jordon index[Title/Abstract] OR predictive value[Title/Abstract] OR Saturation[Title/Abstract] OR Specificity[Title/Abstract] OR ROC Curve[Title/Abstract] OR ROC Analysis[Title/Abstract] OR Receiver Operating Characteristic[Title/Abstract] OR ROC[Title/Abstract] OR Area Under Curves[Title/Abstract] OR AUC[Title/Abstract] OR predictive value[Title/Abstract] OR Predictive Value of Tests[Title/Abstract]	2,188,728
12	Area Under Curve[Mesh]	46,013
11	ROC Curve[Mesh]	71,839
10	Predictive Value of Tests[Mesh]	223,907
9	Sensitivity and Specificity[Mesh]	651,431
8	(Stomach Neoplasms[MeSH Terms]) OR (Stomach Neoplasms[Title/Abstract] OR Neoplasm, Stomach[Title/Abstract] OR Stomach	140,176

	Neoplasm[Title/Abstract] OR Neoplasms, Stomach[Title/Abstract] OR Gastric Neoplasms[Title/Abstract] OR Gastric Neoplasm[Title/Abstract] OR Neoplasm, Gastric[Title/Abstract] OR Neoplasms, Gastric[Title/Abstract] OR Cancer of Stomach[Title/Abstract] OR Stomach Cancers[Title/Abstract] OR Gastric Cancer[Title/Abstract] OR Cancer, Gastric[Title/Abstract] OR Cancers, Gastric[Title/Abstract] OR Gastric Cancers[Title/Abstract] OR Stomach Cancer[Title/Abstract] OR Cancer, Stomach[Title/Abstract] OR Cancers, Stomach[Title/Abstract] OR Cancer of the Stomach[Title/Abstract] OR Gastric Cancer, Familial Diffuse[Title/Abstract])	
7	Stomach Neoplasms[Title/Abstract] OR Neoplasm, Stomach[Title/Abstract] OR Stomach Neoplasm[Title/Abstract] OR Neoplasms, Stomach[Title/Abstract] OR Gastric Neoplasms[Title/Abstract] OR Gastric Neoplasm[Title/Abstract] OR Neoplasm, Gastric[Title/Abstract] OR Neoplasms, Gastric[Title/Abstract] OR Cancer of Stomach[Title/Abstract] OR Stomach Cancers[Title/Abstract] OR Gastric Cancer[Title/Abstract] OR Cancer, Gastric[Title/Abstract] OR Cancers, Gastric[Title/Abstract] OR Gastric Cancers[Title/Abstract] OR Stomach Cancer[Title/Abstract] OR Cancer, Stomach[Title/Abstract] OR Cancers, Stomach[Title/Abstract] OR Cancer of the Stomach[Title/Abstract] OR Gastric Cancer, Familial Diffuse[Title/Abstract]	97,019
6	Stomach Neoplasms[MeSH Terms]	112,091
5	(("Tomography, X-Ray Computed"[Mesh]) OR (X-Ray Computed Tomography[Title/Abstract] OR Tomography, X-Ray Computerized[Title/Abstract] OR Tomography, X Ray Computerized[Title/Abstract] OR Computed X Ray Tomography[Title/Abstract] OR X-Ray Computer Assisted Tomography[Title/Abstract] OR X Ray Computer Assisted Tomography[Title/Abstract] OR Tomography, X-Ray Computer Assisted[Title/Abstract] OR Tomography, X Ray Computer Assisted[Title/Abstract] OR Computerized Tomography, X Ray[Title/Abstract] OR Computerized Tomography, X-Ray[Title/Abstract] OR X-Ray Computerized Tomography[Title/Abstract] OR CT X Ray[Title/Abstract] OR CT X Rays[Title/Abstract] OR X Ray, CT[Title/Abstract] OR X Rays, CT[Title/Abstract] OR Tomodensitometry[Title/Abstract] OR Tomography, X Ray Computed[Title/Abstract] OR X Ray Tomography, Computed[Title/Abstract] OR X-Ray Tomography, Computed[Title/Abstract] OR Computed X-Ray Tomography[Title/Abstract] OR Tomographies, Computed X-Ray[Title/Abstract] OR Tomography, Computed X-Ray[Title/Abstract] OR Tomography, Xray Computed[Title/Abstract] OR Computed Tomography, Xray[Title/Abstract] OR Xray Computed Tomography[Title/Abstract] OR CAT Scan, X Ray[Title/Abstract] OR CAT Scan, X-Ray[Title/Abstract] OR CAT Scans, X-Ray[Title/Abstract] OR Scan, X-Ray CAT[Title/Abstract] OR Scans, X-Ray CAT[Title/Abstract] OR X-Ray CAT Scan[Title/Abstract] OR X-Ray CAT Scans[Title/Abstract] OR Tomography, Transmission Computed[Title/Abstract] OR Computed Tomography, Transmission[Title/Abstract] OR Transmission Computed Tomography[Title/Abstract] OR CT Scan, X-Ray[Title/Abstract] OR CT Scan, X Ray[Title/Abstract] OR CT Scans, X-Ray[Title/Abstract] OR Scan, X-Ray CT[Title/Abstract] OR	668,893

	Scans, X-Ray CT[Title/Abstract] OR X-Ray CT Scan[Title/Abstract] OR X-Ray CT Scans[Title/Abstract] OR Computed Tomography, X-Ray[Title/Abstract] OR Computed Tomography, X Ray[Title/Abstract] OR X Ray Computerized Tomography[Title/Abstract] OR Cine-CT[Title/Abstract] OR Cine CT[Title/Abstract] OR Electron Beam Computed Tomography[Title/Abstract] OR Electron Beam Tomography[Title/Abstract] OR Beam Tomography, Electron[Title/Abstract] OR Tomography, Electron Beam[Title/Abstract] OR Tomography, X-Ray Computerized Axial[Title/Abstract] OR Tomography, X Ray Computerized Axial[Title/Abstract] OR X-Ray Computerized Axial Tomography[Title/Abstract] OR X Ray Computerized Axial Tomography[Title/Abstract] OR computed tomography[Title/Abstract] OR contrast-enhanced computed tomography[Title/Abstract] OR multidetector computed tomography[Title/Abstract])) OR (double contrast enhanced ultrasonography[Title/Abstract] OR double contrast enhanced ultrasound[Title/Abstract] OR DCEUS[Title/Abstract])	
4	double contrast enhanced ultrasonography[Title/Abstract] OR double contrast enhanced ultrasound[Title/Abstract] OR DCEUS[Title/Abstract]	191
3	("Tomography, X-Ray Computed"[Mesh]) OR (X-Ray Computed Tomography[Title/Abstract] OR Tomography, X-Ray Computerized[Title/Abstract] OR Tomography, X Ray Computerized[Title/Abstract] OR Computed X Ray Tomography[Title/Abstract] OR X-Ray Computer Assisted Tomography[Title/Abstract] OR X Ray Computer Assisted Tomography[Title/Abstract] OR Tomography, X-Ray Computer Assisted[Title/Abstract] OR Tomography, X Ray Computer Assisted[Title/Abstract] OR Computerized Tomography, X Ray[Title/Abstract] OR Computerized Tomography, X-Ray[Title/Abstract] OR X-Ray Computerized Tomography[Title/Abstract] OR CT X Ray[Title/Abstract] OR CT X Rays[Title/Abstract] OR X Ray, CT[Title/Abstract] OR X Rays, CT[Title/Abstract] OR Tomodensitometry[Title/Abstract] OR Tomography, X Ray Computed[Title/Abstract] OR X Ray Tomography, Computed[Title/Abstract] OR X-Ray Tomography, Computed[Title/Abstract] OR Computed X-Ray Tomography[Title/Abstract] OR Tomographies, Computed X-Ray[Title/Abstract] OR Tomography, Computed X-Ray[Title/Abstract] OR Tomography, Xray Computed[Title/Abstract] OR Computed Tomography, Xray[Title/Abstract] OR Xray Computed Tomography[Title/Abstract] OR CAT Scan, X Ray[Title/Abstract] OR CAT Scan, X-Ray[Title/Abstract] OR CAT Scans, X-Ray[Title/Abstract] OR Scan, X-Ray CAT[Title/Abstract] OR Scans, X-Ray CAT[Title/Abstract] OR X-Ray CAT Scan[Title/Abstract] OR X-Ray CAT Scans[Title/Abstract] OR Tomography, Transmission Computed[Title/Abstract] OR Computed Tomography, Transmission[Title/Abstract] OR Transmission Computed Tomography[Title/Abstract] OR CT Scan, X-Ray[Title/Abstract] OR CT Scan, X Ray[Title/Abstract] OR CT Scans, X-Ray[Title/Abstract] OR Scan, X-Ray CT[Title/Abstract] OR Scans, X-Ray CT[Title/Abstract] OR X-Ray CT Scan[Title/Abstract] OR X-Ray CT Scans[Title/Abstract] OR Computed Tomography, X-Ray[Title/Abstract] OR Computed Tomography, X Ray[Title/Abstract] OR X Ray Computerized Tomography[Title/Abstract] OR	668,728

	Cine-CT[Title/Abstract] OR Cine CT[Title/Abstract] OR Electron Beam Computed Tomography[Title/Abstract] OR Electron Beam Tomography[Title/Abstract] OR Beam Tomography, Electron[Title/Abstract] OR Tomography, Electron Beam[Title/Abstract] OR Tomography, X-Ray Computerized Axial[Title/Abstract] OR Tomography, X Ray Computerized Axial[Title/Abstract] OR X-Ray Computerized Axial Tomography[Title/Abstract] OR X Ray Computerized Axial Tomography[Title/Abstract] OR computed tomography[Title/Abstract] OR contrast-enhanced computed tomography[Title/Abstract] OR multidetector computed tomography[Title/Abstract])	
2	X-Ray Computed Tomography[Title/Abstract] OR Tomography, X-Ray Computerized[Title/Abstract] OR Tomography, X Ray Computerized[Title/Abstract] OR Computed X Ray Tomography[Title/Abstract] OR X-Ray Computer Assisted Tomography[Title/Abstract] OR X Ray Computer Assisted Tomography[Title/Abstract] OR Tomography, X-Ray Computer Assisted[Title/Abstract] OR Tomography, X Ray Computer Assisted[Title/Abstract] OR Computerized Tomography, X Ray[Title/Abstract] OR Computerized Tomography, X-Ray[Title/Abstract] OR X-Ray Computerized Tomography[Title/Abstract] OR CT X Ray[Title/Abstract] OR CT X Rays[Title/Abstract] OR X Ray, CT[Title/Abstract] OR X Rays, CT[Title/Abstract] OR Tomodensitometry[Title/Abstract] OR Tomography, X Ray Computed[Title/Abstract] OR X Ray Tomography, Computed[Title/Abstract] OR X-Ray Tomography, Computed[Title/Abstract] OR Computed X-Ray Tomography[Title/Abstract] OR Tomographies, Computed X-Ray[Title/Abstract] OR Tomography, Computed X-Ray[Title/Abstract] OR Tomography, Xray Computed[Title/Abstract] OR Computed Tomography, Xray[Title/Abstract] OR Xray Computed Tomography[Title/Abstract] OR CAT Scan, X Ray[Title/Abstract] OR CAT Scan, X-Ray[Title/Abstract] OR CAT Scans, X-Ray[Title/Abstract] OR Scan, X-Ray CAT[Title/Abstract] OR Scans, X-Ray CAT[Title/Abstract] OR X-Ray CAT Scan[Title/Abstract] OR X-Ray CAT Scans[Title/Abstract] OR Tomography, Transmission Computed[Title/Abstract] OR Computed Tomography, Transmission[Title/Abstract] OR Transmission Computed Tomography[Title/Abstract] OR CT Scan, X-Ray[Title/Abstract] OR CT Scan, X Ray[Title/Abstract] OR CT Scans, X-Ray[Title/Abstract] OR Scan, X-Ray CT[Title/Abstract] OR Scans, X-Ray CT[Title/Abstract] OR X-Ray CT Scan[Title/Abstract] OR X-Ray CT Scans[Title/Abstract] OR Computed Tomography, X-Ray[Title/Abstract] OR Computed Tomography, X Ray[Title/Abstract] OR X Ray Computerized Tomography[Title/Abstract] OR Cine-CT[Title/Abstract] OR Cine CT[Title/Abstract] OR Electron Beam Computed Tomography[Title/Abstract] OR Electron Beam Tomography[Title/Abstract] OR Beam Tomography, Electron[Title/Abstract] OR Tomography, Electron Beam[Title/Abstract] OR Tomography, X-Ray Computerized Axial[Title/Abstract] OR Tomography, X Ray Computerized Axial[Title/Abstract] OR X-Ray Computerized Axial Tomography[Title/Abstract] OR X Ray Computerized Axial Tomography[Title/Abstract] OR computed tomography[Title/Abstract] OR contrast-enhanced computed	339,222

	tomography[Title/Abstract] OR multidetector computed tomography[Title/Abstract]	
1	"Tomography, X-Ray Computed"[Mesh]	497,433

Database: Web of Science

Retrieval date: December 29, 2023. Results 2056

#	Search Query	Results
1	TS=(Computed Tomography OR Tomography, X-Ray Computerized OR Tomography, X Ray Computerized OR Computed X Ray Tomography OR X-Ray Computer Assisted Tomography OR X Ray Computer Assisted Tomography OR Tomography, X-Ray Computer Assisted OR Tomography, X Ray Computer Assisted OR Computerized Tomography, X Ray OR Computerized Tomography, X-Ray OR X-Ray Computerized Tomography OR CT X Ray OR CT X Rays OR X Ray, CT OR X Rays, CT OR Tomodensitometry OR Tomography, X Ray Computed OR X Ray Tomography, Computed OR X-Ray Tomography, Computed OR Computed X-Ray Tomography OR Tomographies, Computed X-Ray OR Tomography, Computed X-Ray OR Tomography, Xray Computed OR Computed Tomography, Xray OR Xray Computed Tomography OR CAT Scan, X Ray OR CAT Scan, X-Ray OR CAT Scans, X-Ray OR Scan, X-Ray CAT OR Scans, X-Ray CAT OR X-Ray CAT Scan OR X-Ray CAT Scans OR Tomography, Transmission Computed OR Computed Tomography, Transmission OR Transmission Computed Tomography OR CT Scan, X-Ray OR CT Scan, X Ray OR CT Scans, X-Ray OR Scan, X-Ray CT OR Scans, X-Ray CT OR X-Ray CT Scan OR X-Ray CT Scans OR Computed Tomography, X-Ray OR Computed Tomography, X Ray OR X Ray Computerized Tomography OR Cine-CT OR Cine CT OR Electron Beam Computed Tomography OR Electron Beam Tomography OR Beam Tomography, Electron OR Tomography, Electron Beam OR Tomography, X-Ray Computerized Axial OR Tomography, X Ray Computerized Axial OR X-Ray Computerized Axial Tomography OR X Ray Computerized Axial Tomography OR computed tomography OR contrast-enhanced computed tomography OR multidetector computed tomography) and Preprint Citation Index (Exclude – Database)	981357
2	TS=(double contrast enhanced ultrasonography OR double contrast enhanced ultrasound OR DCEUS) and Preprint Citation Index (Exclude – Database)	476
3	TS=(Neoplasm, Stomach OR Stomach Neoplasm OR Neoplasms, Stomach OR Gastric Neoplasms OR Gastric Neoplasm OR Neoplasm, Gastric OR Neoplasms, Gastric OR Cancer of Stomach OR Stomach Cancers OR Gastric Cancer OR Cancer, Gastric OR Cancers, Gastric OR Gastric Cancers OR Stomach Cancer OR Cancer, Stomach OR Cancers, Stomach OR Cancer of the Stomach OR Gastric Cancer, Familial Diffuse) and Preprint Citation Index (Exclude – Database)	257325
4	TS=(Sensitivity and Specificity OR Predictive Value of Tests OR Sensitivity OR idiosyncrasy OR accuracy OR degree of accuracy OR	6199349

	precision OR spot OR Positive predictive value OR Negative predictive value OR false positive rate OR false-negative rate OR Jordon index OR predictive value OR Saturation OR Specificity OR ROC Curve OR ROC Analysis OR Receiver Operating Characteristic OR ROC OR Area Under Curves OR AUC OR predictive value OR Predictive Value of Tests) and Preprint Citation Index (Exclude – Database)	
5	#1 OR #2 and Preprint Citation Index (Exclude – Database)	981699
6	#3 AND #4 AND #5 and Preprint Citation Index (Exclude – Database)	2056

Database: embase

Retrieval date: December 29, 2023. Results 2276

No.	Query	Results
#15	#5 AND #8 AND #14	2276
#14	#9 OR #10 OR #11 OR #12 OR #13	3182258
#13	'sensitivity and specificity':ti,ab,kw OR 'predictive value of tests':ti,ab,kw OR 'sensitivity':ti,ab,kw OR 'idiosyncrasy':ti,ab,kw OR 'accuracy':ti,ab,kw OR 'degree of accuracy':ti,ab,kw OR 'precision':ti,ab,kw OR 'spot':ti,ab,kw OR 'positive predictive value':ti,ab,kw OR 'negative predictive value':ti,ab,kw OR 'false positive rate':ti,ab,kw OR 'false-negative rate':ti,ab,kw OR 'jordon index':ti,ab,kw OR 'saturation':ti,ab,kw OR 'specificity':ti,ab,kw OR 'roc curve':ti,ab,kw OR 'roc analysis':ti,ab,kw OR 'receiver operating characteristic':ti,ab,kw OR 'roc':ti,ab,kw OR 'area under curves':ti,ab,kw OR 'auc':ti,ab,kw OR 'predictive value':ti,ab,kw OR 'predictive value of tests'	2880576
#12	'area under the curve'/exp OR 'area under the curve'	249830
#11	'receiver operating characteristic'/exp OR 'receiver operating characteristic'	263608
#10	'predictive value'/exp OR 'predictive value'	327349
#9	'sensitivity and specificity'/exp OR 'sensitivity and specificity'	532651
#8	#6 OR #7	220352
#7	'neoplasm, stomach':ti,ab,kw OR 'stomach neoplasm':ti,ab,kw OR 'neoplasms, stomach':ti,ab,kw OR 'gastric neoplasms':ti,ab,kw OR 'gastric neoplasm':ti,ab,kw OR 'neoplasm, gastric':ti,ab,kw OR 'neoplasms, gastric':ti,ab,kw OR 'cancer of stomach':ti,ab,kw OR 'stomach cancers':ti,ab,kw OR 'gastric cancer':ti,ab,kw OR 'cancer, gastric':ti,ab,kw OR 'cancers, gastric':ti,ab,kw OR 'gastric cancers':ti,ab,kw OR 'stomach cancer':ti,ab,kw OR 'cancer, stomach':ti,ab,kw OR 'cancers, stomach':ti,ab,kw OR 'cancer of the stomach':ti,ab,kw OR 'gastric cancer, familial diffuse':ti,ab,kw	129354

#6	'stomach tumor'/exp	205682
#5	#3 OR #4	1496643
#4	'double contrast enhanced ultrasonography':ti,ab,kw OR 'double contrast enhanced ultrasound':ti,ab,kw OR dceus:ti,ab,kw	303
#3	#1 OR #2	1496406
#2	'x-ray computed tomography':ti,ab,kw OR 'tomography, x-ray computerized':ti,ab,kw OR 'tomography, x ray computerized':ti,ab,kw OR 'computed x ray tomography':ti,ab,kw OR 'x-ray computer assisted tomography':ti,ab,kw OR 'x ray computer assisted tomography':ti,ab,kw OR 'tomography, x-ray computer assisted':ti,ab,kw OR 'tomography, x ray computer assisted':ti,ab,kw OR 'computerized tomography, x ray':ti,ab,kw OR 'computerized tomography, x-ray':ti,ab,kw OR 'x-ray computerized tomography':ti,ab,kw OR 'ct x ray':ti,ab,kw OR 'ct x rays':ti,ab,kw OR 'x ray, ct':ti,ab,kw OR 'x rays, ct':ti,ab,kw OR tomodensitometry:ti,ab,kw OR 'tomography, x ray computed':ti,ab,kw OR 'x ray tomography, computed':ti,ab,kw OR 'x-ray tomography, computed':ti,ab,kw OR 'computed x-ray tomography':ti,ab,kw OR 'tomographies, computed x-ray':ti,ab,kw OR 'tomography, computed x-ray':ti,ab,kw OR 'tomography, xray computed':ti,ab,kw OR 'computed tomography, xray':ti,ab,kw OR 'xray computed tomography':ti,ab,kw OR 'cat scan, x ray':ti,ab,kw OR 'cat scan, x-ray':ti,ab,kw OR 'cat scans, x-ray':ti,ab,kw OR 'scan, x-ray cat':ti,ab,kw OR 'scans, x-ray cat':ti,ab,kw OR 'x-ray cat scan':ti,ab,kw OR 'x-ray cat scans':ti,ab,kw OR 'tomography, transmission computed':ti,ab,kw OR 'computed tomography, transmission':ti,ab,kw OR 'transmission computed tomography':ti,ab,kw OR 'ct scan, x-ray':ti,ab,kw OR 'ct scan, x ray':ti,ab,kw OR 'ct scans, x-ray':ti,ab,kw OR 'scan, x-ray ct':ti,ab,kw OR 'scans, x-ray ct':ti,ab,kw OR 'x-ray ct scan':ti,ab,kw OR 'x-ray ct scans':ti,ab,kw OR 'computed tomography, x-ray':ti,ab,kw OR 'computed tomography, x ray':ti,ab,kw OR 'x ray computerized tomography':ti,ab,kw OR 'cine ct':ti,ab,kw OR 'electron beam computed tomography':ti,ab,kw OR 'electron beam tomography':ti,ab,kw OR 'beam tomography, electron':ti,ab,kw OR 'tomography, electron beam':ti,ab,kw OR 'tomography, x-ray computerized axial':ti,ab,kw OR 'tomography, x ray computerized axial':ti,ab,kw OR 'x-ray computerized axial tomography':ti,ab,kw OR 'x ray computerized axial tomography':ti,ab,kw OR 'computed tomography':ti,ab,kw OR 'contrast-enhanced computed tomography':ti,ab,kw OR 'multidetector computed tomography':ti,ab,kw	436772
#1	'computer assisted tomography'/exp OR 'computer assisted tomography'	1450867

Supplementary Table 2. Characteristics of the included studies

Study	Country/Region	Edition of TNM Classification	Study Type	Numbers	Age, Years Mean (SD)	Female (%)	Type of diagnostic modality
Wu 2023(8)	China	The 8th AJCC	Prospective	108	N/A	29(26.85)	CT
Wang 2021(9)	China	The 8th UICC	Retrospective	206	59.7(11.3)	95(46.12)	DCEUS/CT
Gai 2021(10)	China	The 8th AJCC	Retrospective	109	51.37(11.45)	41(37.61)	CT
Zytoon 2020(11)	Egypt	N/A	Prospective	40	55((11.45)	15(37.5)	CT
Shen 2020(12)	China	N/A	Retrospective	59	51.41(10.67)	N/A	DCEUS/CT
Wang 2019(13)	China	N/A	Retrospective	158	59.5(10.6)	52(32.91)	DCEUS
He 2019(14)	China	The 7th AJCC	Retrospective	54	61(9.70)	18(33.33)	DCEUS/CT
Cimavilla-Román	Spanish	The 7th AJCC	Retrospecti	42	70.04(12.36)	16(38.1)	CT

2017(15)			ve				
Arslan 2017(16)	Turkey	The 7th AJCC	Retrospecti ve	51	61(7.83)	14(27.45)	CT
Zhao 2015(17)	China	The 7th AJCC	Retrospecti ve	153	50(27.5)	28(18.3)	CT
Yu 2015(18)	China	The 7th AJCC	Retrospecti ve	40	49(11.13)	13(32.5)	CT
Wang 2014(19)	China	The 7th AJCC	Retrospecti ve	194	56(7.89)	76(39.18)	CT
Feng 2013(20)	China	The 6th UICC	Retrospecti ve	610	57(10.05)	128(20.98)	CT
Zhong 2012(21)	China	The 7th AJCC	Retrospecti ve	120	61.4(8.17)	42(35)	CT
Li 2012(22)	China	The 6th UICC	Prospective	350	51.37(11.45)	105(30)	DCEUS
Zheng 2011(23)	China	The 7th AJCC/The 6th UICC	Retrospecti ve	162	58.3(8.50)	35(21.6)	DCEUS
Venkataraman 2010(24)	India	N/A	Prospective	42	51(9.88)	13(30.95)	CT

Pan 2010(25)	China	N/A	Prospective	350	52(8.06)	130(37.14)	CT
Hwang 2010(26)	Korea	N/A	Retrospective	277	53(49–56)*	106(38.27)	CT
Chen 2010(27)	China	N/A	Retrospective	143	56(11.4)	54(37.76)	DCEUS
Shin 2009(28)	Korea	The 5th UICC	Retrospective	1117	59.3 (10.67)	401(34.16)	CT
Wang 2009a(29)	China	N/A	Retrospective	34	61(10.28)	13(38.24)	CT
Wang 2009b(30)	China	The 6th UICC	Prospective	62	56.0(11.4)	24(38.71)	DCEUS
Huang 2009(31)	China	N/A	Retrospective	57	58(17.50)	22(38.6)	CT
Chao 2009(32)	China	The 4th UICC	prospective	790	58.3(8.5)	287(36.33)	CT
Qian 2008(33)	China	N/A	Retrospective	58	57.4(9.75)	22(37.93)	CT
Chamadol 2008(34)	Thailand	The 5th AJCC	Retrospective	28	54(15.25)	13(46.43)	CT

Yang 2007(35)	Korea	N/A	Retrospective	44	57(11.25)	10(22.73)	CT
Yan 2007(36)	China	The 4th UICC	Retrospective	220	55(11.75)	77(35)	CT
Chen 2007(37)	China	N/A	Retrospective	79	63(9.17)	33(41.77)	CT
Cao 2007(38)	China	N/A	Prospective	89	62(12.41)	40(44.94)	CT
Zhao 2006(39)	China	N/A	Retrospective	45	56(12)	14(31.11)	CT
Feng 2006(40)	China	N/A	Retrospective	58	62(32-8613.5)	27(46.55)	CT
Wei 2005(41)	China	N/A	Retrospective	59	58.3(10.5)	14(28)	CT
Polkowski 2004(42)	Polish	The 5th UICC	Retrospective	88	N/A	32（36.36）	CT
Habermann 2004(43)	Germany	N/A	Retrospective	51	62(7.25)	17(33.33)	CT

Lee 2001(44)	Korea	N/A	Prospective	43	57(12.25)	20(46.51)	CT
D'Elia 2000(45)	Italy	The 3th AJCC	Prospective	107	64(9.67)	34(31.78)	CT
Fukuya 1997(46)	Japan	N/A	Prospective	77	62.5(12.2)	25(32.47)	CT

* Mean (IQR),

R, retrospective study; P, prospective study; n/a, not available; IQR, interquartile range. UICC, Union for International Cancer Control; AJCC, American Joint Committee on Cancer

Supplementary Table 3. Characteristics of the data included in the study

Study	Type of diagnostic modality	T1				T2				T3				T4			
		TP	FP	FN	TN	TP	FP	FN	TN	TP	FP	FN	TN	TP	FP	FN	TN
Wu 2023(8)	CT	10	3	9	86	8	10	3	87	43	9	6	50	21	4	8	75
Wang 2021(9)	CT	3	1	8	194	20	5	16	165	8	68	11	119	83	9	57	57
	DCEUS	10	3	1	192	32	1	4	169	15	25	4	162	116	4	24	62
Gai 2021(10)	CT	15	4	10	80	21	9	10	69	26	10	10	63	13	5	4	87
Zytoon 2020(11)	CT	1	3	1	35	2	2	2	34	9	1	4	26	20	2	1	17
Shen 2020(12)	DCEUS	14	0	1	36	7	2	2	40	7	9	3	32	10	2	7	32
	CT	9	1	6	35	6	8	3	34	8	3	2	38	16	0	1	34
Wang 2019(13)	DCEUS	20	3	12	123	38	16	7	97	51	6	7	94	21	3	2	132
He 2019(14)	CT	6	2	2	44	4	3	3	44	24	6	3	21	10	1	2	41
	DCEUS	7	0	1	46	3	3	4	43	24	10	3	17	5	1	7	41
Cimavilla-Román 2017(15)	CT	7	3	1	31	1	10	1	30	11	7	13	11	2	1	6	33
Arslan 2017(16)	CT	n/a	n/a	n/a	n/a	7	9	4	31	23	4	8	16	6	2	3	40
Zhao 2015(17)	CT	4	2	8	139	20	11	9	113	27	20	10	96	58	11	17	67
Yu 2015(18)	CT	2	3	3	32	4	3	5	28	19	3	1	17	5	1	1	33

Wang 2014(19)	CT	16	8	14	156	12	10	12	160	12	30	2	150	104	2	22	66
Feng 2013(20)	CT	10	0	38	562	24	71	42	473	417	53	49	91	26	9	4	571
Zhong 2012(21)	CT	10	1	5	104	14	7	7	92	42	11	8	59	29	6	5	80
Li 2012(22)	DCEUS	0	0	0	350	90	3	18	241	175	24	20	131	41	17	6	286
Zheng 2011(23)	DCEUS	26	5	16	115	37	13	12	100	48	11	8	145	14	4	1	143
Venkataraman 2010(24)	CT	0	0	1	41	7	4	4	27	15	10	2	15	6	0	7	29
Pan 2010(25)	CT	30	2	18	300	37	28	25	260	115	42	20	173	86	10	19	235
Hwang 2010(26)	CT	47	9	134	87	22	7	49	199	20	33	2	222	1	2	2	272
Chen 2010(27)	DCEUS	n/a	n/a	n/a	n/a	41	2	10	90	61	13	6	63	22	4	3	114
Shin 2009(28)	CT	547	154	55	361	88	96	231	702	75	117	87	838	15	25	19	1058
Wang 2009a(29)	CT	2	0	1	31	9	4	1	20	12	2	3	17	4	1	2	27
Wang 2009b(30)	DCEUS	2	0	1	56	8	2	2	47	35	2	4	18	7	3	0	49
Huang 2009(31)	CT	1	0	3	53	4	2	1	50	18	4	5	30	22	4	3	28
Chao 2009(32)	CT	62	10	73	645	70	40	62	618	288	83	45	374	163	27	27	573
Qian 2008(33)	CT	5	2	3	48	24	5	6	23	11	4	4	39	5	2	0	51
Chamadol 2008(34)	CT	0	2	0	26	0	1	2	25	4	2	3	19	17	2	2	7
Yang 2007(35)	CT	15	0	2	25	7	3	1	31	15	1	2	24	0	1	0	41

Yan 2007(36)	CT	37	7	10	166	12	10	17	181	81	21	15	103	43	9	5	163
Chen 2007(37)	CT	18	2	6	38	10	12	2	40	15	3	8	38	4	0	1	59
Cao 2007(38)	CT	9	1	1	72	24	6	5	48	16	14	7	46	11	2	10	60
Zhao 2006(39)	CT	3	0	3	39	2	4	1	38	20	5	4	16	9	2	3	31
Feng 2006(40)	CT	5	3	2	40	3	6	5	36	6	4	6	34	19	4	4	13
Wei 2005(41)	CT	2	0	2	46	17	4	3	26	15	5	2	28	7	0	2	41
Polkowski 2004(42)	CT	2	1	9	76	2	21	7	58	28	16	22	22	7	11	11	59
Habermann 2004(43)	CT	n/a	n/a	n/a	n/a	24	5	5	17	12	5	7	27	3	2	0	46
Lee 2001(44)	CT	8	4	8	23	6	8	4	25	10	0	6	27	1	2	0	40
D'Elia 2000(45)	CT	3	0	12	92	29	13	6	59	36	6	5	60	15	1	1	90
Fukuya 1997(46)	CT	7	0	5	29	13	9	3	16	6	4	4	27	1	1	2	37

Supplementary Table 4. Specific parameters of CT scan

Study	CT device parameters	(Contrast agent type, dosage) /Injection rate	Scanning phase	Patient preparation	Scanning parameters	reconstruction parameters	The thickness of CT scan （mm）	The thickness of CT reconstructed slices（mm）
Wu 2023(8)	(Discovery CT750 HD, GE Healthcare)/64slice	（Optiray, 350 mg/mL）/ 3-4 mL/s	20-30s (arterial phase) /55-60s (intestinal phase)	fasted for 6h/ingest 1500 mL of water for gastric filling	120-kVtube voltage, automatic tube current (range: 80-260mA), 512 x 512 matrix and an appropriate field of view to fit the patient.	slice thickness is 5 mm/reconstructed thickness is 3 mm	5	3
Wang 2021(9)	(Philips Brilliance spiral CT) /256 slice	(iopromide，1.5mL/Kg)/ 3 mL/s	portal vein phase (20s after the arterial phase), and equilibrium phase (60s after the portal vein phase).	fast 8 hours/drink water 500 ml for gastric filling / intramuscular injection of raceanisodamine hydrochloride injection 10–20 mg	120 kV, 200–250 mAs, pitch 0.938, and collimation 0.625mm × 128.	N/a	N/a	N/a
Gai 2021(10)	(Siemens Definite spiral CT)/ 64 slice	(iopromide，1.5mL/Kg)/3.5 mL/s	After 30 s of contrast medium injection/injection of	fasted for 8 h / drank 800–1000 ml water for gastric filling/ injected	Parameters of scanning were set as voltage of 120 kV, current of 280	N/a	N/a	N/a

			contrast medium for 60 s	20 mg Anisodamine Hydrochloride	mA, slice thickness of 5 mm, and pitch of 1.25 mm.			
Zytoon 2020(11)	128 slice spiral CT	(nonionic iodine contrast agent，100mL)/3 mL/s	arterial phase (start delay of 30 s), in the portal venous phase (start delay of 75 s), and the equilibrium phase (start delay of 180 s)	fast 8 h / (Each patient received an intramuscular administration of 20 mg of anisodamine to decrease peristaltic bowel movement) / drink 1000–1200 ml water for gastric filling	128 detector rows used; pitch, 3; reconstruction interval, 2.5 mm; 200 mA; 120 kV; and tube rotation time, 0.8 s.	slice thickness is 5 mm/ multiplanar reconstruction had a section thickness of 2.5mm	5	2.5
Shen 2020(12)	Siemens 64 slice spiral CT	(Iohexol, 370 mgL/mL)/3-4 mL/s	The cortical phase scan was 25-30 s after injection.The scanning time of solid phase is 60-90 s after injection.	fasted 6 hours/ anisodamine was injected intramuscularly for 20 mg/ drink 1500 mL water to fully dilate the gastric cavity.	Tube voltage: 120 kV, tube current: 300 mAs, layer thickness: 5 mm, layer spacing: 5 mm.	slice thickness is 5 mm/ multiplane reconstruction reconstructed thickness is 5 mm	5	5
He 2019(14)	Siemens 256 slice spiral CT	(ioversol, 350 mgI/mL)/3 mL/s	N/a	fast 6 hours/ drink 600 to 1000 mL of water to dilate the stomach	N/a	N/a	N/a	N/a

Cimavilla-Román 2017(15)	(Siemens SOMATOM Definition MDCT scanner)/64-slice	(Ultravist 300 ,80-100 ml)/2 mL/s	a craniocaudal direction with a delay time of 35 and 70 seconds	N/a	tube voltage, 120 kVp; tube current,180mAs with automatic modulation; slice thickness, 1.25 (for a detectorconfiguration of 64 x 0.6 mm); rotation time, 0.6 sec; and pitch, 1.2.A convolution kernel (B30) and a 512 x 512 pixel matrix were used for image reconstruction.	Axial images were reconstructed with a slice thickness of 3 mm and a reconstruction increment of 1.5 mm. Sagittal and coronal images were reconstructed with a slice thickness of 1.5 mm.	3	1.5
Arslan 2017(16)	(Siemens SOMATOM Sensation 16)16-slice	(Omnipaque 350,80-100 ml)/2 mL/s	Approximately 70 seconds after the start of contrast medium administration	8 hours fasts/ drink water 1000 ml for gastric filling	kV was 120 and mAs was 150	slice thickness is 5 mm/ reconstituted with 1 mm thick pieces, at a reconstruction interval of 1 mm.	2.5	1
Zhao 2015(17)	（Siemens Definition Flash CT）256-slice	(The nonionic iodine-containing contrast agent Iohexol,300 mg/ml，90 ml)/3 mL/s	Scanning is performed at 35 seconds and 70 seconds respectively after injecting the contrast agent,Obtain the images of the	Fasting 8 hours/Inject 10 mg of anisodamine hydrobromide (654-2) intramuscularly/ 800 to 1000 ml of water for gastric filling	The two tube voltages are 100 kV and 140 kV respectively. The scanning slice thickness is 5 mm, and the reconstruction is carried out with the standard algorithm, with the	slice thickness is 5 mm, and the multiplanar reconstruction is 1 mm.	5	1

			arterial phase and the venous phase.		reconstruction slice thickness being 1.0 mm.			
Yu 2015(18)	(GE Lightspeed ，spiral CT)/16-slice	N/a	N/a	15 mg of anisodamine was intramuscularly injected/ take 2 to 3 g of gasgenerating agent to expand the stomach cavity	N/a	N/a	N/a	N/a
Wang 2014(19)	(LightSpeed VCT CT)/16-slice	N/a	N/a		N/a	N/a	N/a	N/a
Feng 2013(20)	（Philips Brilliance TM16，spiral CT）/ 16-slice	(1.5 ml iopromide per kilogram of body weight）90 ml)/2.5 mL/s	Dual-phasic helical scans were obtained at 25 seconds (arterial phase) and 50 seconds (portal-venous phase)	liquid diet on the day prior to examination/ received 600-800 ml water for gastric filling	120 kV, 250mA	N/a		
Zhong 2012(21)	64-slice spiral CT	(Contrast medium, 100 milliliters)/3 mL/s	The scanning of the arterial phase starts at a rate of 3 ml per second, 30 seconds after injecting the contrast agent. The parenchymal phase begins 60 seconds later, and the	In a fasting state/intramuscular injection of 10 milligrams of scopolamine hydrobromide. /ingest between 2000 and 2500 milliliters of water.	120 kV, 200-300 mA, slice thickness/slice interval: 3.75 mm or 5.0 mm, pitch: 1.375:1	N/a	5	5

Venkataraman 2010(24)	（Siemens Somatom Plus 4，CT）4-slice	(76% meglumine diatrizoate，60 ml)/3 mL/s	equilibrium phase starts 120 seconds after the injection of the contrast agent. Helical CT was performed from the diaphragmatic dome to the pelvis during a single breath-hold of 25 seconds	800 ml of water for gastric filling	N/a		N/a	N/a
Pan 2010(25)	（GE-Lightspeed CT scanner）/16-slice ；(GE Medical Systems)/16-slice	(iopromide,180 ml)/initial phase (100ml)/3ml/s and a second phase (80 ml) at a flow rate of 1ml/s for maintenance	Three CT acquisitions are obtained at 30 seconds, 60 seconds, and 180 seconds.	fasting 8 hours/ingest 1,000 ml of water /inject with a hypotonic agent (20 mg of scopolamine)	120 kV and 150 mA	Images in arterial and parenchymal phases were reconstructed into thin slices (2.5 mm) for further MIP reconstruction	5	2.5
Hwang 2010(26)	16 or 64 detector row scanners (Mx8000 IDT 16 and Brilliance 64（Philips Medical Systems)	(iopromide，2mL/Kg)/3 mL/s	N/a	Each patient drank 500–1000 mL tap water.	N/a	N/a	N/a	N/a
Shin 2009(28)	（16-channel MDCT MX8000; Philips Medical Systems)/16-slice	N/a	N/a		N/a		N/a	N/a

Wang 2009a(29)	GE Lightspeed 16-slice CT Scanner	(Omnipaque, 100 mL in volume, with a concentration of 300 mg/mL)/3mL/s	N/a	fast for 4 to 6 hours / orally take 800 to 1000 milliliters of clear water to fully distend the gastric cavity/ intramuscularly inject 10 milligrams of anisodamine (654-2) to reduce the tension of the gastrointestinal tract	120 kilovolts (KV), 350 milliamperes (mA), slice thickness of 3.75 millimeters, reconstruction interval of 1.25 millimeters, pitch of 1.375:1, matrix of 512×512	N/a		
Huang 2009(31)	（Philips brilliance 64 spiral CT）/16-slice ；（Philips brilliance16 spiral CT）/16-slice	(ltravist，300 mg I / mL)/3mL/s	The arterial phase and venous phase scans are carried out at 25 seconds and 50 seconds respectively after the injection.	Fast 8 hours/ orally take 500 to 1000 milliliters of water.	120 kilovolts (kV), 90 milliamperes (mA), the scanning time is 0.5 seconds per revolution, the table speed is 30 millimeters per revolution, and the pitch is 7.	The thickness of the transverse reconstruction slices was 1.0 mm, with a slice interval of 1.0 mm. The thickness of the Multi-Planar Reconstruction (MPR) slices was 2.0 mm, and the slice interval was 1.0 mm.	1	1

Chao 2009(32)	(LightSpeed QXi; GE Medical Systems)/ 4-slice	(nonionic iodine contrast agentUltravist，100ml)/3mL/s	CT acquisitions were performed in the arterial phase (start delay of 30 sec), in the portal venous phase (start delay of 75 sec), and in the equilibrium phase (start delay of 180 sec).	fast 8 hours/ intramuscular administration of 20 mg of anisodamine to decrease peristaltic bowel movement / ingest 1,000–1,200 ml water for gastric filling	four detector rows used; pitch, 3; reconstruction interval 2.5 mm; 200 mA; 120 kV; and tube rotation time, 0.8 sec.	section thickness of 3.75 mm/multiplanar reconstruction (MPR) images.	3.75	2.5
Qian 2008(33)	（GE Lightspeed 16 layer Spiral CT）	1.5ml/kg injection of iohexol, a Nonionic contrast agent, through the anterior elbow vein at the rate of 3ml/s	The images were scanned in arterial phase (35s after injection), portal phase (50s in parenchyma phase) and delayed phase (100s).	N/a	N/a	N/a	N/a	N/a
Chamadol 2008(34)	multi-slice CT scanner (Siemens, Somatome Volume Zoom)16-slice	(Non-ionic,iodinated contrast material, 300 mg/L,100 mL) / 2.5-3 mL/s	Contrastenhanced CT images were obtained at 30 sec for the arterial phase and 70 sec for the portal venous phase.	Fast 6 hours/ ingest 800–1,000 ml water for gastric filling	The abdomen was scanned in the supine position with 8-mm thick, continuous slices, from the dome of the diaphragm to the end of the lower pole of the kidney.	N/a	8	N/a

Yang 2007(35)	(SOMATOM Sensation 64; Siemens Medical System)/64-slice	(nonionic contrast media iopromide, Ultravist 370, 100 mL) / 4 mL/s	Scanning was performed during the arterial and portal venous phases, as determined with bolus tracking and automated triggering technology.	ingested 8 g of effervescent granules along with 10 mL of water to distend the stomach.	Scanning parameters were as follows: number of slices per rotation, 32 2; slice collimation, 0.6 mm; slice width, 5mm; feed/rotation, 23mm; pitch factor, 1.2; kernel, B30f, and gantry speed, 0.5 second per rotation.	The transverse section data were reconstructed twice, first with 5-mm thick sections at 5-mm intervals in the transverse plane and then with 1-mm thick sections at 1-mm intervals in the transverse plane. The second set of reconstructed transverse scans was reformatted in the coronal and oblique coronal plane with 3to 5-mm sections at 3- to 5-mm intervals.	N/a	N/a
Yan 2007(36)	N/a	N/a	N/a	N/a	N/a	N/a	N/a	N/a
Chen 2007(37)	(LightSpeed QXi; GE Medical Systems Milwaukee,)/16-slice	(contrast material,100mL)/ 3mL/s.	was initiated 50 seconds after the start of injection of contrast material for evaluation	overnight fasting / Butyl scopolamine (10mg) was administered intravenously to reduce bowel peristalsis/ ingest two packs of effervescent granules with minimal water to obtain gastric distension.	included 16 × 1.25-mm beam collimation, 1.2mm slice thickness, 2.5 mm reconstruction interval, a pitch of 6, 0.8 seconds gantry rotation time, table feed of 7.5 mm/rotation, field of view to fit the size of	(GE Medical Systems) for 3D reconstruction. Orthogonal sectional images of 0.6 mm thickness	1.2	2.5

					the individual patient (usually around 30–35cm), 120kVp and 80mA.			
Cao 2007(38)	(Light Speed 16 CT01-Oc0 Spiral CT)/16-slice	(Onepike 300 mg I/ml, 80-90ML)/3ml/s.	Arterial phase (20 s) and portal phase (70 s) images	Fasted 12 hours/ 20 mg scopolamine was injected intramuscularly/ingest 1,000 ml water for gastric filling/ If the patient has gastric retention, the gastric tube should be placed 2 days before the examination. The stomach was washed with concentrated sodium chloride solution, and the gastric fluid was sucked out through the gastric tube after examination. Patients were given breath-holding guidance before CT examination.	Voltage 120 kV, current 280 mA, pitch 1.375 mm, layer thickness 7.5 mm	Arterial phase and portal venous phase images were reconstructed into 2.5 IBm slice thickness images by MSCT server. All the image guide workstations were processed with ADW4.0 software, followed by multi-planar reconstruction (multiplanar reconstruction,MPR), volume reproduction technique (volume rendering,VR) and so on.	7.5	2.5
Zhao 2006(39)	(Toshiba Xpress/sx Spiral CT Scanner)/1-slice	(Ioversol Injection with an iodine concentration of 300 g/L, 1.5mL/Kg) / 3mL/s	The scanning of the arterial phase starts 27 seconds after the contrast agent is	fast for 8 to 12 hours /ingest 800-1,000 ml water for gastric filling	The scanning slice thickness is 0.5 cm. The scanning conditions are 120 kV, 250 mAs, the	N/a	N/a	N/a

			injected into the elbow vein, and the scanning of the venous phase starts 60 seconds after the injection.		matrix is 512x512, and the pitch is 1.			
Feng 2006(40)	(GE LightSpeed 16 CT Scanner)/16-slice	(Omnipaque with an iodine concentration of 300 mg/ml, 80 - 90 ml)/ 3 ml/s.	After enhancement, images of the arterial phase (at 20 seconds) and the portal venous phase (at 70 seconds) are acquired respectively.	Fasted 12 hours/administer an intramuscular injection of 20 mg of scopolamine (except for those with contraindications)/ ingest 1,000 ml water for gastric filling	The scanning parameters are as follows: tube voltage 120 kV, tube current 280 mA, slice thickness 7.5 mm, pitch 1.375:1, window width 400 Hu, and window level 40 Hu.	N/a	5	
Wei 2005(41)	(GE Hispeed CT/i)/ 1-slice	injection of of contrast material (Omnipaque, Schering,80-100 mL)/ 2.5-3.0 mL/s	30, 60-70, 150-180 s after contrast material was injected.	Ingest 500-1 000 mL of water for gastric filling/ 20-40 mg of anisodamine intramuscularly to minimize peristalsis/ An additional 500 mL of water was then offered to improve distension of the proximal part of the stomach.	It started above the diaphragm to cover the gastric cardia by obtaining contrast images with 5- or 7-mm collimation, a pitch of 1.5:1.0 or 1.1:1.0 (120 kV, 210 mA), and a matrix size of 512×512, then 3-mm axial scans were reconstructed for	N/a	N/a	N/a

					image evaluation.			
Polkowski 2004(42)	(Elscint Elscint CT Twin Flash scanner with two rows ofdetectors)2-slice	(Iohexol/Omnipaque 350 mg/ml,100ml)/3.5ml/s	Following the contrast injection, two-phase contrast-enhanced CT scanning was carried out in the arterial dominant phase (delay 25 s, pitch 1, slice thickness 2.5 mm) and in the parenchymal phase (delay 40±95 s, pitch 1.5, slice thickness 5 mm).	Ingest 800mL of water for gastric fillin/ administration of 20mg of scopolamine butylbromide.	N/a	N/a	5	N/a
Habermann 2004(43)	(Siemens Somatom Plus 4)/1-slice	(iodinated contrast material iopamidol Solutrast 300,120mL)/ 2.5 mL/s	30 seconds (arterial phase) and 70 seconds (portal-venous phase)	fast for 6 hours / intravenous infusion of 20 mg scopolamine butylbromide / Ingest 500-800 mL of water for gastric filling	120 kV, 200–240 mAs	The imaging parameters for the early phase were 3-mm collimation, 5 mm/sec table speed, and 3-mm reconstruction interval.		

Lee 2001(44)	(Siemens Somatom Plus 4)/1-slice	(68% ioversol Optiray 320, 120 ml)/ 3 ml/s.	the start of infusion of contrast material for the arterial phase, after 75 s for the parenchymal phase, and after 180 s for the delayed phase.	Ingest 500 mL of water for gastric filling/Twenty milligrams of scopolamine butylbromide was slowly injected intravenously in order to reduce gastric peristalsis.	120 kVp, 233 mA, 5 mm collimation and 7.5 mm/s table feed	N/a	5	5
D'Elia 2000(45)	(GE Medical Systems CT Sitec 3000)/1-slice	(non-ionic contrast medium Iopamiro, 200 ml) was administered by power injector using a biphasic technique: 100 ml in rapid bolus at a flow rate of 4 ml/s followed by continuous infusion of 100 ml at a flow rate of 1ml/s, through a 20-F plastic catheter placed in antecubital vein.	The examination began 45 s from the start of bolus infusion.	fast for 5 hours/Ingest 400±600 mL of water for gastric filling/ intravenous infusion of 20 mg of scopolamine butylbromide	120 kV, 130 mAs time of scan 1.8 s with delay of 3 s for table-incremental time, matrix 512 512, scan thickness 10 mm with 10-mm intervals; additional scans of 5 mm thickness were performed when the gastric cancer was smaller than 3 cm.	N/a		
Fukuya 1997(46)	CT scanner (CT scanner Toshiba 900S)/1-slice	(nonionic contrast material lopamiron 300,100 ml)/3 ml/s	Scans were started 40s after the initiationof the injection of intravenous contrast	Ingest 400 mlwater for gastric filling/ 20 mg of scopolaminebutyl bromide was injected intramuscularly.	120 kV and 150 mA	Image reconstruction was performed at 2.5-mm intervals.Based on the volumetric data obtained by helical scanning, axial	5	2.5

	material.	images of 5-mm section thickness at 5-mm intervals were printed on hard copies. MPR was also performed at the CT console using the software facilitated in the CT scanner.

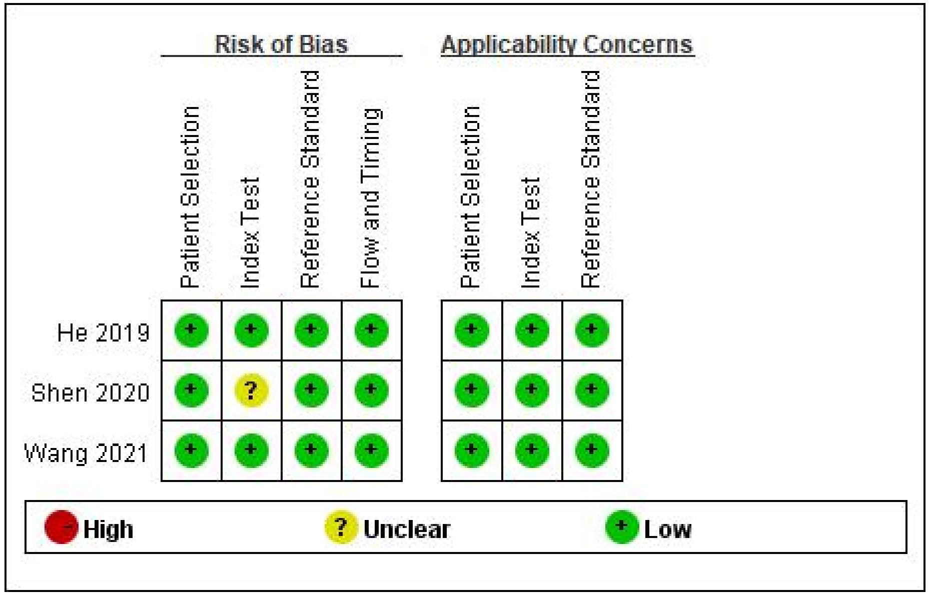

Supplementary Figure 2A. Quality of the 3 included studies (Evaluated by QUADAS-C)

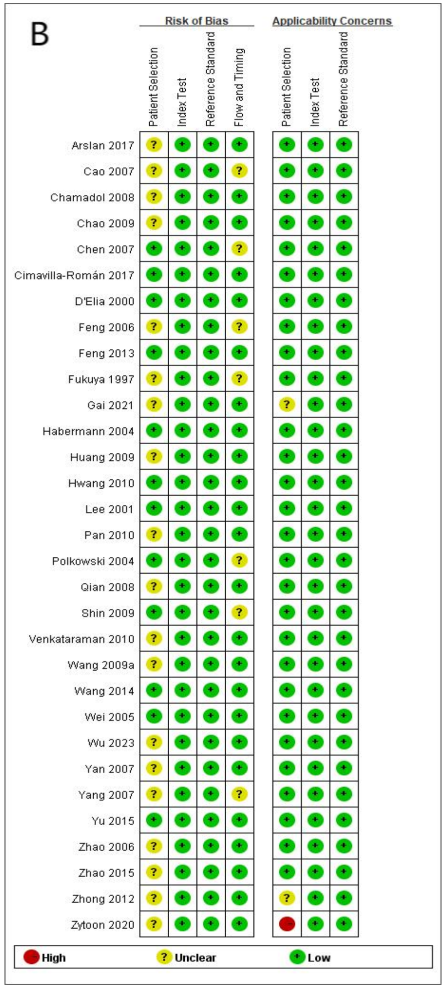

Supplementary Figure 2B. Quality of the 31 Single diagnostic test accuracy studies (Evaluated by QUADAS-2)

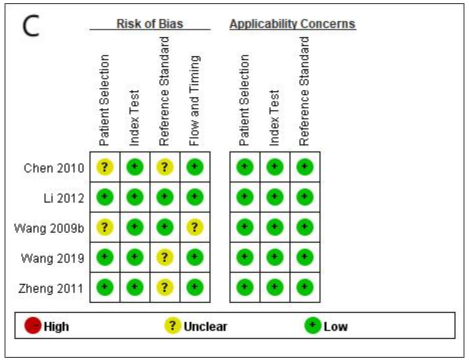

Supplementary Figure 2C. Quality of the 5 Single diagnostic test accuracy studies (Evaluated by QUADAS-2).

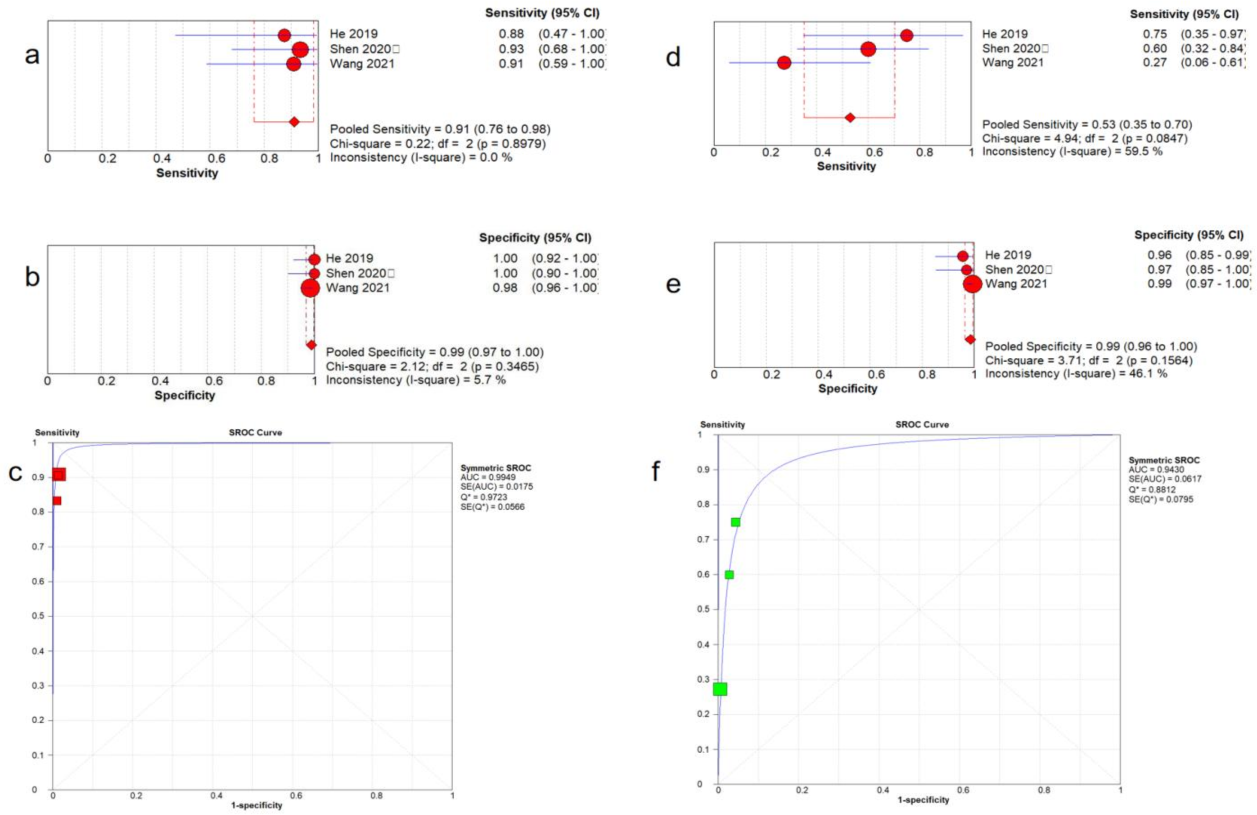

Supplementary Figure 3. Forest plots of sensitivity, specificity and SROC for DCEUS and enhanced-CT in T1 staging CDTA

SE (a), SP (b), and SROC (c) for DCEUS

SE (d), SP (e), and SROC (f) for enhanced-CT

SE: sensitivity; SP: specificity; CT, computed tomography; DCEUS, double contrast-enhanced ultrasound; CDTA, comparative diagnostic test accuracy studies

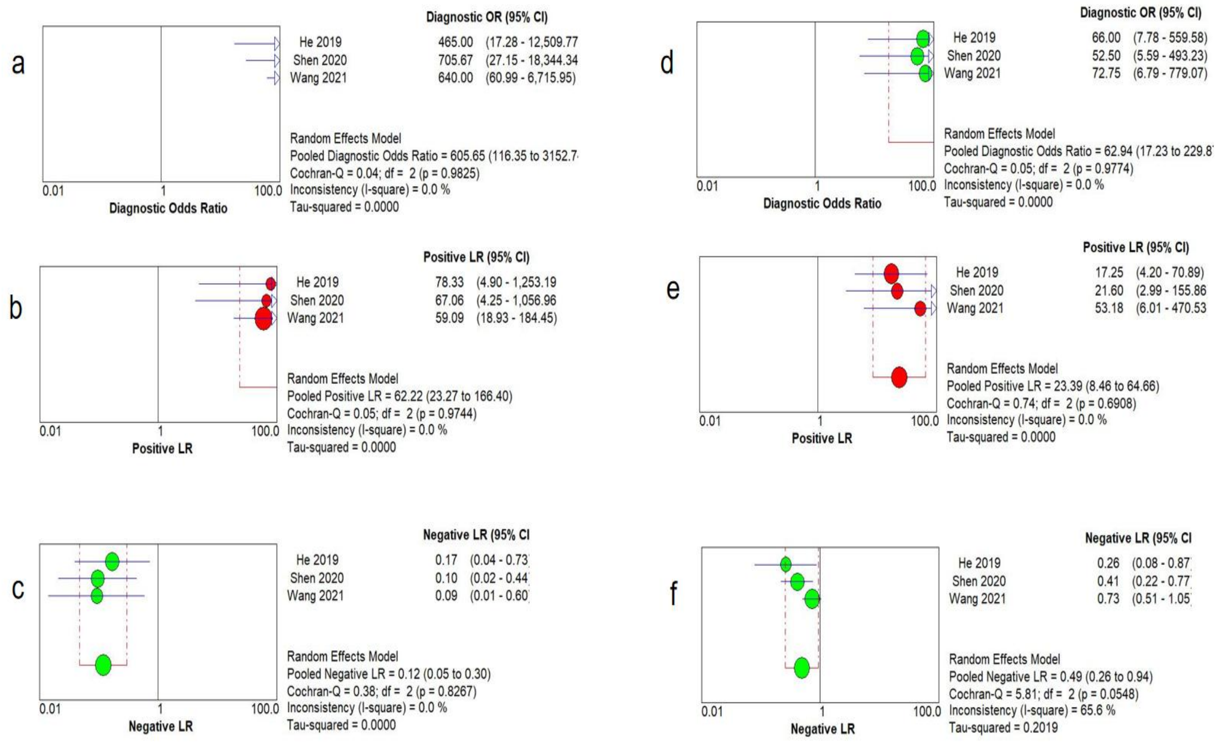

Supplementary Figure 4. Forest plots of DOR, PLR and NLR for DCEUS and enhanced-CT in T1 staging CDTA
DOR (a), PLR (b), and NLRC (c) for DCEUS DOR (d), PLR (e), and NLRC (f) for enhanced-CT
DOR: diagnostic odds ratio; PLR: positive likelihood ratio; NLR: negative likelihood ratio; CT, computed tomography; DCEUS, double contrast-enhanced ultrasound

A

Study or Subgroup	DCEUS Mean	DCEUS SD	DCEUS Total	enhanced-CT Mean	enhanced-CT SD	enhanced-CT Total	Weight	Mean Difference IV, Random, 95% CI
He 2019	0.88	0.99	54	0.75	1.16	54	28.0%	0.13 [-0.28, 0.54]
Shen 2020	0.93	0.63	59	0.6	1.02	59	38.0%	0.33 [0.02, 0.64]
Wang 2021	0.91	1.5	206	0.27	2.01	206	34.0%	0.64 [0.30, 0.98]
**Total (95% CI)**			**319**			**319**	**100.0%**	**0.38 [0.10, 0.66]**

Heterogeneity: Tau² = 0.03; Chi² = 3.76, df = 2 (P = 0.15); I² = 47%
Test for overall effect: Z = 2.69 (P = 0.007)

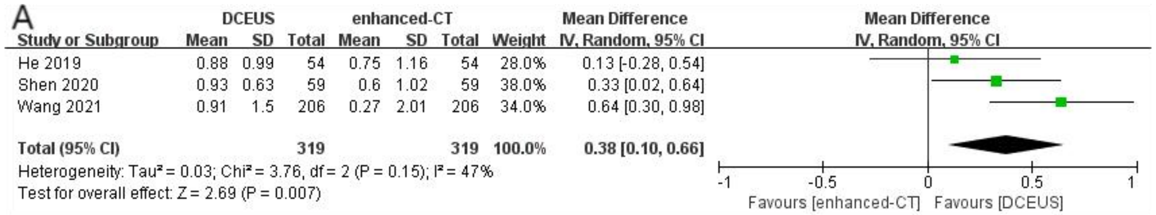

B

Study or Subgroup	DCEUS Mean	DCEUS SD	DCEUS Total	enhanced-CT Mean	enhanced-CT SD	enhanced-CT Total	Weight	Mean Difference IV, Random, 95% CI
He 2019	1	0.15	54	0.96	0.26	54	8.5%	0.04 [-0.04, 0.12]
Shen 2020	1	0.2	59	0.97	0.29	59	6.8%	0.03 [-0.06, 0.12]
Wang 2021	0.98	0.15	206	0.99	0.11	206	84.7%	-0.01 [-0.04, 0.02]
**Total (95% CI)**			**319**			**319**	**100.0%**	**-0.00 [-0.03, 0.02]**

Heterogeneity: Tau² = 0.00; Chi² = 1.92, df = 2 (P = 0.38); I² = 0%
Test for overall effect: Z = 0.25 (P = 0.80)

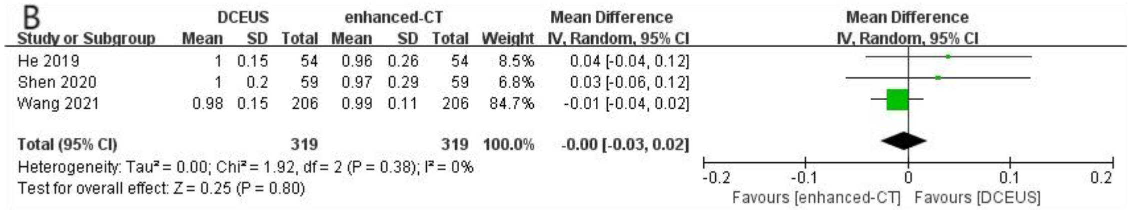

Supplementary Figure 5. Forest plots of MD for sensitivity and specificity in T1 staging CDTA sensitivity (A), specificity (B)

MD, mean difference; CI, confidence interval

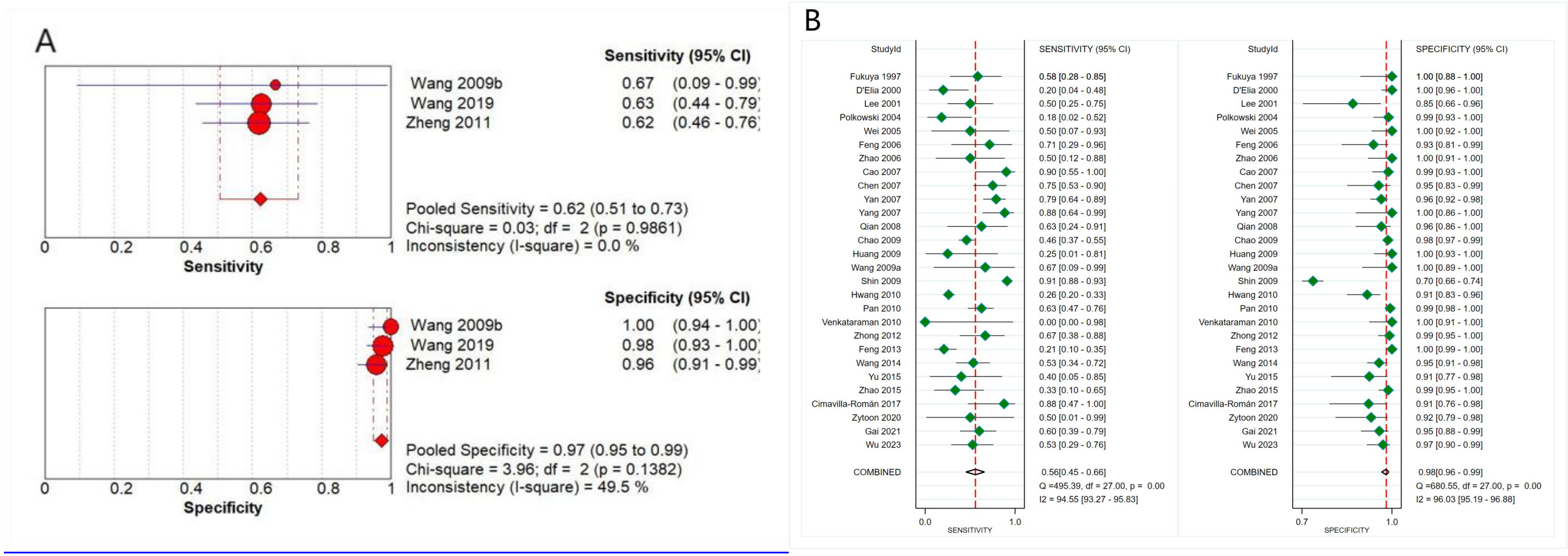

Supplementary Figure 6. Forest plots of sensitivity and specificity for DCEUS and enhanced-CT in T1 staging among SDTA

DCEUS (A), enhanced-CT (B)

CT, computed tomography; DCEUS, double contrast-enhanced ultrasound

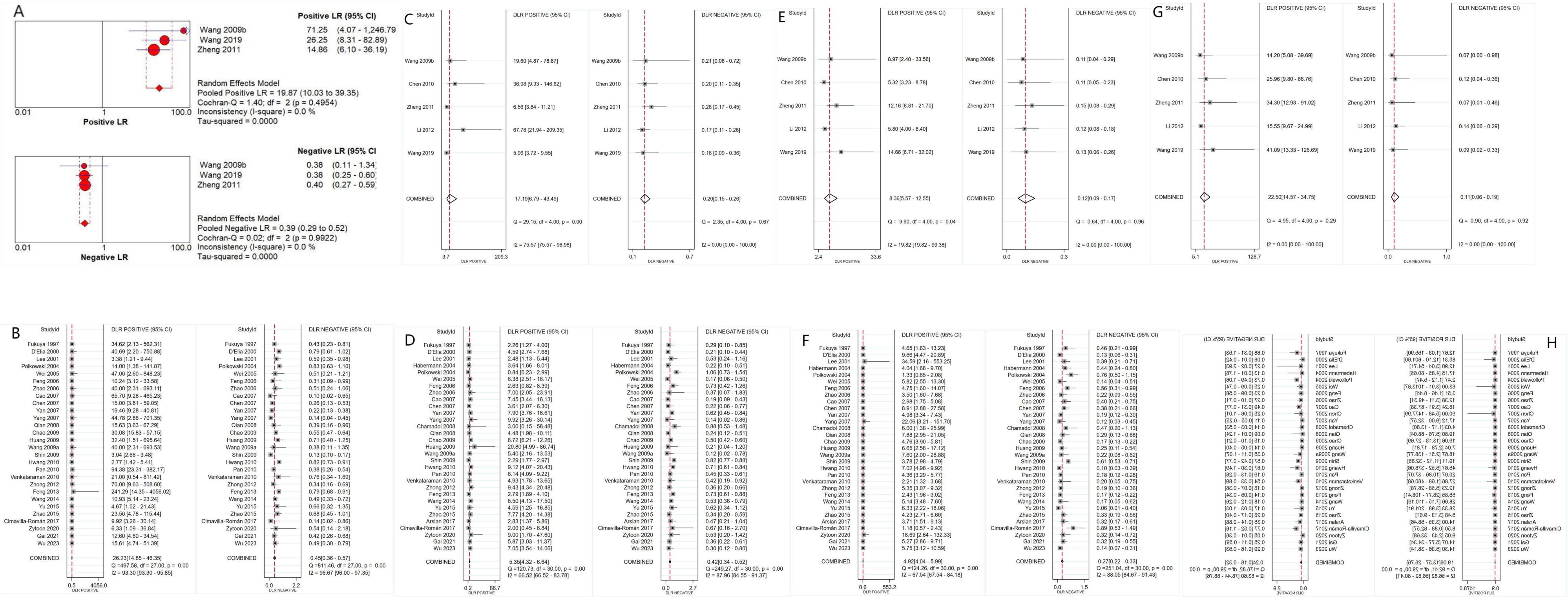

A
Positive LR (95% CI)
Wang 2009b 71.25 (4.07 - 1,246.79
Wang 2019 26.25 (8.31 - 82.89)
Zheng 2011 14.86 (6.10 - 36.19)
Random Effects Model
Pooled Positive LR = 19.87 (10.03 to 39.35)
Cochran-Q = 1.40; df = 2 (p = 0.4954)
Inconsistency (I-square) = 0.0 %
Tau-squared = 0.0000
0.01
1
100.0
Positive LR
Negative LR (95% CI
Wang 2009b 0.38 (0.11 - 1.34
Wang 2019 0.38 (0.25 - 0.60
Zheng 2011 0.40 (0.27 - 0.59
Random Effects Model
Pooled Negative LR = 0.39 (0.29 to 0.52)
Cochran-Q = 0.02; df = 2 (p = 0.9922)
Inconsistency (I-square) = 0.0 %
Tau-squared = 0.0000
Negative LR
B
C
D
E
F
G
H

Supplementary Figure 7. Likelihood forest plots

(A) DCEUS in T1 staging; (B) enhanced-CT forT1 staging; (C) DCEUS in T2 staging; (D) enhanced-CT in T2 staging; (E) DCEUS in T3 staging; (F) enhanced-CT in T3 staging; (G) DCEUS in T4 staging; (H) enhanced-CT in T4 staging.

Summary LR+ and LR-for index test with 95% confidence intervals. DLR, Diagnosis likelihood ratio; CT, computed tomography; DCEUS, Double Contrast-Enhanced Ultrasound

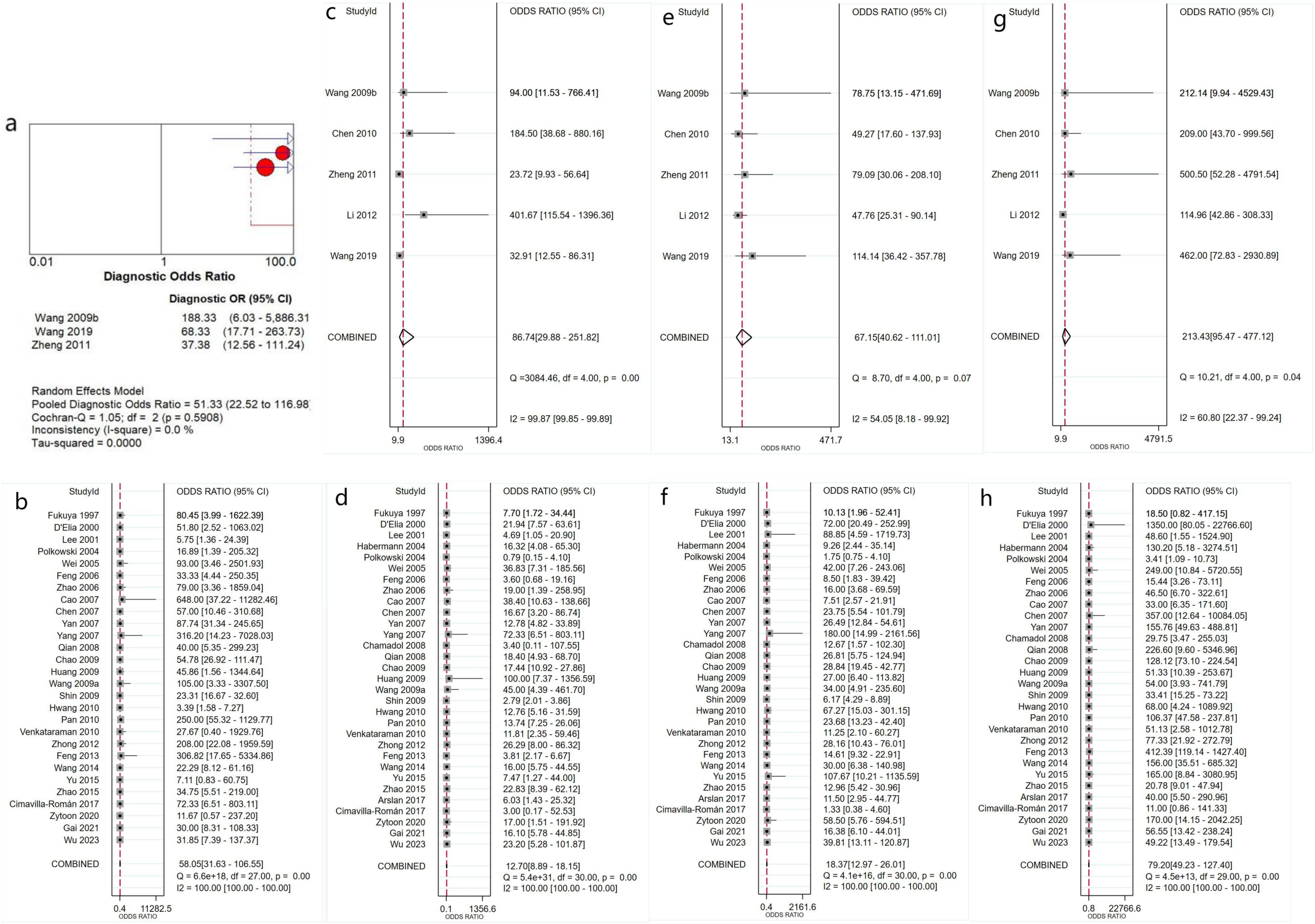

a
Diagnostic Odds Ratio
Diagnostic OR (95% CI)
Wang 2009b 188.33 (6.03 - 5,886.31
Wang 2019 68.33 (17.71 - 263.73)
Zheng 2011 37.38 (12.56 - 111.24)
Random Effects Model
Pooled Diagnostic Odds Ratio = 51.33 (22.52 to 116.98
Cochran-Q = 1.05; df = 2 (p = 0.5908)
Inconsistency (I-square) = 0.0 %
Tau-squared = 0.0000
b
c
d
e
f
g
h
StudyId
ODDS RATIO (95% CI)
COMBINED
ODDS RATIO

Supplementary Figure 8. Forest plots of DOR for DCEUS and enhanced-CT among SDTA

(a) DCEUS in T1 staging; (b) enhanced-CT in T1 staging; (c) DCEUS in T2 staging; (d) enhanced-CT in T2 staging; (e) DCEUS in T3 staging; (f) enhanced-CT in T3 staging; (g) DCEUS in T4 staging; (h) enhanced-CT in T4 staging. DOR, Diagnostic Odds Ratio.

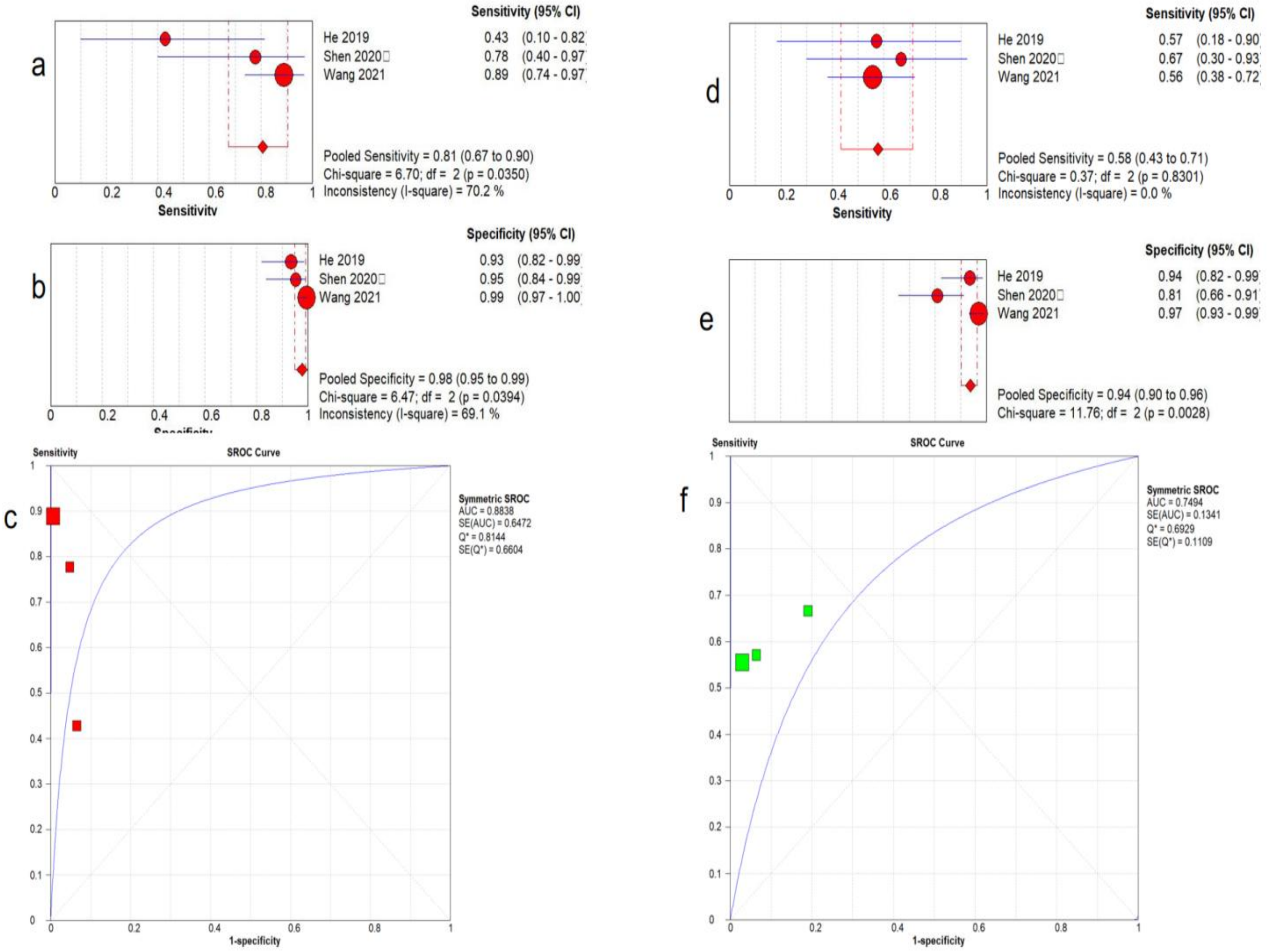

Supplementary Figure 9. Forest plots of sensitivity, specificity and SROC for DCEUS and enhanced-CT in T2 staging CDTA

SE (a), SP (b), and SROC (c) for DCEUS

SE (d), SP (e), and SROC (f) for enhanced-CT

SE: sensitivity; SP: specificity; CT, computed tomography; DCEUS, double contrast-enhanced ultrasound; comparative diagnostic test accuracy, comparative diagnostic test accuracy studies

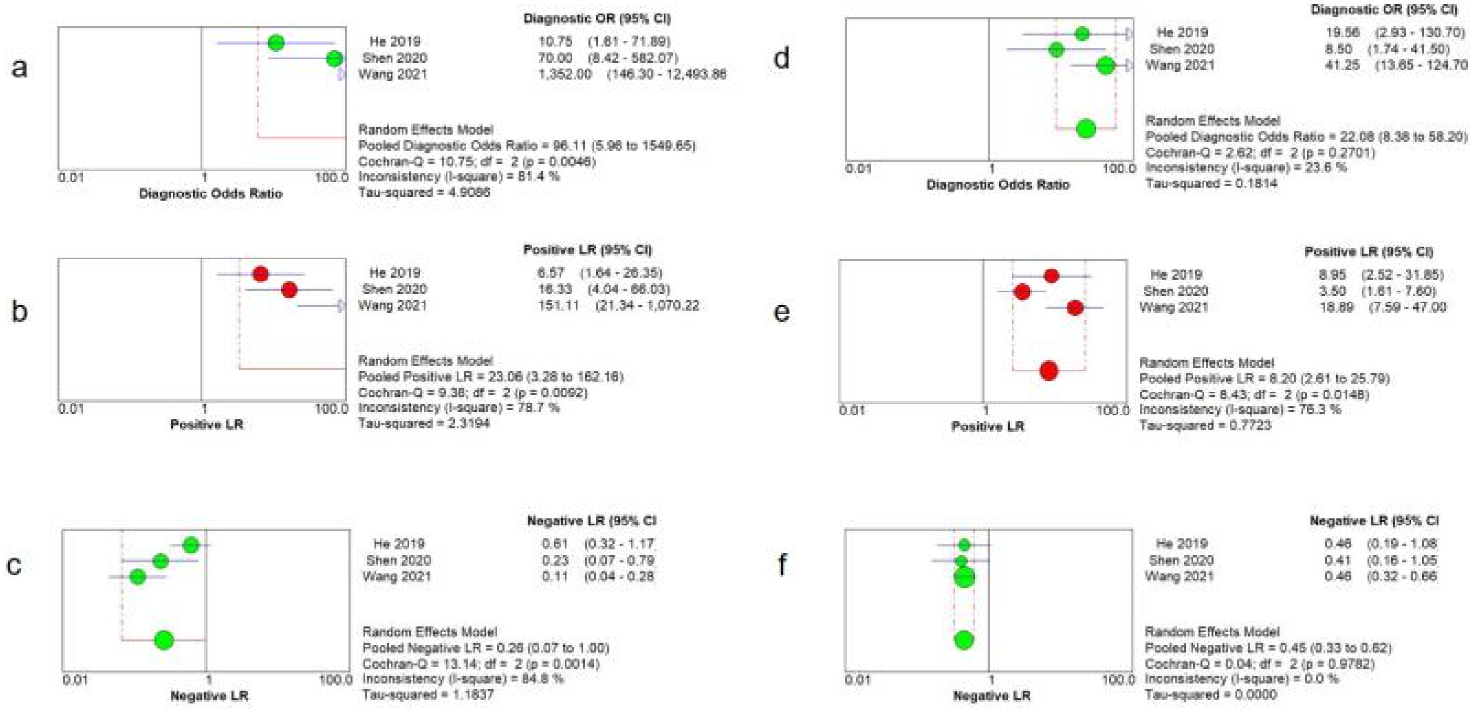

Supplementary Figure 10. Forest plots of DOR, PLR and NLR for DCEUS and enhanced-CT in T2 staging CDTA

DOR (a), PLR (b), and NLRC (c) for DCEUS

DOR (d), PLR (e), and NLRC (f) for enhanced-CT

DOR: diagnostic odds ratio; PLR: positive likelihood ratio; NLR: negative likelihood ratio; CT, computed tomography; DCEUS, double contrast-enhanced ultrasound

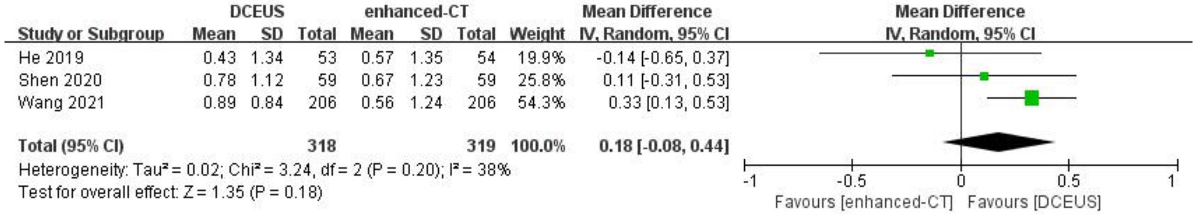

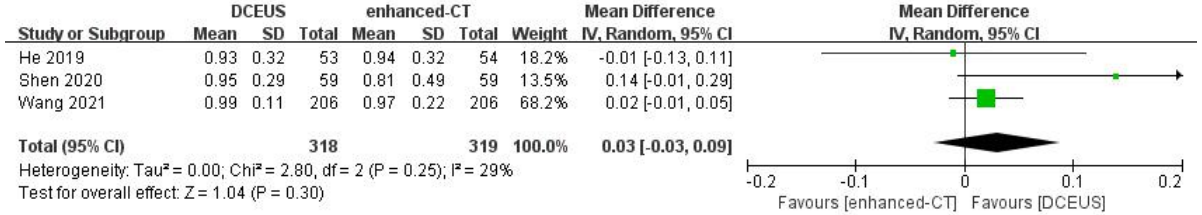

Supplementary Figure 11. Forest plots of MD for sensitivity and specificity in T2 staging CDTA sensitivity (A), specificity (B)

MD, mean difference; CI, confidence interval; comparative diagnostic test accuracy, comparative diagnostic test accuracy studies

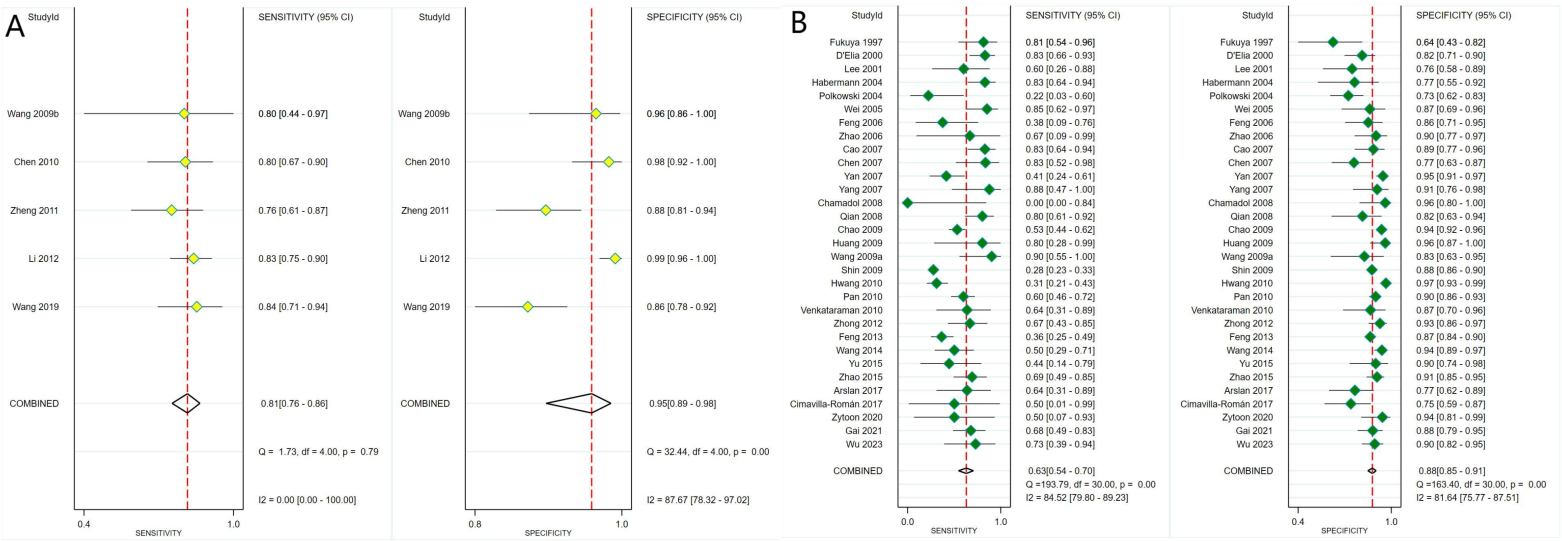

Supplementary Figure 12. Forest plots of sensitivity and specificity for DCEUS and enhanced-CT in T2 staging among SDTA

DCEUS (A), enhanced-CT (B)

CT, computed tomography; DCEUS, double contrast-enhanced ultrasound; comparative diagnostic test accuracy, comparative diagnostic test accuracy studies

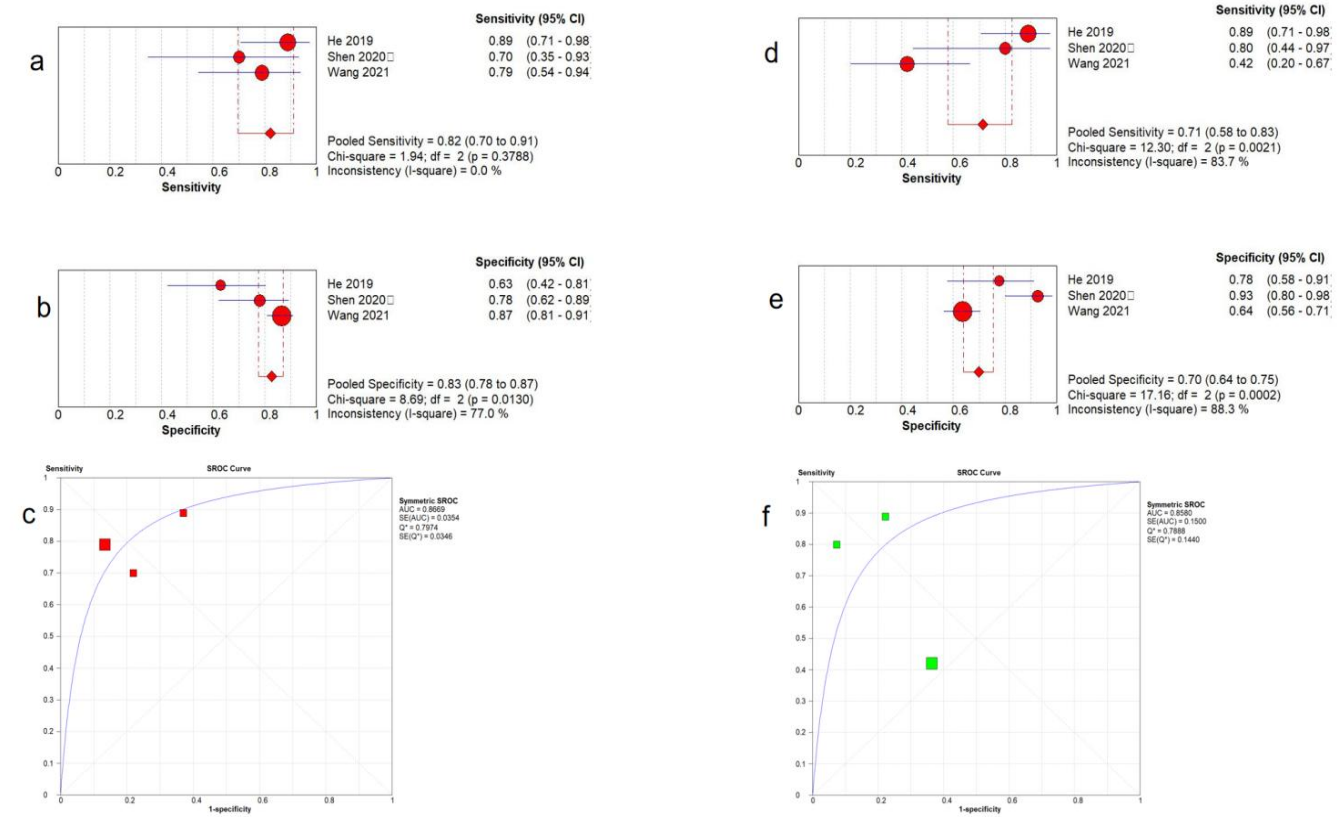

Supplementary Figure 13. Forest plots of sensitivity, specificity and SROC for DCEUS and enhanced-CT in T3 staging CDTA

SE (a), SP (b), and SROC (c) for DCEUS

SE (d), SP (e), and SROC (f) for enhanced-CT

SE: sensitivity; SP: specificity; CT, computed tomography; DCEUS, double contrast-enhanced ultrasound; comparative diagnostic test accuracy, comparative diagnostic test accuracy studies

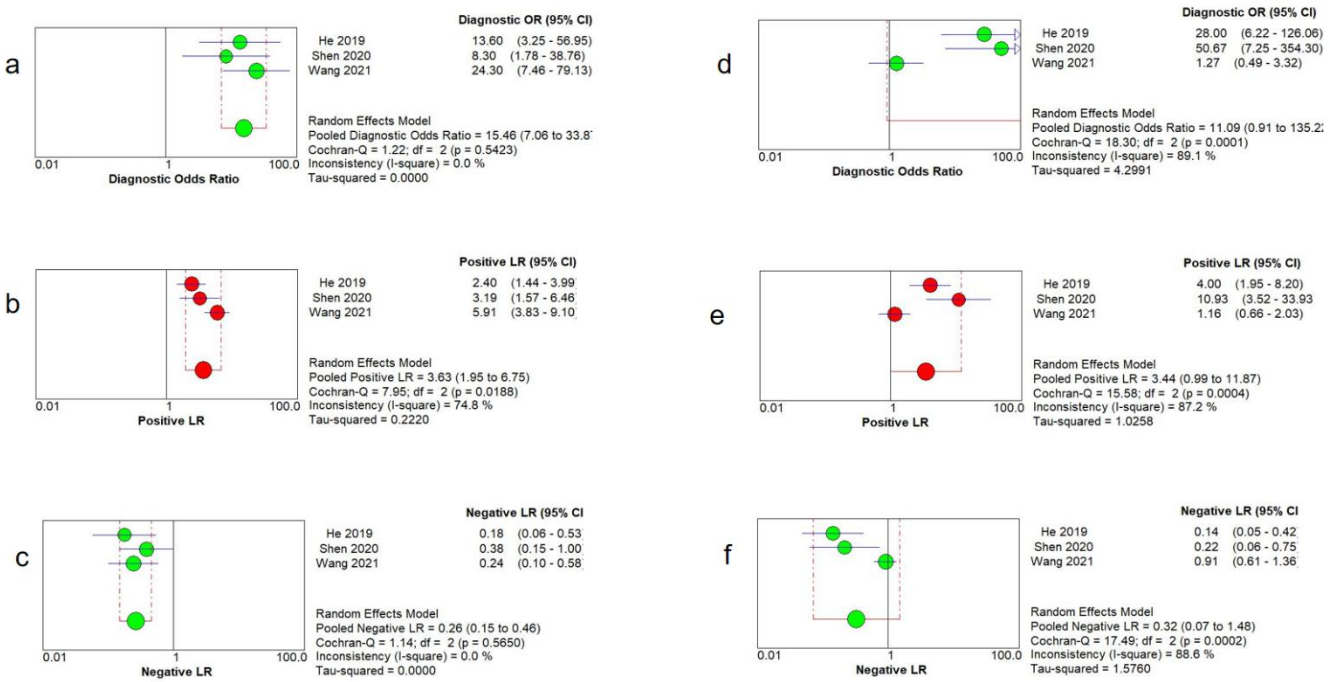

Supplementary Figure 14. Forest plots of DOR, PLR and NLR for DCEUS and enhanced-CT in T3 staging CDTA

DOR (a), PLR (b), and NLRC (c) for DCEUS

DOR (d), PLR (e), and NLRC (f) for enhanced-CT

DOR: diagnostic odds ratio; PLR: positive likelihood ratio; NLR: negative likelihood ratio; CT, computed tomography; DCEUS, double contrast-enhanced ultrasound; comparative diagnostic test accuracy, comparative diagnostic test accuracy studies

Study or Subgroup	DCEUS Mean	DCEUS SD	DCEUS Total	enhanced-CT Mean	enhanced-CT SD	enhanced-CT Total	Weight	Mean Difference IV, Random, 95% CI
He 2019	0.89	0.51	54	0.89	0.51	54	43.5%	0.00 [-0.19, 0.19]
Shen 2020	0.7	1.14	59	0.8	1.04	59	24.8%	-0.10 [-0.49, 0.29]
Wang 2021	0.79	1.46	206	0.42	1.72	206	31.7%	0.37 [0.06, 0.68]
**Total (95% CI)**			**319**			**319**	**100.0%**	**0.09 [-0.17, 0.35]**

Heterogeneity: Tau² = 0.03; Chi² = 4.86, df = 2 (P = 0.09); I² = 59%
Test for overall effect: Z = 0.70 (P = 0.49)

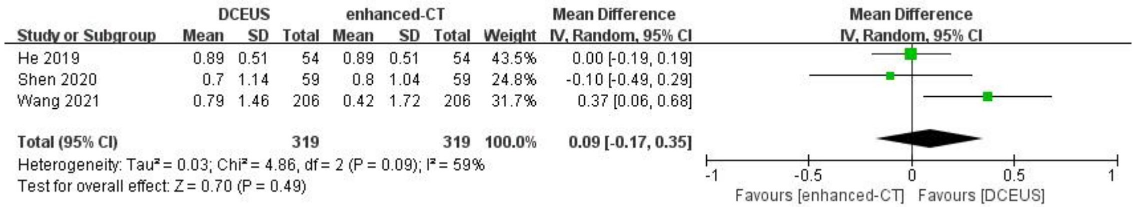

Study or Subgroup	DCEUS Mean	DCEUS SD	DCEUS Total	enhanced-CT Mean	enhanced-CT SD	enhanced-CT Total	Weight	Mean Difference IV, Random, 95% CI
He 2019	0.63	0.73	54	0.78	0.62	54	29.5%	-0.15 [-0.41, 0.11]
Shen 2020	0.78	0.53	59	0.93	0.35	59	34.0%	-0.15 [-0.31, 0.01]
Wang 2021	0.87	0.37	206	0.64	0.55	206	36.5%	0.23 [0.14, 0.32]
**Total (95% CI)**			**319**			**319**	**100.0%**	**-0.01 [-0.31, 0.28]**

Heterogeneity: Tau² = 0.06; Chi² = 20.60, df = 2 (P < 0.0001); I² = 90%
Test for overall effect: Z = 0.07 (P = 0.94)

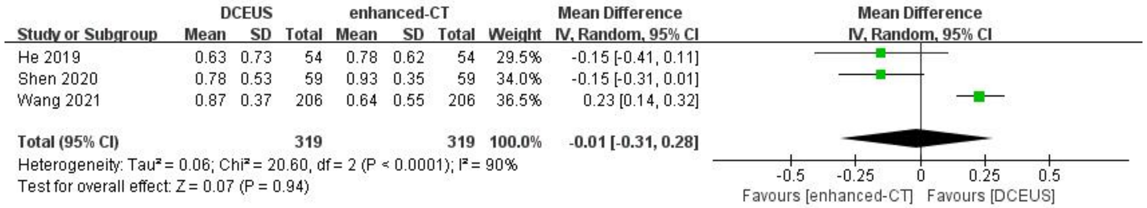

Supplementary Figure 15. Forest plots of MD for sensitivity and specificity in T3 staging CDTA sensitivity (A), specificity (B)

MD, mean difference; CI, confidence interval; comparative diagnostic test accuracy, comparative diagnostic test accuracy studies

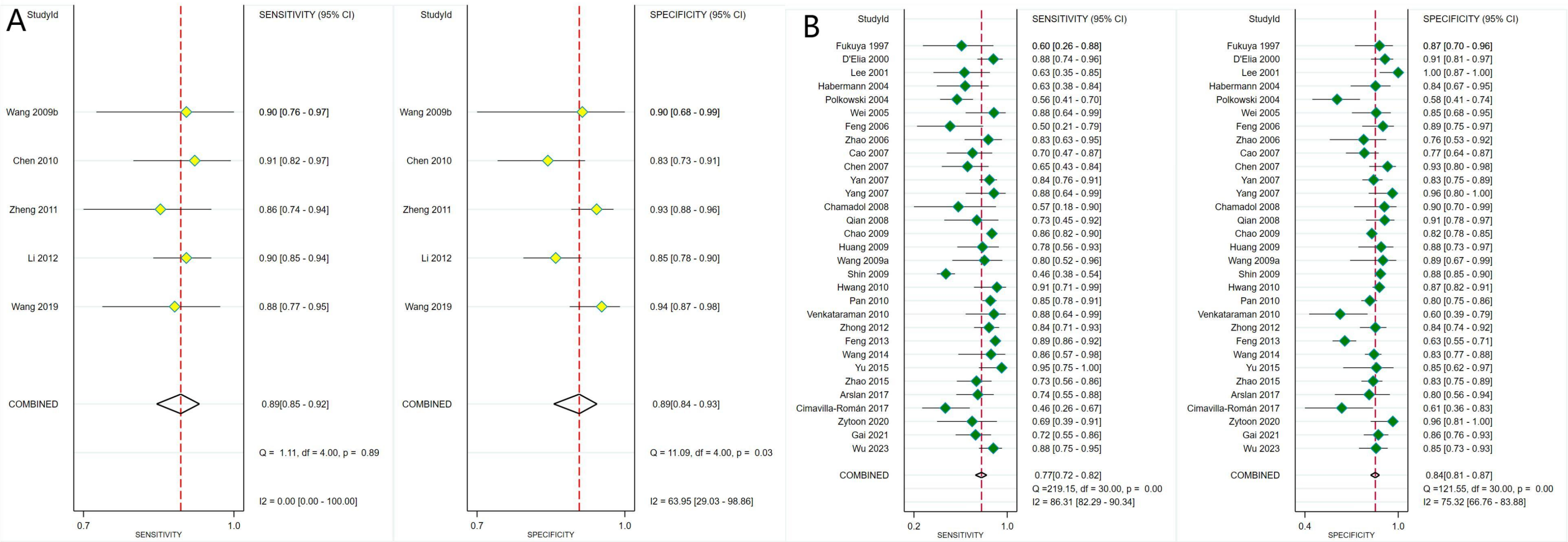

Supplementary Figure 16. Forest plots of sensitivity and specificity for DCEUS and enhanced-CT in T3 staging among SDTA

DCEUS (A), enhanced-CT (B)

CT, computed tomography; DCEUS, double contrast-enhanced ultrasound; SDTA, single diagnostic test accuracy studies

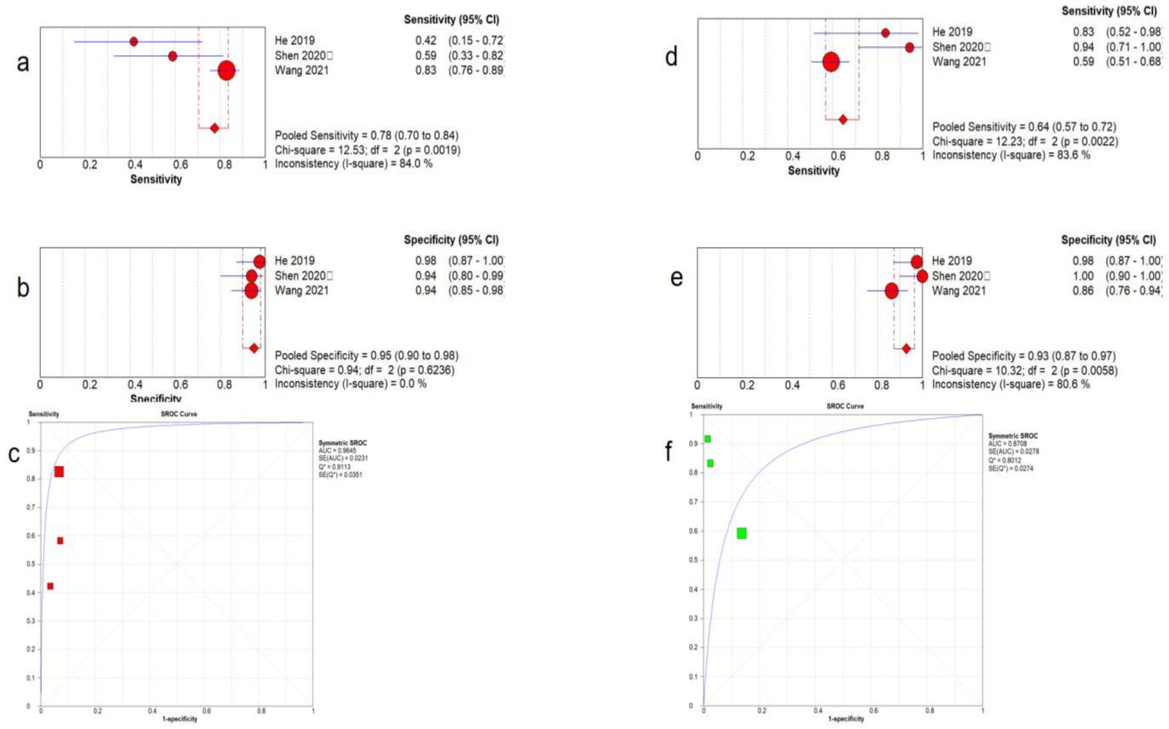

Supplementary Figure 17. Forest plots of sensitivity, specificity and SROC for DCEUS and enhanced-CT in T4 staging CDTA

SE (a), SP (b), and SROC (c) for DCEUS

SE (d), SP (e), and SROC (f) for enhanced-CT

SE: sensitivity; SP: specificity; CT, computed tomography; DCEUS, double contrast-enhanced ultrasound; comparative diagnostic test accuracy, comparative diagnostic test accuracy studies

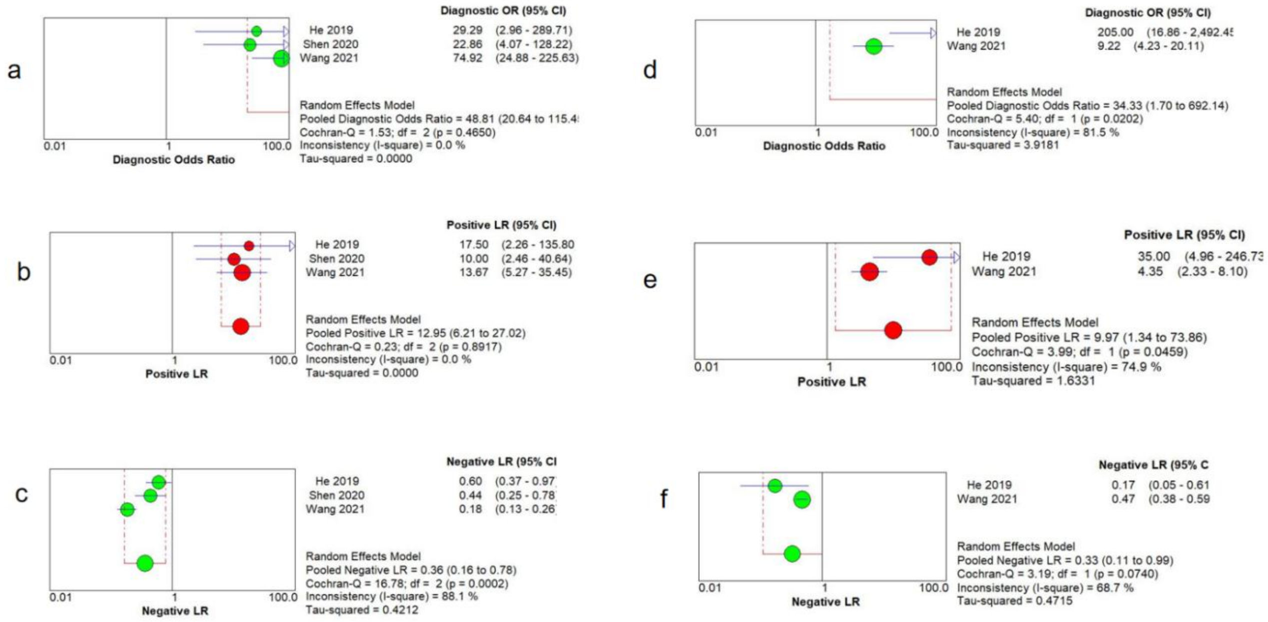

Supplementary Figure 18. Forest plots of DOR, PLR and NLR for DCEUS and enhanced-CT in T4 staging CDTA

DOR (a), PLR (b), and NLRC (c) for DCEUS

DOR (d), PLR (e), and NLRC (f) for enhanced-CT

DOR: diagnostic odds ratio; PLR: positive likelihood ratio; NLR: negative likelihood ratio; CT, computed tomography; DCEUS, double contrast-enhanced ultrasound; comparative diagnostic test accuracy, comparative diagnostic test accuracy studies

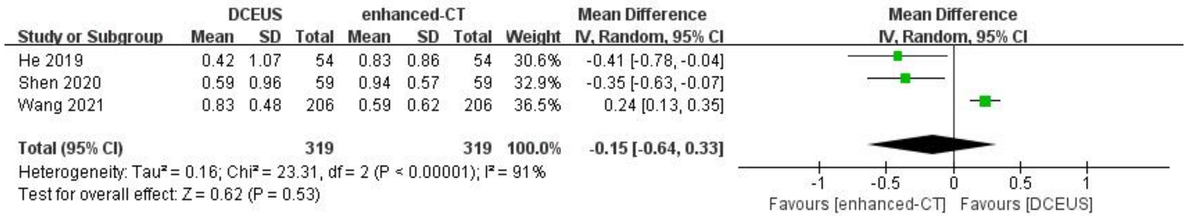

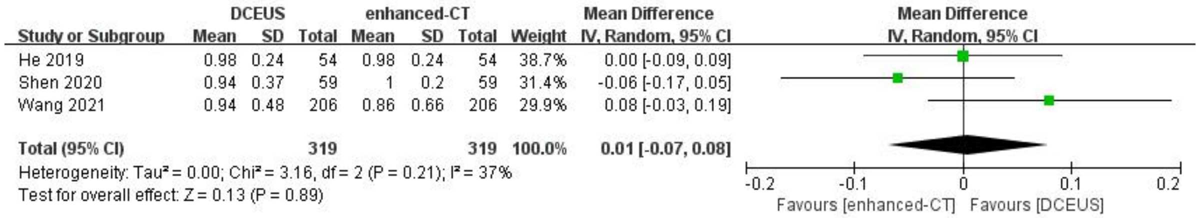

Supplementary Figure 19. Forest plots of MD for sensitivity and specificity in T4 staging CDTA sensitivity (A), specificity (B)

MD, mean difference; CI, confidence interval; comparative diagnostic test accuracy, comparative diagnostic test accuracy studies

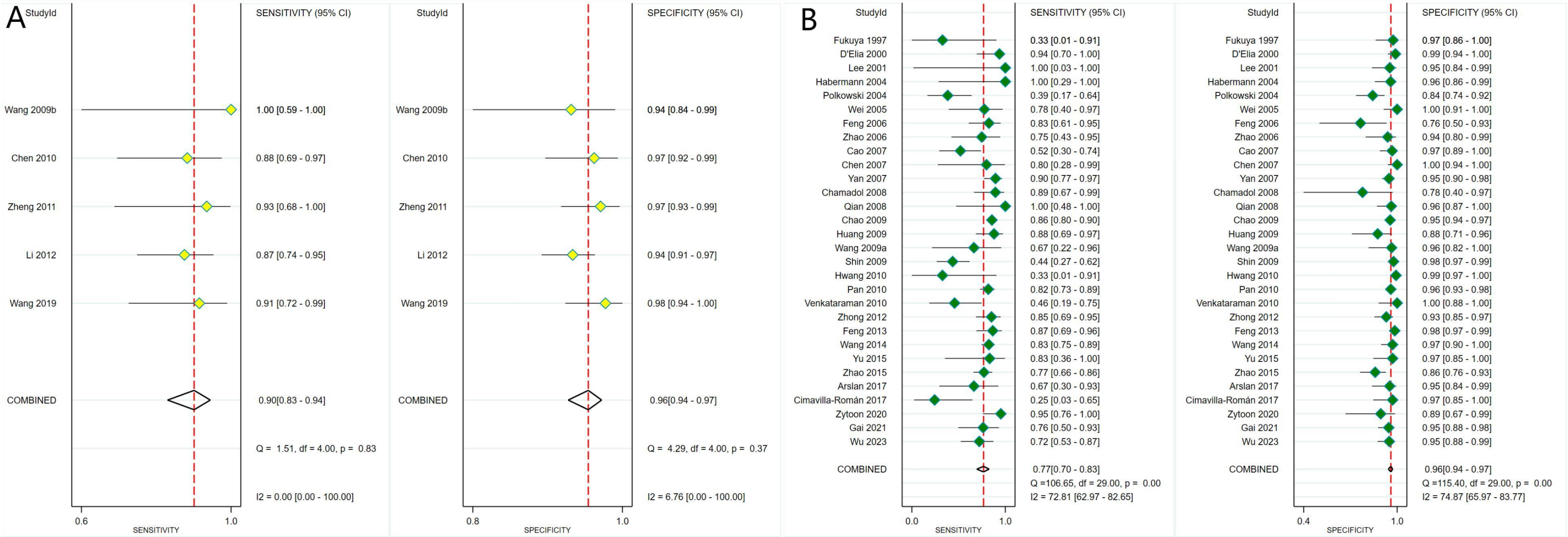

Supplementary Figure 20. Forest plots of sensitivity and specificity for DCEUS and enhanced-CT in T4 staging among SDTA DCEUS (A), enhanced-CT (B)

CT, computed tomography; DCEUS, double contrast-enhanced ultrasound; SDTA, Single diagnostic test accuracy studies

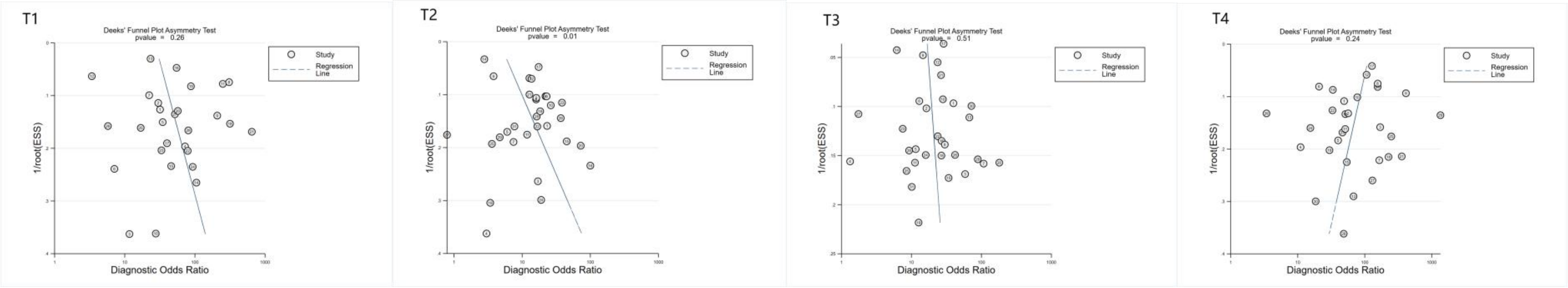

Supplementary Figure 21. The results of Deek's funnel plot tes

Reference

1. Schünemann HJ, Mustafa RA, Brozek J, Santesso N, Bossuyt PM, Steingart KR, et al. GRADE guidelines: 22. The GRADE approach for tests and strategies-from test accuracy to patient-important outcomes and recommendations. J Clin Epidemiol. 2019;111:69-82.
2. Hultcrantz M, Mustafa RA, Leeflang MMG, Lavergne V, Estrada-Orozco K, Ansari MT, et al. Defining ranges for certainty ratings of diagnostic accuracy: a GRADE concept paper. J Clin Epidemiol. 2020;117:138-48.
3. Whiting PF, Rutjes AW, Westwood ME, Mallett S, Deeks JJ, Reitsma JB, et al. QUADAS-2: a revised tool for the quality assessment of diagnostic accuracy studies. Ann Intern Med. 2011;155(8):529-36.
4. MS. P. The Statistical Evaluation of Medical Tests for Classifi- cation and Prediction. Oxford University Press. 2003.
5. Schünemann HJ, Mustafa RA, Brozek J, Steingart KR, Leeflang M, Murad MH, et al. GRADE guidelines: 21 part 2. Test accuracy: inconsistency, imprecision, publication bias, and other domains for rating the certainty of evidence and presenting it in evidence profiles and summary of findings tables. J Clin Epidemiol. 2020;122:142-52.
6. Yang B, Mustafa RA, Bossuyt PM, Brozek J, Hultcrantz M, Leeflang MMG, et al. GRADE Guidance: 31. Assessing the certainty across a body of evidence for comparative test accuracy. J Clin Epidemiol. 2021;136:146-56.
7. Schünemann HJ, Mustafa RA, Brozek J, Steingart KR, Leeflang M, Murad MH, et al. GRADE guidelines: 21 part 1. Study design, risk of bias, and indirectness in rating the certainty across a body of evidence for test accuracy. J Clin Epidemiol. 2020;122:129-41.
8. Wu LL, Xin JY, Wang JJ, Feng QQ, Xu XL, Li KY. Prospective Comparison of Oral Contrast-Enhanced Transabdominal Ultrasound Imaging With Contrast-Enhanced Computed Tomography in Pre-operative Tumor Staging of Gastric Cancer. Ultrasound in Medicine and Biology. 2023;49(2):569-77.
9. Wang J, Li X, Zhang Z, Jing C, Li J. Clinical Research of Combined Application of DCEUS and Dynamic Contrast-Enhanced MSCT in Preoperative cT Staging of Gastric Cancer. Journal of Oncology. 2021;2021.
10. Gai Q, Li X, Li N, Li L, Meng Z, Chen A. Clinical significance of multi-slice spiral CT, MRI combined with gastric contrast-enhanced ultrasonography in the diagnosis of T staging of gastric cancer. Clin Transl Oncol. 2021;23(10):2036-45.
11. Zytoon AA, El-Atfey SIB, Hassanein SAH. Diagnosis of gastric cancer by MDCT gastrography: diagnostic characteristics and management potential. Egyptian Journal of Radiology and Nuclear Medicine. 2020;51(1).
12. Shen WF, Zhou HL, Li Y. Contrast-enhanced CT vs double contrast-enhanced ultrasound for preoperative evaluation of T stage of gastric cancer. World Chinese Journal of Digestology. 2020;28(20):1042-7.
13. Wang L, Liu Z, Kou H, He H, Zheng B, Zhou L, et al. Double contrast-enhanced ultrasonography in preoperative T staging of gastric cancer: A comparison with

endoscopic ultrasonography. Frontiers in Oncology. 2019;9.
14. He P, Miao L-y, Ge H-y, Wang T-l, Ye J-x, Meng L-m, et al. Preoperative Tumor Staging of Gastric Cancer: Comparison of Double Contrast-Enhanced Ultrasound and Multidetector Computed Tomography. Journal of Ultrasound in Medicine. 2019;38(12):3203-9.
15. Cimavilla Román M, de la Serna Higuera C, Loza Vargas LA, Benito Fernández C, Barrio Andrés J, Madrigal Rubiales B, et al. Endoscopic ultrasound versus multidetector computed tomography in preoperative gastric cancer staging. Rev Esp Enferm Dig. 2017;109(11):761-7.
16. Arslan H, Fatih Özbay M, Çalll I, Doğan E, Çelik S, Batur A, et al. Contribution of diffusion weighted MRI to diagnosis and staging in gastric tumors and comparison with multi-detector computed tomography. Radiology and Oncology. 2017;51(1):23-9.
17. Zhao Q, Li Y, Hu Z, Tan B, Yang P, Tian Y. Value of the preoperative TNM staging and the longest tumor diameter measurement of gastric cancer evaluated by MSCT. Zhonghua wei chang wai ke za zhi = Chinese journal of gastrointestinal surgery. 2015;18(3):227-31.
18. Yu T, Wang X, Zhao Z, Liu F, Liu X, Zhao Y, et al. Prediction of T stage in gastric carcinoma by enhanced CT and oral contrast-enhanced ultrasonography. World Journal of Surgical Oncology. 2015;13(1).
19. Wang Y, Wang J, Hong Q. Joint application of computed tomography and laparoscopy in preoperative staging of gastric cancer. National Medical Journal of China. 2014;94(10):769-71.
20. Feng XY, Wang W, Luo GY, Wu J, Zhou ZW, Li W, et al. Comparison of endoscopic ultrasonography and multislice spiral computed tomography for the preoperative staging of gastric cancer - Results of a single institution study of 610 chinese patients. PLoS ONE. 2013;8(11).
21. Zhong BY, Liu YX, Huang WF, Liu QQ, Liu SQ, Liu Y. [Clinical value of 64-slice spiral 3-phase CT enhanced scanning for preoperative TNM staging assessment of gastric carcinoma]. Zhonghua wei chang wai ke za zhi = Chinese journal of gastrointestinal surgery. 2012;15(7):706-9.
22. Li S, Huang P, Wang Z, Chen J, Xu H, Wang L, et al. Preoperative T staging of advanced gastric cancer using double contrast-enhanced ultrasound. Ultraschall in der Medizin (Stuttgart, Germany : 1980). 2012;33(7):E218-24.
23. Zheng Z, Yu Y, Lu M, Sun W, Wang F, Li P, et al. Double contrast-enhanced ultrasonography for the preoperative evaluation of gastric cancer: A comparison to endoscopic ultrasonography with respect to histopathology. American Journal of Surgery. 2011;202(5):605-11.
24. Venkataraman I, Rao HK, Singh P, Elangovan S, Kate V. Efficacy of Hydrogastric Sonography and Spiral Computed Tomography in Staging of Gastric Carcinoma-A Comparative Study. Journal of Clinical Ultrasound. 2010;38(9):480-5.
25. Pan Z, Zhang H, Yan C, Du L, Ding B, Song Q, et al. Determining gastric cancer resectability by dynamic MDCT. European Radiology. 2010;20(3):613-20.
26. Hwang SW, Lee DH, Lee SH, Park YS, Hwang JH, Kim JW, et al. Preoperative staging of gastric cancer by endoscopic ultrasonography and multidetector-row computed tomography. Journal of Gastroenterology and Hepatology (Australia). 2010;25(3):512-8.

27. Chen RJ, Huang PT, Li YP, Zheng ZQ, Zhao YP, Huang FG, et al. [Comparison of preoperative T staging by oral contrast enhanced ultrasonography and double contrast enhanced ultrasonography in advanced gastric carcinoma]. Zhonghua zhong liu za zhi [Chinese journal of oncology]. 2010;32(7):551-4.
28. Kun-wook SRLJ-hLM-sPJIKH-hYH-kL. Analysis of Diagnostic Performance of CT and EUS for Clinical TN Staging of Gastric Cancer. Journal of Gastric Cancer. 2009;9(4):177-85.
29. Wang G, Cheng X, Wang Z, Lei Z, Zhang X. Diagnotic value of MSCT with two oral contrast agents filling method in TNM staging of gastric carcinoma. Chinese Journal of Clinical Oncology. 2009;36(22):1266-70.
30. Wang CL, Yang YM, Cui J, Ouyang H, Wang ZM, Ye HS, et al. [Diagnostic value of double contrast-enhanced ultrasonography in preoperative staging of gastric cancer]. Zhonghua zhong liu za zhi [Chinese journal of oncology]. 2009;31(9):701-4.
31. Huang J, Chen WX, Wang XP, Yao J. Clinical application of mutidetector computer tomography in diagnosing T staging of gastric carcinoma. Journal of Sichuan University (Medical Science Edition). 2009;40(6):1105-9.
32. Chao Y, Zhu ZG, Yan M, Zhang H, Pan ZL, Chen J, et al. Value of multidetector-row computed tomography in the preoperative T and N staging of gastric carcinoma: A large-scale Chinese study. Journal of Surgical Oncology. 2009;100(3):205-14.
33. Qian J, Ren K, Wang Q, Lu T, Zhu YS, Zhao Y, et al. Comparative study of scanning methods of multiple detector-row spiral computed tomography to the advanced gastric cancer: Different scanning stages. Chinese Journal of Medical Imaging Technology. 2008;24(5):710-3.
34. Chamadol N, Wongwiwatchai J, Bhudhisawasd V, Pairojkul C. Accuracy of spiral CT in preoperative staging of gastric carcinoma: Correlation with surgical and pathological findings. Journal of the Medical Association of Thailand. 2008;91(3):356-63.
35. Yang DM, Kim HC, Jin W, Ryu CW, Kang JH, Park CH, et al. 64 multidetector-row computed tomography for preoperative evaluation of gastric cancer: histological correlation. J Comput Assist Tomogr. 2007;31(1):98-103.
36. Yan C, Zhu ZG, Yan M, Chen KM, Chen J, Liu BY, et al. Clinical significance of multi-slice spiral CT and serum tumor markers in the preoperative assessment of gastric carcinoma. World Chinese Journal of Digestology. 2007;15(30):3194-203.
37. Chen BB, Liang PC, Liu KL, Hsiao JK, Huang JC, Wong JM, et al. Preoperative diagnosis of gastric tumors by three-dimensional multidetector row CT and double contrast barium meal study: Correlation with surgical and histologic results. Journal of the Formosan Medical Association. 2007;106(11):943-52.
38. Cao H, Liu H, Lu MH, Chen ZP, Wu ZY, Gong HX, et al. Clinical application of multi-slice spiral computed tomography in the preoperative staging of gastric carcinoma. Zhonghua wei chang wai ke za zhi = Chinese journal of gastrointestinal surgery. 2007;10(4):346-9.
39. Zhao ZQ, Zheng KG, Xu DS. Value of spiral computed tomography in preoperative TNM staging for gastric cancer. World Chinese Journal of Digestology. 2006;14(18):1785-9.

40. Feng Q, Zhuang ZG, Xu JR. Multislice CT for diagnosis of invasion depth of gastric cancer. Chinese Journal of Medical Imaging Technology. 2006;22(2):257-9.
41. Wei WZ, Yu JP, Li J, Liu CS, Zheng XH. Evaluation of contrast-enhanced helical hydro-CT in staging gastric cancer. World Journal of Gastroenterology. 2005;11(29):4592-5.
42. Polkowski M, Palucki J, Wronska E, Szawlowski A, Nasierowska-Guttmejer A, Butruk E. Endosonography versus helical computed tomography for locoregional staging of gastric cancer. Endoscopy. 2004;36(7):617-23.
43. Habermann CR, Weiss F, Riecken R, Honarpisheh H, Bohnacker S, Staedtler C, et al. Preoperative staging of gastric adenocarcinoma: comparison of helical CT and endoscopic US. Radiology. 2004;230(2):465-71.
44. Lee DH, Ko YT, Park SJ, Lim JW. Comparison of hydro-US and spiral CT in the staging of gastric cancer. Clinical Imaging. 2001;25(3):181-6.
45. D'Elia F, Zingarelli A, Palli D, Grani M. Hydro-dynamic CT preoperative staging of gastric cancer: Correlation with pathological findings. A prospective study of 107 cases. European Radiology. 2000;10(12):1877-85.
46. Fukuya T, Honda H, Kaneko K, Kuroiwa T, Yoshimitsu K, Irie H, et al. Efficacy of helical CT in T-staging of gastric cancer. Journal of Computer Assisted Tomography. 1997;21(1):73-81.
